# Vulnerability to snakebite envenoming and access to healthcare in the Terai region of Nepal: a geospatial analysis

**DOI:** 10.1016/j.lansea.2022.100103

**Published:** 2022-11-17

**Authors:** Carlos Ochoa, Mamit Rai, Sara Babo Martins, Gabriel Alcoba, Isabelle Bolon, Rafael Ruiz de Castañeda, Sanjib Kumar Sharma, François Chappuis, Nicolas Ray

**Affiliations:** a Institute of Global Health (IGH), Department of Community Health and Medicine, Faculty of Medicine, University of Geneva, Geneva, Switzerland; b Institute for Environmental Sciences (ISE), University of Geneva, Geneva, Switzerland; c KHDC-Nepal, Dharan, Nepal; d Médecins Sans Frontières (MSF), Neglected Tropical Diseases Working Group, Geneva, Switzerland; e Division of Tropical and Humanitarian Medicine, Geneva University Hospitals (HUG), Geneva, Switzerland; f B.P. Koirala Institute of Health Sciences (BPKIHS), Dharan, Nepal; g Department of Community Health and Medicine, Faculty of Medicine, University of Geneva, Geneva, Switzerland

**Keywords:** Snakebite, Vulnerability, Accessibility to healthcare, Travel time, Nepal, Risk, Geospatial analysis, AccessMod

## Abstract

**Background:**

Snakebite envenoming is a neglected tropical disease that mainly affects poor populations in rural areas. In hyperendemic regions, prevention could partially reduce the constant risk, but the population still needs timely access to adequate treatment. In line with WHO's snakebite roadmap, we aim to understand snakebite vulnerability through modelling of risk and access to treatment, and propose plausible solutions to optimise resource allocation.

**Methods:**

We combined snakebite-risk distribution rasters with travel-time accessibility analyses for the Terai region of Nepal, considering three vehicle types, two seasons, two snakebite syndromes, and uncertainty intervals. We proposed localised and generalised optimisation scenarios to improve snakebite treatment coverage for the population, focusing on the neurotoxic syndrome.

**Findings:**

In the Terai, the neurotoxic syndrome is the main factor leading to high snakebite vulnerability. For the most common scenario of season, syndrome, and transport, an estimated 2.07 (15.3%) million rural people fall into the high vulnerability class. This ranges between 0.3 (2.29%) and 6.8 (50.43%) million people when considering the most optimistic and most pessimistic scenarios, respectively. If all health facilities treating snakebite envenoming could optimally treat both syndromes, treatment coverage of the rural population could increase from 65.93% to 93.74%, representing a difference of >3.8 million people.

**Interpretation:**

This study is the first high-resolution analysis of snakebite vulnerability, accounting for uncertainties in both risk and travel speed. The results can help identify populations highly vulnerable to snakebite envenoming, optimise resource allocation, and support WHO's snakebite roadmap efforts.

**Funding:**

10.13039/501100001711Swiss National Science Foundation.

**Research in context**

**Evidence before this study**

Understanding vulnerability to snakebite is key to effectively design health policies. Previous studies looking at the distribution of snakebite risk or at vulnerability have assumed multiple proxies, analysed only one season, one mode of transport or one snakebite syndrome, or generalised their analysis to include any health conditions and facility type. Recent research has identified hotspots of snakebite risk in specific parts of the Terai and integrated the importance of poverty, environmental, and climatic factors in assessing snakebite risk into a One Health framework. To date, no study has combined high-resolution snakebite risk based on large-scale community surveys with multifactorial accessibility to snakebite envenoming (SBE) treatment to determine vulnerability.

**Added value of this study**

This study overcomes the limitations of previous work by building on our previous findings on snakebite risk and providing realistic estimates of travel time to SBE treatment for different combinations of common transport methods, seasons, and snakebite syndromes. Combining geospatial data on travel time to healthcare and snakebite risk resulted in high-resolution maps of population vulnerability to SBE in the Terai, including uncertainty intervals in key parameters, which provide a range of potential results and avoids underestimating the vulnerable population. Highly vulnerable populations are identified and scenarios for scaling-up SBE treatment capacity are proposed, providing concrete policymaking input.

**Implications of all the available evidence**

By combining two fundamental elements that determine the outcome of SBE events, namely the risk of snakebite and the accessibility to treatment, the vulnerability of the population to adverse outcomes of SBE has now been mapped at high spatial resolution, emphasizing particularly high population vulnerability in the eastern part of the Terai. Local transport methods, snakebite syndromes, and seasonal conditions were considered, and possible variations due to uncertainties were included, all in a realistic approach aiming to represent real-life conditions as accurately as possible. The results can support decision-makers and public health planners by providing a balanced view of vulnerability depending on SBE syndrome (haemotoxic or neurotoxic), season, and the type of transport victims use to reach medical care. This has implications for the appropriate allocation of resources and training of healthcare providers in snakebite management, as well as for preventive measures against snakebite where they are most needed. The methodology can serve as a blueprint for other SBE hyperendemic countries and make a meaningful contribution towards halving the number of deaths by 2030, a key target of the WHO roadmap for snakebite.

## Introduction

Snakebite envenoming (SBE) is a multifaceted neglected tropical disease that primarily affects underserved and poor communities in low- and middle-income countries (LMIC).^1^ Realistically, it cannot be eradicated because the causative agents, venomous snakes, represent hundreds of species with an extremely important ecological role and a global distribution. SBE is estimated to cause up to 138,000 human deaths and more than 400,000 disabilities worldwide each year.^1^ In the Terai region of Nepal, the latest incidence reaches 251 victims per 100,000 people, representing an estimated 36,148 snakebite victims in the Terai's rural population per year,^2^ an alarmingly high value comparable to estimates in other hyperendemic countries in the region (Bangladesh 623.4 and Sri Lanka 398 per 100,000).^3^^,^^4^ Crucially, quick access to appropriate medical treatment should reduce fatal outcome or extreme morbidity.

Snakebite risk has been defined as the theoretical probability of getting bitten by a (venomous) snake.^5^ For the analysis of snakebite risk, multiple factors such as variations of temperature and precipitation, urbanization, or distance to water have been considered according to specific conditions of the study area.^3^^,^^6^^,^^7^ In a previous study, we geographically modelled snakebite risk per year, with a resolution of 1 km (see Methods), identified the hotspots of snakebite risk along the Terai, and highlighted the multifactorial nature of SBE, which depends on environmental, ecological, socio-economic, and geospatial factors, among others.^6^

In a health framework, vulnerability includes the conditions determined by physical, socio-economic, and environmental factors that increase the susceptibility of an individual or community to the impact of hazards. We adopt this in our estimation of SBE vulnerability. In the Terai, there are two main types of life-threatening SBE systemic syndromes: neurotoxic and haemotoxic, depending on which tissues are mainly affected. The neurotoxic syndrome is the most urgent to treat, as severe manifestations of envenoming can develop within an hour or less after the bite.^8^ In the haemotoxic syndrome, severe manifestations can take six or more hours to appear, allowing more time to seek medical attention. In Nepal, bites from cobras (*Naja naja* and *Naja* *kaouthia*) and kraits (*Bungarus* sp.) are the most frequent cause of neurotoxic envenomings^9^ and the appraised distributions for these species include the whole Terai.^8^ Hospital- and community-based epidemiological studies on snakebite in the Terai, conducted at the local and regional levels, have shown the predominance of the neurotoxic syndrome.^2^^,^^9^, ^10^, ^11^

The WHO's snakebite roadmap stresses the importance of getting SBE victims to health facilities that can treat snakebite appropriately with snake antivenom (SAV) and ventilatory support in a timely manner.^1^ An eastern-Terai community survey found that 80% of SBE deaths occurred in the community or on the road due to various delays, the most important one being lack of transport.^11^ Also, shorter reporting times are known to mean better outcomes after SBE.^1^ In many cases, victims might delay seeking medical help waiting for symptoms to appear, or due to unawareness or traditional practices.^12^ For Nepal, Alirol and colleagues reported times from bite to treatment ranging between 30 min and 15 days, due notably to season, landscape, transport availability, and time of the day.^13^ All this renders fundamental the identification of populations at high risk of delayed access to healthcare, and the modelling of their accessibility to snakebite treatment as realistically as possible.

For other health emergencies (eg, obstetric complications), WHO has set a maximum travel time limit of 2 h to reach healthcare.^14^ For SBE, this value varies greatly depending on the snake species and the associated clinical syndrome, laying between 30 min and more than 6 h.^13^ For the neurotoxic syndrome, WHO recommends a maximum delay of 1 h between the bite and reaching the health facility.^15^ For the haemotoxic syndrome, studies in India and Brazil have shown that administration of SAV before 6 h significantly reduced the development of severe envenoming symptoms.^16^^,^^17^ A rare example of a precise estimate of the delay effect in haemotoxic envenoming in Nigeria indicated an additional 1.01% mortality per each extra hour of travel time.^18^

Analyses of accessibility to healthcare are being conducted in many LMICs countries, including Nepal.^19^^,^^20^ However, to our knowledge, only two studies have combined snakebite (risk or cases) and accessibility to healthcare in the population,^21^^,^^22^ and none have done it continuously or taken uncertainty intervals (UI, a measure representing dispersion from an estimate) into account when calculating travel times. Similarly, when calculating snakebite vulnerability, previous studies have used surrogate values for risk (eg, species habitat suitability), and have also not considered UI.^22^^,^^23^ Here we analyse vulnerability to SBE across the Terai by combining high-resolution rasters of snakebite risk^6^ and travel time to SBE treatment facilities. We consider mode of transport, syndrome type, and climatic seasons, and incorporate UI into our vulnerability analysis for both travel time and snakebite risk, which provides a broader range of potential outcomes, a wider knowledge-base for decision-making, and reduces the chances of underestimating the affected population. Finally, we propose a set of scale-up and optimisation measures for existing snakebite facilities as a viable solution to improve rural people's access to adequate and timely medical treatment for SBE in the most vulnerable areas of the Terai.

## Methods

### Study area

The Terai region of Nepal comprises mainly the southern, subtropical plains of the country, where most of the rural population of Nepal lives.^24^ This study focused on this region considering that rural and poorer sectors of the population are the most affected by lethal snakebite,^6^^,^^25^ and that incidence is much lower in the other two regions of Nepal (hills and Himalayas).^10^^,^^26^ For a detailed description of the population selection, see Alcoba and colleagues.^27^ For this analysis, we also excluded the largest metropolitan areas (>20 000 inhabitants), where snakebites are infrequent, and the population is in closer proximity to various health services.

### Data sources and preparation

**Fig. 1:**
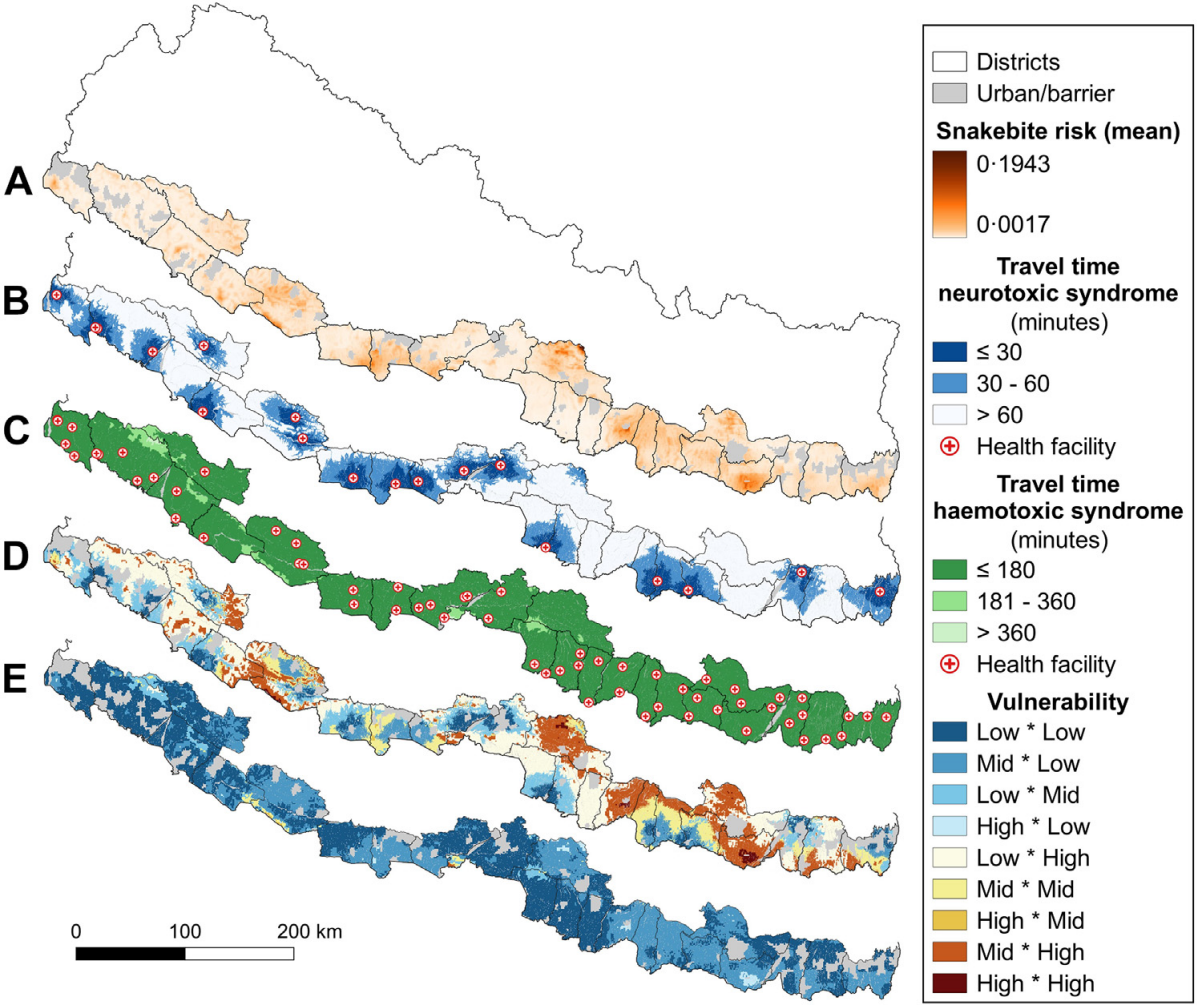
**From snakebite risk to vulnerability**. Terai maps of snakebite risk (mean posterior distribution at 1 km^2^/year, **a**),^6^ catchment areas of the facilities treating neurotoxic (**b**) and haemotoxic (**c**) syndromes, 9-class rasters of vulnerability to neurotoxic (**d**) and haemotoxic (**e**) syndromes. Scenarios (**b**–**e**) incorporate wet season and motorcycle as the main transport method. The combination of the reclassified versions of **a** × **b**, and **a** × **c** produce **d** and **e**, respectively. Source: vector map and administrative divisions from gadm.org, projected in the local WGS 84/UTM zone 45 N coordinate reference system in QGIS 3.24 (qgis.org).

Most of the data sources used in the study are open and freely available online (see Appendix 1 p 2 for sources and projection details). These include administrative units at different levels, landcover, road network, water elements, hanging trail bridges, health facilities, digital elevation model, and population layers. Additionally, we used the mean predicted snakebite risk (Fig. 1a) from Ochoa and colleagues^6^ (see Appendix 1 p 2) and the upper and lower UI rasters (mean ± SD) were derived. We emphasized the difference between climatic seasons by including additional smaller rivers and canals (dry for most of the year) to a separate wet-season waterline layer. We complemented the road network with a layer of hanging trail bridges,^28^ considering only bridges classified at the source as in good condition. We created a layer with health facilities treating SBE from open source layers and information from the Epidemiology and Disease Control Division in Nepal. An initial dataset of merged open source data and more than 1100 potential and confirmed snakebite treatment facilities was cleaned of duplicates and facilities unlikely to treat snakebite. Nepali investigators further cleansed the dataset through direct calls or visits to the facilities to identify the treatment options they offered. Accordingly, for this syndrome-focused accessibility analysis, we created two sets of facilities and performed separate analyses with them: 1) neurotoxic set, if the facilities have either mechanical respirators only, or respirators and SAV, as respirators are essential to treat severe cases of this syndrome; 2) haemotoxic set, if the facilities have either only SAV or respirators and SAV (see supplementary appendix 1). To produce the various plots and figures in the main document and in the supplementary material, and to calculate summary statistics, we use the statistical software R v.4.1.2.2.^29^

### Geographical accessibility analysis

Least cost path algorithms are particularly suitable to determine the distance or time to access healthcare in LMIC's conditions (eg, deficient roads and extensive barriers).^19^^,^^30^ These models use geospatial data to define movement through the landscape, include speed scenarios that incorporate expert-based information, and assume that motorized vehicles would be available without delay when a patient walks to the closest road. We computed travel time rasters between any location and SBE treating facilities using AccessMod (v.5.7.18).^30^ We created travel-speed scenarios (Appendix 1 Tables 2–4) based on questionnaires sent to health personnel involved with snakebite. The questionnaires collected information on the vehicles used by the population in SBE emergencies on five main types of roads, their relative importance, and their estimated speed, both during the dry and wet seasons throughout the Terai. The detailed travel speed UI determination are given in Appendix 1 (p 2).

In our analysis, we included the two main seasons (dry and wet), which not only represent opposite peaks of snakebite incidence,^31^ but also how drivable the roads are. Additionally, we considered two snakebite syndromes (neurotoxic and haemotoxic), and the facilities treating them. Finally, we incorporated three transport modes: motorcycle, tempo-bike (also auto-rickshaw or tuk–tuk), and four-wheeled vehicles, each with three speeds (average, and upper and lower UI). These transport methods were the most common according to the responses from expert health personnel to our questionnaires and the baseline survey described in Alcoba and colleagues.^27^ The combination of these factors resulted in 36 individual travel time rasters (see data sharing). These continuous rasters were reclassified according to the syndrome they represented into three categories: neurotoxic: 1 for travel times between 0 and 30 min, 2 for 30–60 min, and 3 for >60 min; and haemotoxic: 1 for travel times between 0 and 3 h, 2 for 3–6 h, and 3 for >6 h. This reclassification represents mild, moderate, and severe risk of mortality or morbidity caused by travel time-related treatment delays. In Appendix 1 (Fig. 4), we compare the areas covered in each of the three travel time categories for the neurotoxic syndrome, considering the wet season, and motorcycle as transport method, and including the average speed, and the upper and lower speed UI for this vehicle. Additionally, for travel time analyses, an extension area of 10 km was added to all layers to the inland, northern part of the selected districts to account for a distance that people living near the Terai's immediate borders could prefer to travel to reach a facility (Appendix 1 Fig. 4).

### Vulnerability

We calculated a vulnerability index for SBE in the Terai by combining the risk of snakebite with the risk represented by severe outcomes (morbidity and/or mortality) due to deficient timely access to adequate medical treatment. We therefore multiplied the previous 36 reclassified travel-time rasters by three snakebite risk rasters (mean and mean ± SD). We kept 36 of the 108 resulting rasters by selecting the scenarios that represented the medium and the extreme combinations (high and low) of snakebite risk and travel time risk for each combination of season, transport method, and syndrome. In that framework, medium vulnerability represents the combination of average snakebite and travel time risks. High vulnerability combines the upper UI of snakebite and travel time risks, and low vulnerability represents the lower UI of snakebite and travel time risks (details in Appendix 1 p 2).

**Fig. 2:**
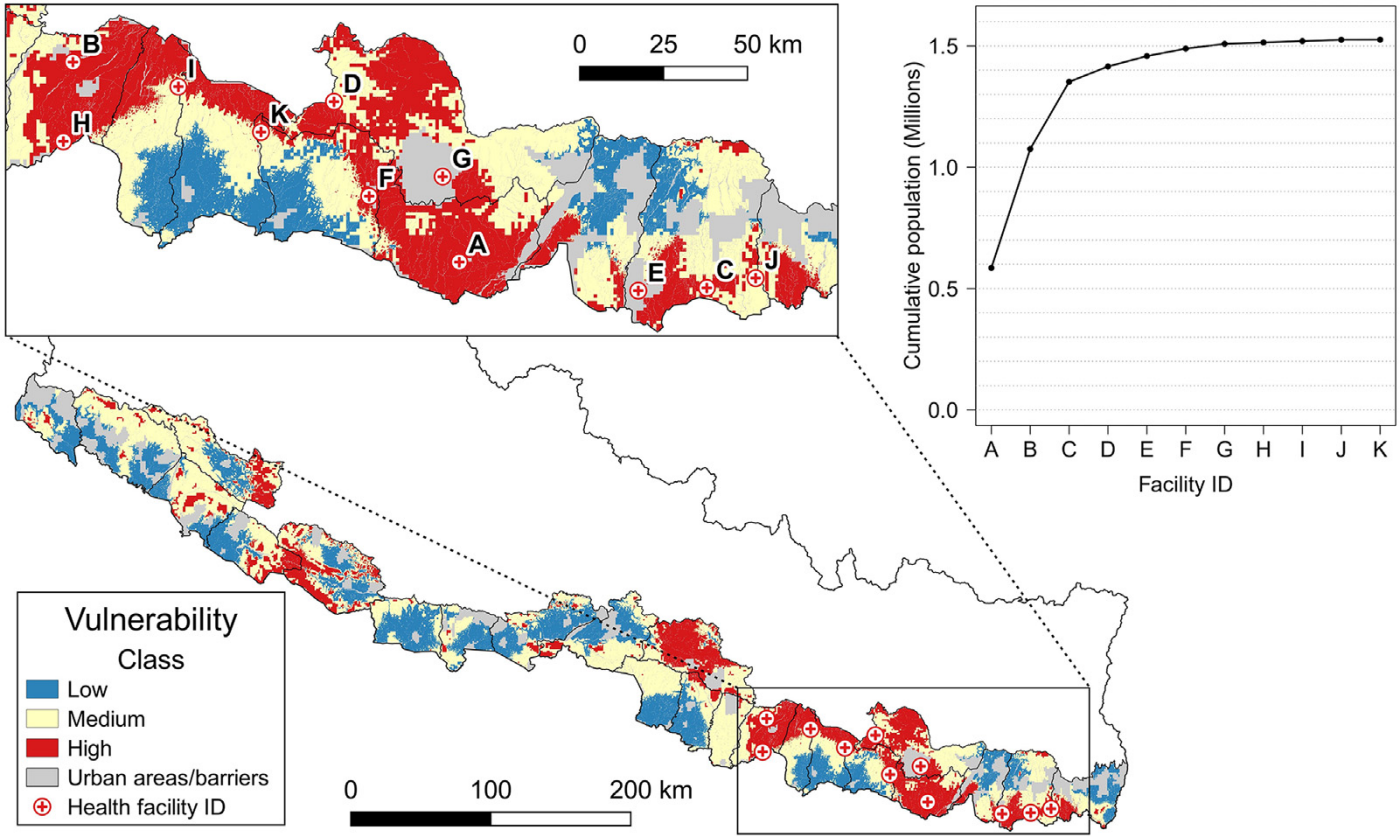
**3-Class vulnerability map for the standard scenario (neurotoxic syndrome, motorcycle, and wet season)**. The letters identify possible facilities to upgrade from treating only haemotoxic to also treat neurotoxic. **Internal plot:** Coverage of the cumulative population in high vulnerability areas within 60-min travel time. **Source:** vector map and administrative divisions from gadm.org, projected in the local WGS 84/UTM zone 45 N coordinate reference system in QGIS 3.24 (qgis.org).

For a clearer visualization and quantification of the vulnerable population in different areas, we further reclassified the risk of snakebite into three main classes based on the risk thresholds previously used in Ochoa and colleagues^6^: 1.5 for a risk between 0 and 0.01, 2.5 for 0.01–0.05, and 3.5 for >0.05. The 0.01 threshold value closely represents the mode of this distribution, which we chose as a transition from mild to moderate risk. The 0.05 threshold value is above 98.97% of the risk values and represents the end of the main distribution and the beginning of a long right tail expressing extreme values (severe risk). The values assigned to these classes allowed identification of each combination of snakebite and travel time risks in a 9-class vulnerability raster (Fig. 1d and e), and the population in each class (Appendix 1 Table 6). The 36 reclassified rasters can also be found in data sharing. For simplicity, the 9-class vulnerability rasters were aggregated into 3-class vulnerability rasters according to the code in Appendix 1 Fig. 2a.

### Healthcare scale-up analysis

**Fig. 3:**
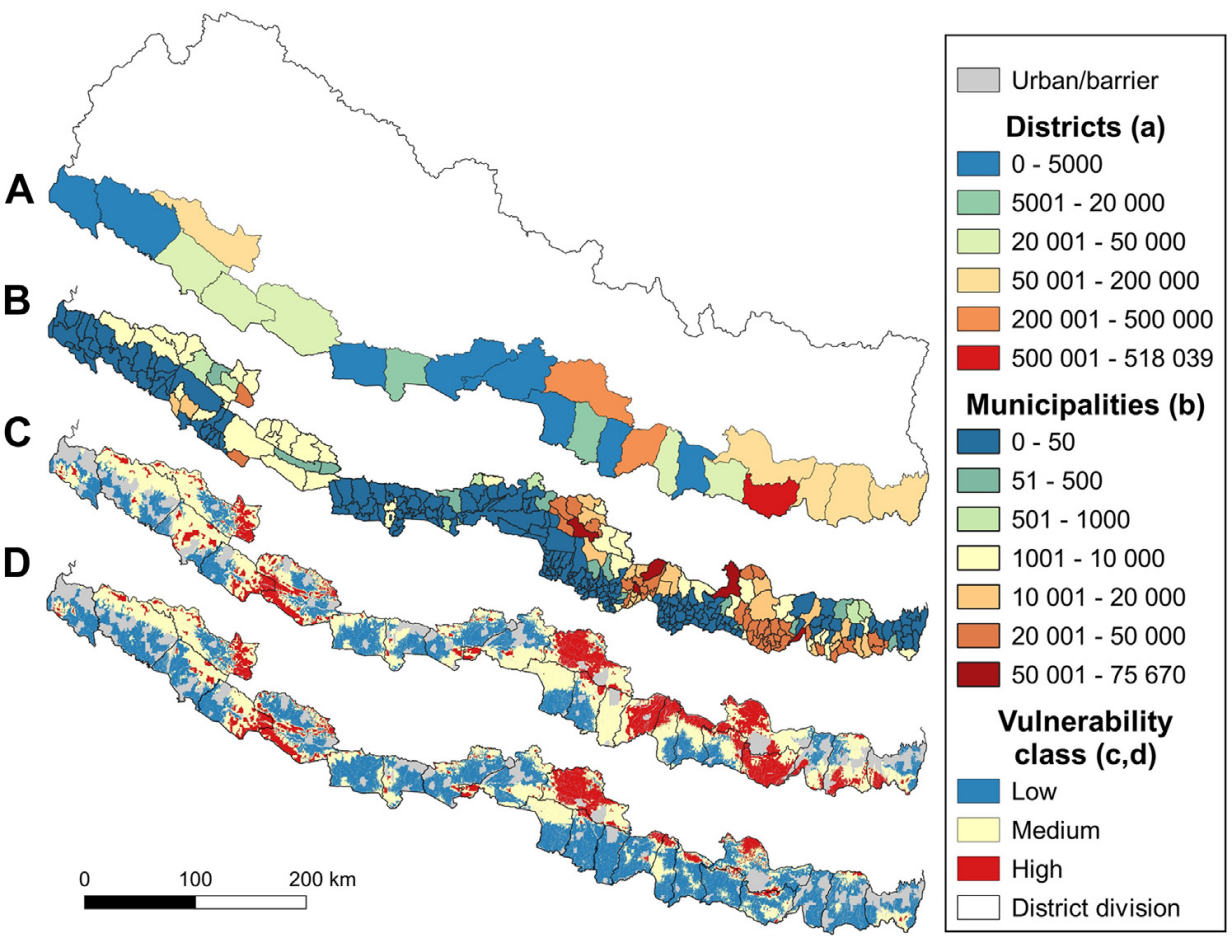
**Comparative maps of rural population and areas in the medium vulnerability scenario combining motorcycle, wet season, and neurotoxic syndrome**. Choropleth maps of the rural population per district **(a)** and municipality **(b)** falling into the high vulnerability class areas in red **(c)**. Reduced vulnerability through upgrade of all facilities without respirators so they can treat both syndromes **(d)**. **Source:** vector map and administrative divisions from gadm.org, projected in the local WGS 84/UTM zone 45 N coordinate reference system in QGIS 3.24 (qgis.org).

To illustrate the possibilities of scaling-up the access to healthcare in the Terai, we combined a 3-class map of population vulnerability, the geographical distribution of the rural population, and the location of health facilities treating haemotoxic SBE (no respirators) or no SBE at all, but that are willing to treat it. For the vulnerability map, we combined the average snakebite risk, the average speed of motorcycles (the most common vehicle),^11^^,^^32^ the wet season (when snakebite is more frequent),^10^^,^^26^^,^^31^ and the neurotoxic syndrome (the most widespread).^9^^,^^10^ We selected an area where facilities treating only haemotoxic syndrome were located in or near high-vulnerability populations, in regions of high rural population density, and whose catchments did not overlap with the catchment areas of current facilities treating neurotoxic syndrome. This resulted in 11 facilities in eastern Terai (Fig. 2). We quantified the population within each catchment area by overlapping their 60-min travel-time catchment areas with high vulnerability areas. The facilities ranked in descending order according to the population coverage in non-overlapping catchments are presented in Appendix 1, Table 8. Then, these values were plotted as a cumulative curve of the rural population classed at high vulnerability in each catchment area, starting by the largest catchment, followed in descending order by the next non-overlapping populations (Fig. 2). We also quantified the impact of potentially upgrading all facilities to treat both syndromes, again using wet season and the average speed of motorcycle as standard factors. To do this, we reprocessed the 3-class vulnerability map to account for all SBE treating facilities (Fig. 3d) and recalculated the population within 60 min travel time (Table 2).

**Table 1:** Rural population vulnerability.

Travel time scenario	Vulnerability class		
	Low n (%)	Medium n (%)	High n (%)
FWH	13,312,141 (98.30)	229,304 (1.69)	661 (0)
FWN	7,064,908 (52.17)	4,549,852 (33.60)	1,927,345 (14.23)
MWH	13,336,412 (98.48)	205,179 (1.52)	534 (0)
MWN	6,931,985 (51.19)	4,538,768 (33.52)	2,071,372 (15.30)

Example of rural population of the Terai in the medium vulnerability scenario and in the three vulnerability classes for four-wheeled vehicles (F) and motorcycle (M), wet season (W), and the haemotoxic (H) and neurotoxic (N) syndromes.

**Table 2:** Comparison of the population coverage at 60 min travel time between facilities treating neurotoxic syndrome and all available facilities, should they all be able to treat both syndromes.

District	Total population	Facilities treating neurotoxic syndromes		All snakebite treating facilities	
		Pop. at 60 min.	%	Pop. at 60 min.	%
Dhanusa	640,337	780,053	98.43	784,865	99.03
Mahottari	782,105	589,105	92.00	626,463	97.83
Sarlahi	651,541	30,763	**3.93**	768,619	**98.28**
Bara	396,335	513,849	78.87	643,359	98.74
Chitawan	323,808	314,332	79.31	346,314	87.38
Makwanpur	1,009,486	51,344	15.86	56,561	17.47
Parsa	692,208	958,404	94.94	985,353	97.61
Rautahat	578,526	172	**0.02**	687,741	**99.35**
Morang	955,134	151,786	**26.24**	556,607	**96.21**
Sunsari	453,543	460,288	**48.19**	945,375	**98.98**
Jhapa	634,286	366,451	80.80	447,950	98.77
Saptari	816,564	22,946	**3.62**	628,903	**99.15**
Siraha	315,959	763,244	93.47	808,838	99.05
Udayapur	606,252	40,820	**12.92**	251,939	**79.74**
Kanchanpur	689,847	529,387	87.32	599,002	98.80
Kailali	388,303	582,267	84.41	635,059	92.06
Banke	338,668	332,839	85.72	334,008	86.02
Bardiya	237,389	173,078	51.11	315,977	93.30
Surkhet	478,701	97,380	41.02	97,380	41.02
Dang	663,344	388,232	81.10	444,881	92.93
Kapilbastu	548,009	647,543	97.62	657,338	99.09
Nawalparasi	800,698	504,511	92.06	512,125	93.45
Rupandehi	792,525	795,678	99.37	796,013	99.41
Total	**13,793,567**	**9,094,472**	**65.93**	**12,930,671**	**93.74**

Highlighted in bold are high priority districts with a neurotoxic syndrome coverage improvement greater than 50%.

### Role of the funding source

The study sponsor had no role in study design, data collection, analysis, or interpretation, manuscript writing, or the decision to submit for publication.

## Results

In the Terai, we identified 96 SBE treating facilities, including 56 without respirators (haemotoxic treatment only), three equipped only with respirators (neurotoxic treatment only), and 37 with SAV and respirators (treating both syndromes). We present general results for all 36 scenarios, and specific results for the most relevant, which includes neurotoxic syndrome, wet season, and motorcycle (average speed) as main transport method. We started by calculating the catchment area and population coverage of all facilities according to the travel time thresholds for each syndrome. In terms of coverage, there was a clear difference between the neurotoxic and haemotoxic syndromes (Table 1, Fig. 1b and c). Continuous travel time maps comparing neurotoxic and haemotoxic syndromes for the wet season and motorcycle average speed can be found in Appendix 1, Fig. 3. All travel time raster files can be found in data sharing.

For the haemotoxic syndrome, the larger number of well-distributed facilities combined with the much longer travel time threshold meant that all districts have a current coverage of their population above 97% within the 180-min travel time threshold representing mild symptoms (Appendix 1 Table 5). The vulnerability analysis showed that for all scenarios considering the haemotoxic syndrome, most of the rural population (between 92.83 and 99.98%) fell into a low vulnerability class, mainly due to reduced travel time risk (Fig. 1c and Appendix 1 Table 7). In contrast, only 0.33% of the population (in the very worst scenario) fell into the high vulnerability class for this syndrome. Instead, the neurotoxic syndrome had a much larger proportion of the population falling into the high or medium vulnerability classes (Appendix 1 Table 7). In line with these results, we focused our analysis on the neurotoxic syndrome, which shows greater deficiencies and inequalities in terms of coverage across the Terai.

For the most plausible neurotoxic scenario described above, six districts in the eastern and central Terai had population coverage of less than 30% (Appendix 1 Table 5, bold font) within the 60-min travel time threshold, indicating moderate morbidity risk. In three districts, extreme values showed that more than 95% of the population faced a travel time of more than 60 min to reach a facility that can treat the neurotoxic syndrome, placing them in the severe risk category. Considering the 18 neurotoxic scenarios, for the most optimistic, which includes motorcycle's upper-speed UI during the dry season, coverage values showed a minimum of 28.65% in the Sarlahi district (Appendix 2), yet leaving four districts with less than 50% of their population outside the 60-min coverage threshold. In contrast, under the most pessimistic scenario, which takes into account tempo-bike's lower-speed UI during the wet season, only five of the 23 districts considered for the Terai have a population coverage above 50%. When analysing vulnerability for the same optimistic scenario in the whole Terai, just over 300 000 people (2.29%) fell into the high vulnerability class, while 11.3 million (83.75%) fell into the low vulnerability class (Appendix 1 Table 7). For the most pessimistic scenario, on the other hand, more than 6.8 million rural people in all the Terai (50.43%) fell into the high vulnerability class and only 2.47 million (18.2%) into the low vulnerability class.

The overall results for tempo-bike scenarios were worse than those for motorcycle and four-wheeled vehicles (4WV), comparatively including more population in the high vulnerability class and fewer in the low vulnerability class (Appendix 1 Fig. 1). This is likely due to the lower speed of these vehicles and limited accessibility to side roads and paths. In contrast, motorcycles and 4WV are faster and can therefore cover larger areas in the 30- and 60-min travel times agreed for the neurotoxic syndrome. The results for motorcycles and 4WV were very similar in all three vulnerability classes, as seen in Table 1 (for all scenarios, see Appendix 1, Table 7). Although 4WVs are slightly faster on average on most roads (Appendix 1 Tables 2 and 4), the possibility of riding motorcycles on all types and sizes of roads ultimately meant larger coverage areas, even if additional population was not always covered.

Selecting a low, medium, or high vulnerability scenario strongly affected the number of people falling into each vulnerability class. A clear example can be seen in Appendix 1 Fig. 1, bottom panel for low vulnerability class, where for all combinations, the low vulnerability scenarios (represented by the downward-pointing triangles) always included more people. Meanwhile, the people falling into the different vulnerability classes for the medium vulnerability scenario depended more on seasonality and transport method (Appendix 1 Fig. 1). To better understand the individual effect of the factors considered, we compared the percentages of the rural population falling into the high vulnerability class for all neurotoxic syndrome scenarios, while keeping additional factors stable. The comparison between seasons shows that on average 36.4% (95% CI 19.76–53.1) more people are expected to be in the high vulnerability class due to wet season conditions. Regarding the different vehicles analysed, using the tempo-bike as transport mode instead of a motorcycle represents a 34.69% (95% CI 16–53.38) increase in the population in the high vulnerability class. As mentioned above, the effects on vulnerability of using motorcycles instead of 4WVs is minimal: on average 1.62% (95% CI -2.0–5.24) more people fall into the high vulnerability class.

Our scale-up analysis shows the population coverage in the high vulnerability class for each of the selected facilities, while excluding the overlapping populations between their catchments (Appendix 1 Table 8). The Gajendra Narayan Zonal Hospital (A) would see the greatest improvement. By enhancing mechanical respirators’ availability and training personnel to operate them, this facility alone would cover and reduce the high neurotoxic vulnerability of more than half a million people in adjacent rural areas. Additionally, upgrading the Nawalpur Snakebite Treatment Centre (B) and the Rangeli District Hospital (C) could provide, in total, access to neurotoxic treatment to more than 1.3 million people within shorter travel times (less than 60 min). This shows that upgrading a few selected facilities could significantly improve the overall deficit in treatment coverage to the rural population in the Terai. This upgrade could serve the district and regional health authorities as a prospective Terai-wide upgrading. To evaluate this idea, we visually compared the 3-class distribution of vulnerability in the Terai for our standard neurotoxic scenario including wet season and transport by motorcycle at average speed, and the potential scenario where all snakebite facilities treat both syndromes (Fig. 3d). The largest and more evident reduction in the high vulnerability class was found in the eastern Terai, which confirmed the selection of facilities done for the upgrade of three facilities. If all snakebite treating facilities that currently cannot optimally treat neurotoxic syndrome were upgraded, about 3.8 million people in high vulnerability areas for that syndrome in the rural Terai could be covered within the 60-min severity threshold (moderate symptoms), and increase from 65.93 to 93.74% (Table 2). This would also reduce the high-vulnerability rural population from 2.07 to 0.4 million. The districts of Sarlahi, Rautahat, and Saptari, where coverage could increase by more than 90% and where the highest snakebite incidence in the Terai was recently reported,^2^ would particularly benefit. Additionally, six districts could profit from a coverage increase of more than 50% (Table 2 highlights). Despite this potential coverage improvement (Fig. 3d), the districts of Makwanpur (17.47%) and Surkhet (41.02%) would still have deficient population coverages, which might only be improved by adding SBE treating capabilities (SAV, respirators, and staff) to well-placed facilities not treating SBE so far.

Fig. 3 shows choropleth maps per district (a) and municipality (b) again for the medium vulnerability scenario combining motorcycle, wet season, and neurotoxic syndrome, and summarising the estimated population in the high vulnerability class. This highlights the heterogeneity in vulnerability inside the districts, translates into more realistic population numbers, and emphasizes the importance of fine resolution rasters (c) to identify in detail the areas and populations with greater health coverage deficiencies. Zoomable, interactive versions of (b) as well as the equivalent maps for low and high vulnerability scenarios can be found in appendices 4–6.

## Discussion

We conducted a high-resolution geospatial analysis of the vulnerability to snakebite of the rural population in the Terai region of Nepal, combining snakebite risk with different scenarios of travel time and accessibility to healthcare, and for the first time including empiric UI for both of them. Our results have shown that the neurotoxic syndrome is a key determinant of vulnerability to snakebite, as the threshold for severity is much lower, which has a particularly strong impact in the eastern Terai. Targeted capacity building of facilities would significantly increase population treatment coverage and reduce vulnerability.

Modelling accessibility to specific healthcare is particularly helpful to recognize how well a population is covered, to detect its vulnerabilities, and to optimize allocation of resources.^5^ Our study focuses on the second of the three-delay framework,^33^ which considers the time between deciding to seek help and reaching the health facility. This can often be the longest delay because it is influenced by external factors such as distance, type of transport, landscape, climate, etc. Unfortunately, in the Terai, delays both in deciding to seek medical help (first delay), due to the preference for visiting traditional healers,^11^^,^^13^ and in dispensing the actual treatment at health centres (third delay), due to the lack of trained personnel and/or equipment,^9^^,^^10^ are also common. Still, the second delay seems to offer the best opportunity for bite-to-treatment time reduction, firstly by understanding the impact of different scenarios and secondly by analysing the accessibility and availability of health facilities with SAV, respirators, and the medical staff trained to use them. It is therefore necessary to understand the dynamics of accessibility to healthcare for the two SBE syndromes, but especially for the neurotoxic one, as it poses a greater immediate health threat and is the predominant syndrome in the Terai. It is equally important to understand at the highest spatial resolution possible how different factors affect vulnerability to SBE, in order to propose solutions that are adapted to the specific conditions in the concerned areas and contribute to more equitable health coverage for rural populations.

SBE is a complex, multifactorial phenomenon^3^^,^^6^ and this complexity is expressed and amplified when more intricate traits, such as vulnerability to SBE, are considered. In our analysis, in addition to the already multidimensional snakebite risk, we determine vulnerability to SBE by also including multiple travel time scenarios. These scenarios are influenced by the geospatial characteristics of the Terai, the modes of transport used by the population, the two main seasons, and the severity of the envenoming syndromes present. They also represent unique combinations of snakebite and travel-time risk parameters that affect exposed areas differently. The overlap of these areas with the distribution of the rural population defines the SBE vulnerability to which the population is exposed.

For the haemotoxic syndrome, the longer agreed travel time thresholds for mild and moderate envenoming, the rather flat and narrow geography of the Terai region, and the large number of health facilities treating it meant that most of the rural population (up to 99.98% according to scenario) should fall into low vulnerability areas. In contrast, for the neurotoxic syndrome, where the time thresholds for mild and moderate envenoming are much shorter and there are fewer facilities fully equipped to treat it, much larger proportions of the rural population are at high vulnerability (up to 50.43% depending on the scenario). This is particularly problematic, considering that an estimated 74.4% (32/43) of severe envenoming cases in the Terai are neurotoxic.^2^

Our expert-based travel-speed results and our baseline survey^27^ confirmed that motorcycle is an extremely frequent transport method for snakebite victims in the Terai region. This validates the practicality and relevance of this vehicle as victim transport from previous studies in eastern Terai and the neighbouring Bihar in India.^11^^,^^32^ The results for 4WV are very similar, having slightly less coverage than motorcycles in most scenarios, likely due to limited access into smaller roads and paths. Despite the practicality and accessibility of motorcycles, ambulances and other 4WVs provide an additional level of protection for snakebite victims, especially during the most intense period of the wet season, so they could be preferable when available and affordable. The use of specific transport modes and even the privation of one could be determined by the affordability of the associated costs for each of them, adding an extra layer of inequity to an already neglected section of society.

Regarding the possible scale-up of health facilities, our local evaluation in the Terai revealed that very few of them are willing to include snakebite treatment in their services. We therefore concluded that the simplest and most cost-effective solution would be to upgrade some of the more numerous and widely distributed facilities without mechanical respirators to also fully treat neurotoxic cases. Our local scale-up results have shown that even the careful selection of one or two facilities in highly vulnerable areas could represent a substantial improvement towards integral rural population coverage of SBE healthcare. The focus on the eastern Terai is also confirmed by a recent Terai-wide cross-sectional survey analysis, where 65.6% (21/32) of neurotoxic SBE occurred in this region.^2^ In an ideal scenario, where all facilities currently treating SBE could optimally treat both syndromes, the coverage of the rural population would be above 93%, with about 863 000 people remaining uncovered in some districts. This could be addressed by developing SBE treating capabilities in strategically placed health centres not currently treating SBE. Additionally, a better understanding of the distribution of syndromes could also help prioritizing health facilities for upgrading or development.

Our use of UI in travel speed and snakebite risk to calculate vulnerability resulted in extreme values that both indicate the need for further research, as more accurate vulnerability estimates with lower uncertainty should result from more detailed data. Future improvements could include more exhaustive and computationally demanding methods to estimate uncertainty in a more dynamic and realistic way eg, by generating 95% UI from replicated random processes. Fundamental elements of our analysis are the travel scenarios and their speed estimations questionnaires. These data rely on the respondent's personal assessment of speed and are, therefore, already subject to some uncertainty. This could be improved eg, by including additional speed estimation methods, such as GPS tracking of specific types of vehicles, which adds logistic difficulties to be considered. The presented high vulnerability scenarios show plausible extreme values that could indicate priority areas for improving neurotoxic treatment capabilities, and as a guide for the SAV stockpiling scheme currently in development by WHO in its roadmap for snakebite.

Clearly, the availability of mechanical respirators and medical personnel who can use them correctly is a main limiting factors for good coverage of neurotoxic SBE in the Terai. One solution, once the Covid-19 pandemic halts in Nepal, would be to repurpose the more than 300 respirators donated by the international community when the delta variant of SARS-CoV-2 hit Nepal in April 2021. Enhancing capacity by training personal to operate these equipment would be a way forward.

By including multiple factors like climate, syndrome, and transport method into travel time rasters and combining these with snakebite risk, we were able to determine vulnerable populations under a variety of conditions.^5^ However, a clear limitation to this type of modelling is the availability and accessibility of up-to-date and reliable data on health facilities and their capabilities to treat snakebite (SAV, mechanical respirators, and personnel). As SBE occurs mainly in LMIC, having well-documented health systems that include georeferenced facilities, is uncommon. The lack and reduced access to this type of data in Nepal, in part due to its recent transition to federalism, compelled us to gather this information through open data and direct phone calls and visits.

With this analysis, we highlight vulnerability hotspots based on the risk of snakebite and of delayed treatment due to travel time. These hotspots are mainly evident in the case of neurotoxic syndrome, as it becomes severe within shorter (travel) time. To address this problem, new snakebite treatment centres could be established in these hotspots. However, in resource-constrained settings, a more realistic option might be to upgrade facilities that already treat SBE but do not have respirators or even trained staff. Ideally, any centre treating SBE should be able to deal with any syndrome without the need for referral, especially for the neurotoxic syndrome where a few minutes can make the difference between mild and severe outcomes. For this study, we assumed that only SBE treating centres with operating mechanical respirators would be able to optimally treat neurotoxic envenoming. However, centres with SAV, manually assisted ventilation (eg, bag-valve mask ventilation), and an ambulance referral system to hospitals with mechanical ventilators can also save lives, even if their contribution to partially reducing the vulnerability may be more difficult to quantify. It is imperative to emphasise that in all cases of snakebite, transport should be started as soon as possible after the bite, without waiting for any symptoms to appear. Reducing population vulnerability via the improvement of accessibility to snakebite healthcare services will undoubtedly translate into a reduction in the number of deaths and disabilities caused by this neglected tropical disease.

## Contributors

CO and NR designed the spatial sampling methodology and conceptualized the analysis. CO analysed and interpreted the data, produced the output figures, and wrote the initial manuscript. CO, NR, FC, SBM, RRdC, IB, GA, and SKS conceived and designed the study. FC and NR oversaw the SNAKE-BYTE project and raised funding. MR coordinated data collection in health facilities and prepared secondary data in the Terai. All authors contributed to subsequent revisions and approved the final version submitted for publication. NR, FC and CO had final responsibility for the decision to submit the study for publication.

## Data sharing statement

The data used and the sources are described in this article and in the supplementary materials. The vulnerability rasters can be found in https://doi.org/10.26037/yareta:2l67hobrufaarox3wmvg564dka and the original snakebite predicted risk raster are accessible in https://doi.org/10.26037/yareta:qqyq2a7mo5bdrgp4qolvc4q6cq.

## Editor note

The Lancet Group takes a neutral position with respect to territorial claims in published maps and institutional affiliations.

## Declaration of interests

The project was funded by The 10.13039/501100001711Swiss National Science Foundation (10.13039/501100001711SNSF) (SNAKE-BYTE - project number 315130_176271) and the recipients of grant are Nicolas Ray and François Chappuis. The authors declare no competing interests.

## Appendix A Supplementary data

Appendix 6

Caption for Supplementary Material
