# Supplementary material for: Vulnerability to snakebite envenoming and access to healthcare in the Terai region of Nepal: a geospatial analysis
Source: Lancet Reg Health Southeast Asia. 2022 Nov 17;9:100103. doi: 10.1016/j.lansea.2022.100103 (PMC10306013; doi:10.1016/j.lansea.2022.100103)
Supplement: Appendix 1 [file mmc1.docx]

**Supplementary material**

**Vulnerability to snakebite envenoming and access to healthcare in the Terai region of Nepal: a geospatial analysis**

Carlos Ochoa^1,2*^, PhD

Mamit Rai^3^, BSocSc

Sara Babo Martins^1^, PhD

Gabriel Alcoba^1,4,5^, MD

Isabelle Bolon^1^, PhD

Rafael Ruiz de Castañeda^1^, PhD

Sanjib Kumar Sharma^3^, MD

François Chappuis^5,6^, MD

Nicolas Ray^1,2^, PhD

^1^ Institute of Global Health (IGH), Department of Community Health and Medicine, Faculty of Medicine, University of Geneva, Geneva, Switzerland

^2^ Institute for Environmental Sciences (ISE), University of Geneva, Geneva, Switzerland

^3^ KHDC-Nepal, Dharan, Nepal

^4^ Médecins Sans Frontières (MSF), Geneva, Switzerland

^5^ Division of Tropical and Humanitarian Medicine, Geneva University Hospitals (HUG), Geneva, Switzerland

^6^ B.P. Koirala Institute of Health Sciences (BPKIHS), Dharan, Nepal

^7^ Department of Community Health and Medicine, Faculty of Medicine, University of Geneva, Geneva, Switzerland

*Corresponding author:

**Carlos Ochoa**

[carlos.ochoa@unige.ch](mailto:carlos.ochoa@unige.ch)

Institute of Global Health

Faculty of Medicine, University of Geneva

Chemin des Mines 9

1202 Geneva, Switzerland

+41 22 379 07 62

**Methodological details**

**Geospatial data preparation**

All layers were projected into the WGS 84/UTM zone 45 N local coordinate reference system and all rasters were aligned with each other. All manipulations and calculations of the spatial data layers were done using QGIS v.3.24.^1^ and R v.4.1.2.^2^ All raster layers were obtained at 30 metres resolution, except for the snakebite risk, which had an original resolution of 1 km and was resampled to match the other layers. The point-shape file for the hanging bridges, which mainly contains the location, length, and structural condition of thousands of suspension trail bridges built over the last 50 years, was made available on request. The point locations for the trail bridges were converted into polygons covering the entire length of the bridge each represents connecting the two opposite ends of the path.

**General description of the snakebite-risk map construction**

The risk map is the result of the predictive analysis of a logistic hierarchical Bayesian model using the Integrated Nested Laplace Approximation (INLA) methodology. The values represent the posterior distributions estimated at 39 684 points at 1 km intervals throughout the Terai, excluding by design densely populated areas (Village Development Committees) with >20 000 inhabitants where snakebites are considered uncommon.^3^ The statistical methods, covariates, validation, and other results are discussed in detail in Ochoa and collaborators.^4^

**Medically Important Venomous Snakes and the snakebite risk**

For the snakebite risk map used here, Ochoa and collaborators used the medically important venomous snake (MIVS) definitions (primary and secondary) from WHO and the distributions developed by Roll and collaborators.^5^ According to this study, there are up to 10 MIVS at any location in the Terai. That study was based on a survey that could not identify reliably the biting species of snake. So instead of analysing individual species, we analysed species richness, which was ultimately not a significant factor.

**Classification of facilities by syndrome**

The classification of facilities into those that can treat neurotoxic or haemotoxic syndromes was crucial for the analysis of accessibility. This classification aimed to define the facilities able to provide appropriate clinical management for each of the two syndromes, including any degree of severity, but especially in cases of delayed presentation and victims with advanced signs of envenoming. As part of this aim, facilities with both mechanical respirators and SAV were considered independently for the travel time analysis of each syndrome, in addition to the facilities that could only treat adequately one syndrome. These definitions must be kept general because, from an expert clinical perspective, the use of SAV may be more or less useful depending eg, on the type of snake (krait, cobra, or viper), and the degree of envenomation, which greatly depends on the delay between bite and treatment.

**Uncertainty determination**

The uncertainty for each of the main roads was calculated as average ±SD. The speed uncertainty, for roads without one, was ±19·5% of the agreed speed value. This value was the overall average of the proportions between the average speed and SD for all roads, seasons, and vehicles in the travel speed scenarios. For smaller roads that were not included in our travel speed surveys, we took mean speed values from those roads in the Terai in OpenStreetMaps. For off-road speeds, we used the values described in a detailed accessibility analysis in Nepal, which also included a 25% speed reduction in the wet season.^6^

**Vulnerability scenarios and catchment areas**

The high and low vulnerability values include in their calculation the upper or lower uncertainty intervals (UI) of snakebite risk, respectively. Additionally, they include the less direct travel time risk. For the high vulnerability values, the high travel time risk is the lower speed UI for each transport method, which results in smaller catchment areas within the mild and moderate time ranges for each syndrome (figure 2b). At the same time, this makes the areas of severe risk, outside the catchment (>60 minutes) proportionally larger. For the low vulnerability, the low travel time risk represents the upper speed UI for each transport method, resulting in bigger areas within the mild and moderate time ranges for each syndrome (figure 2b). Similarly, this makes the areas of severe risk, outside the catchment (>60 minutes) proportionally smaller.

The final 36 vulnerability rasters were normalised to a 0–1 range (accessible from the main-file data sharing link and appendix 3). It is worth noting that some values of the lower limit for snakebite risk (mean–SD) were borderline negative, thus to avoid negative vulnerability, these negative risk values were zero-truncated. Additionally, although for some scenarios we combined values of two independent UI, one for snakebite risk and one for travel speeds, the combination of the two cannot be considered a conventional measure of dispersion from the mean. Instead, it represents upper and lower extreme values of the main combination of factors used to define vulnerability (see table 7 & figure 1).

**Table 1. Data layers and sources.**

| Layer | Source | Type | Initial resolution |
| --- | --- | --- | --- |
| Digital elevation model (DEM) | Shuttle Radar Topography Mission (SRTM GL1)^7^ | Raster | 30 m |
| Population (Nepal, 2018) | Facebook connectivity lab and CIESIN^8^ | Raster | 30 m |
| Waterlines and waterbodies | [GeoFabrik](http://download.geofabrik.de/asia/nepal-latest-free.shp.zip) and OSM^9^ | Vector/polygon |  |
| Landcover | ESA WorldCover consortium^10^ | Raster | 30 m |
| Roads | [GeoFabrik](http://download.geofabrik.de/asia/nepal-latest-free.shp.zip) and OSM^9^ | Vector |  |
|  | [Facebook microsites AI predicted roads](https://github.com/facebookmicrosites/Open-Mapping-At-Facebook/wiki/Available-Countries) | Vector |  |
| Trail bridges | Helvetas Swiss Inter-Cooperation^11^ | Points |  |
| Predicted snakebite risk | Ochoa et al.^4^ | Raster | 1 km |
| Health facilities | [Global Healthsites Mapping Project](https://data.humdata.org/dataset/nepal-healthsites) | Points |  |
|  | [Survey Department of Nepal](https://data.humdata.org/dataset/nepal-health-facilities-cod) | Points |  |
|  | Nepali Army snakebite centres (local experts) | Points |  |
|  | OpenStreetMaps^9^ through [HealthSites.io](https://healthsites.io/) project | Points |  |

**Table 2. AccessMod travel scenarios for motorcycle**. Summary of the input scenario table used to define the travel speed and method for each combination of speed, landscape, climate, and road. The 'Class' column contains individual identifiers assigned to each type of road or landcover. The 'Label' column contains the main descriptive name for each type of road or landcover. The columns under 'Speed' represent the estimated speed for the specified UI and climatic conditions. The 'Mode' column is needed in AccessMod to assign to each type of road or landcover the main transport mode, which can be on foot (WALKING), cycling (BYCYCLING), or by using motorized vehicles (MOTORIZED). For further details see: https://doc-accessmod.unepgrid.ch/display/EN/

| Class | Label | Speed (km/h) | | | | | | Mode |
| --- | --- | --- | --- | --- | --- | --- | --- | --- |
|  |  | Average  Dry | Average  Wet | Upper  Dry | Upper  Wet | Lower  Dry | Lower  Wet |  |
| 10 | Tree cover | 3·2 | 2·4 | 3·8 | 2·9 | 2·6 | 1·9 | WALKING |
| 20 | Shrubland | 3·6 | 2·7 | 4·3 | 3·2 | 2·9 | 2·2 | WALKING |
| 30 | Grassland | 4·8 | 3·6 | 5·7 | 4·3 | 3·9 | 2·9 | WALKING |
| 40 | Cropland | 3·2 | 2·7 | 3·8 | 3·2 | 2·6 | 2·2 | WALKING |
| 50 | Built-up | 5 | 3·7 | 6·0 | 4·4 | 4·0 | 3·0 | WALKING |
| 60 | Bare sparse vegetation | 3 | 2·2 | 3·6 | 2·6 | 2·4 | 1·8 | WALKING |
| 70 | Snow and Ice | 1·6 | 1·2 | 1·9 | 1·4 | 1·3 | 1·0 | WALKING |
| 80 | Permanent water bodies | 0 | 0 | 0·0 | 0·0 | 0·0 | 0·0 | WALKING |
| 90 | Herbaceous wetland | 0·5 | 0 | 0·6 | 0·0 | 0·4 | 0·0 | WALKING |
| 1001 | trunk | 55 | 45 | 64·4 | 50·0 | 45·7 | 40·0 | MOTORIZED |
| 1002 | trunk link | 55 | 45 | 64·4 | 50·0 | 45·7 | 40·0 | MOTORIZED |
| 1003 | primary | 55 | 45 | 64·4 | 50·0 | 45·7 | 40·0 | MOTORIZED |
| 1005 | secondary | 48·8 | 40 | 56·2 | 43·5 | 41·4 | 36·5 | MOTORIZED |
| 1006 | secondary link | 48·8 | 40 | 56·2 | 43·5 | 41·4 | 36·5 | MOTORIZED |
| 1007 | tertiary | 42·5 | 33·8 | 48·1 | 40·3 | 36·9 | 27·3 | MOTORIZED |
| 1008 | tertiary link | 42·5 | 33·8 | 48·1 | 40·3 | 36·9 | 27·3 | MOTORIZED |
| 1009 | road | 20 | 15 | 23·9 | 17·9 | 16·1 | 12·1 | MOTORIZED |
| 1011 | residential | 33·8 | 27·5 | 40·3 | 35·0 | 27·3 | 20·0 | MOTORIZED |
| 1012 | living street | 20 | 15 | 23·9 | 17·9 | 16·1 | 12·1 | MOTORIZED |
| 1013 | service | 33·8 | 27·5 | 40·3 | 35·0 | 27·3 | 20·0 | MOTORIZED |
| 1014 | track | 33·8 | 27·5 | 40·3 | 35·0 | 27·3 | 20·0 | MOTORIZED |
| 1015 | unclassified | 33·8 | 27·5 | 40·3 | 35·0 | 27·3 | 20·0 | MOTORIZED |
| 1016 | bridleway | 20 | 6·2 | 23·9 | 7·4 | 16·1 | 5·0 | MOTORIZED |
| 1017 | pedestrian | 30 | 23·8 | 36·1 | 33·4 | 23·88 | 14·2 | MOTORIZED |
| 1018 | path | 30 | 23·8 | 36·1 | 33·4 | 23·88 | 14·2 | MOTORIZED |
| 1019 | footway | 30 | 23·8 | 36·1 | 33·4 | 23·88 | 14·2 | MOTORIZED |
| 1020 | cycleway | 20 | 6·2 | 23·9 | 7·4 | 16·1 | 5·0 | MOTORIZED |
| 1021 | steps | 1·5 | 1 | 1·8 | 1·2 | 1·2 | 0·8 | WALKING |
| 1022 | construction | 0 | 0 | 0·0 | 0·0 | 0·0 | 0·0 | WALKING |
| 1050 | hanging bridges | 3 | 2·4 | 3·6 | 2·9 | 2·4 | 1·9 | WALKING |
| 1051 | bridge | 30 | 23·8 | 36·1 | 33·4 | 23·88 | 14·2 | MOTORIZED |

**Table 3. AccessMod travel scenarios for Tempo (motorized tricycle).** Summary of the input scenario table used to define the travel speed and method for each combination of speed, landscape, climate, and road. The 'Class' column contains individual identifiers assigned to each type of road or landcover. The 'Label' column contains the main descriptive name for each type of road or landcover. The columns under 'Speed' represent the estimated speed for the specified UI and climatic conditions. The 'Mode' column is needed in AccessMod to assign to each type of road or landcover the main transport mode, which can be on foot (WALKING), cycling (BYCYCLING), or by using motorized vehicles (MOTORIZED). For further details see: https://doc-accessmod.unepgrid.ch/display/EN/

| Class | Label | Speed (km/h) | | | | | | Mode |
| --- | --- | --- | --- | --- | --- | --- | --- | --- |
|  |  | Average  Dry | Average  Wet | Upper  Dry | Upper  Wet | Lower  Dry | Lower  Wet |  |
| 10 | Tree cover | 3·2 | 2·4 | 3·8 | 2·9 | 2·6 | 1·9 | WALKING |
| 20 | Shrubland | 3·6 | 2·7 | 4·3 | 3·2 | 2·9 | 2·2 | WALKING |
| 30 | Grassland | 4·8 | 3·6 | 5·7 | 4·3 | 3·9 | 2·9 | WALKING |
| 40 | Cropland | 3·2 | 2·7 | 3·8 | 3·2 | 2·6 | 2·2 | WALKING |
| 50 | Built-up | 5 | 3·7 | 6·0 | 4·4 | 4·0 | 3·0 | WALKING |
| 60 | Bare sparse vegetation | 3 | 2·2 | 3·6 | 2·6 | 2·4 | 1·8 | WALKING |
| 70 | Snow and Ice | 1·6 | 1·2 | 1·9 | 1·4 | 1·3 | 1·0 | WALKING |
| 80 | Permanent water bodies | 0 | 0 | 0·0 | 0·0 | 0·0 | 0·0 | WALKING |
| 90 | Herbaceous wetland | 0·5 | 0 | 0·6 | 0·0 | 0·4 | 0·0 | WALKING |
| 1001 | trunk | 37·5 | 33 | 43·1 | 41·3 | 31·9 | 24·7 | MOTORIZED |
| 1002 | trunk link | 37·5 | 33 | 43·1 | 41·3 | 31·9 | 24·7 | MOTORIZED |
| 1003 | primary | 37·5 | 33 | 43·1 | 41·3 | 31·9 | 24·7 | MOTORIZED |
| 1005 | secondary | 35 | 29 | 38·5 | 36·4 | 31·5 | 21·6 | MOTORIZED |
| 1006 | secondary link | 35 | 29 | 38·5 | 36·4 | 31·5 | 21·6 | MOTORIZED |
| 1007 | tertiary | 30 | 24 | 39·4 | 34·0 | 20·7 | 14·0 | MOTORIZED |
| 1008 | tertiary link | 30 | 24 | 39·4 | 34·0 | 20·7 | 14·0 | MOTORIZED |
| 1009 | road | 20 | 15 | 23·9 | 17·9 | 16·1 | 12·1 | MOTORIZED |
| 1011 | residential | 30 | 24 | 36·1 | 31·4 | 23·9 | 16·6 | MOTORIZED |
| 1012 | living street | 20 | 15 | 23·9 | 17·9 | 16·1 | 12·1 | MOTORIZED |
| 1013 | service | 30 | 27·5 | 36·1 | 31·4 | 23·9 | 16·6 | MOTORIZED |
| 1014 | track | 30 | 27·5 | 36·1 | 31·4 | 23·9 | 16·6 | MOTORIZED |
| 1015 | unclassified | 30 | 27·5 | 36·1 | 31·4 | 23·9 | 16·6 | MOTORIZED |
| 1016 | bridleway | 4·3 | 3·7 | 4·8 | 4·2 | 3·8 | 3·2 | WALKING |
| 1017 | pedestrian | 4·3 | 3·7 | 4·8 | 4·8 | 3·8 | 3·0 | WALKING |
| 1018 | path | 4·3 | 3·7 | 4·8 | 4·8 | 3·8 | 3·0 | WALKING |
| 1019 | footway | 4·3 | 3·7 | 4·8 | 4·8 | 3·8 | 3·0 | WALKING |
| 1020 | cycleway | 4·3 | 3·7 | 4·8 | 4·2 | 3·8 | 3·2 | WALKING |
| 1021 | steps | 1·5 | 1 | 1·8 | 1·2 | 1·2 | 0·8 | WALKING |
| 1022 | construction | 0 | 0 | 0·0 | 0·0 | 0·0 | 0·0 | WALKING |
| 1050 | hanging bridges | 3 | 2·4 | 3·6 | 2·9 | 2·4 | 1·9 | WALKING |
| 1051 | bridge | 4·3 | 3·7 | 4·8 | 4·4 | 3·8 | 3·0 | WALKING |

**Table 4. AccessMod travel scenarios for four-wheel vehicles.** Summary of the input scenario table used to define the travel speed and method for each combination of speed, landscape, climate, and road. The 'Class' column contains individual identifiers assigned to each type of road or landcover. The 'Label' column contains the main descriptive name for each type of road or landcover. The columns under 'Speed' represent the estimated speed for the specified UI and climatic conditions. The 'Mode' column is needed in AccessMod to assign to each type of road or landcover the main transport mode, which can be on foot (WALKING), cycling (BYCYCLING), or by using motorized vehicles (MOTORIZED). For further details see: https://doc-accessmod.unepgrid.ch/display/EN/

| Class | Label | Speed (km/h) | | | | | | Mode |
| --- | --- | --- | --- | --- | --- | --- | --- | --- |
|  |  | Average  Dry | Average  Wet | Upper  Dry | Upper  Wet | Lower  Dry | Lower  Wet |  |
| 10 | Tree cover | 3·2 | 2·4 | 3·8 | 2·9 | 2·6 | 1·9 | WALKING |
| 20 | Shrubland | 3·6 | 2·7 | 4·3 | 3·2 | 2·9 | 2·2 | WALKING |
| 30 | Grassland | 4·8 | 3·6 | 5·7 | 4·3 | 3·9 | 2·9 | WALKING |
| 40 | Cropland | 3·2 | 2·7 | 3·8 | 3·2 | 2·6 | 2·2 | WALKING |
| 50 | Built-up | 5 | 3·7 | 6·0 | 4·4 | 4·0 | 3·0 | WALKING |
| 60 | Bare sparse vegetation | 3 | 2·2 | 3·6 | 2·6 | 2·4 | 1·8 | WALKING |
| 70 | Snow and Ice | 1·6 | 1·2 | 1·9 | 1·4 | 1·3 | 1·0 | WALKING |
| 80 | Permanent water bodies | 0 | 0 | 0·0 | 0·0 | 0·0 | 0·0 | WALKING |
| 90 | Herbaceous wetland | 0·5 | 0 | 0·6 | 0·0 | 0·4 | 0·0 | WALKING |
| 1001 | trunk | 55 | 48·75 | 60·6 | 52·9 | 49·4 | 44·6 | MOTORIZED |
| 1002 | trunk link | 55 | 48·75 | 60·6 | 52·9 | 49·4 | 44·6 | MOTORIZED |
| 1003 | primary | 55 | 48·75 | 60·6 | 52·9 | 49·4 | 44·6 | MOTORIZED |
| 1005 | secondary | 50 | 45 | 57·5 | 50·6 | 42·5 | 39·4 | MOTORIZED |
| 1006 | secondary link | 50 | 45 | 57·5 | 50·6 | 42·5 | 39·4 | MOTORIZED |
| 1007 | tertiary | 39·38 | 30·63 | 43·3 | 36·5 | 35·5 | 24·8 | MOTORIZED |
| 1008 | tertiary link | 39·38 | 30·63 | 43·3 | 36·5 | 35·5 | 24·8 | MOTORIZED |
| 1009 | road | 20 | 15 | 23·9 | 17·9 | 16·1 | 12·1 | MOTORIZED |
| 1011 | residential | 35 | 27·5 | 39·3 | 34·1 | 30·7 | 16·6 | MOTORIZED |
| 1012 | living street | 20 | 15 | 23·9 | 17·9 | 16·1 | 12·1 | MOTORIZED |
| 1013 | service | 35 | 27·5 | 39·3 | 34·1 | 30·7 | 16·6 | MOTORIZED |
| 1014 | track | 35 | 27·5 | 39·3 | 34·1 | 30·7 | 16·6 | MOTORIZED |
| 1015 | unclassified | 35 | 27·5 | 39·3 | 34·1 | 30·7 | 16·6 | MOTORIZED |
| 1016 | bridleway | 4·3 | 3·7 | 4·8 | 4·2 | 3·8 | 3·2 | WALKING |
| 1017 | pedestrian | 4·3 | 3·7 | 4·8 | 4·2 | 3·8 | 3·2 | WALKING |
| 1018 | path | 4·3 | 3·7 | 4·8 | 4·2 | 3·8 | 3·2 | WALKING |
| 1019 | footway | 4·3 | 3·7 | 4·8 | 4·2 | 3·8 | 3·2 | WALKING |
| 1020 | cycleway | 4·3 | 3·7 | 4·8 | 4·2 | 3·8 | 3·2 | WALKING |
| 1021 | steps | 1·5 | 1 | 1·8 | 1·2 | 1·2 | 0·8 | WALKING |
| 1022 | construction | 0 | 0 | 0·0 | 0·0 | 0·0 | 0·0 | WALKING |
| 1050 | hanging bridges | 3 | 2·4 | 3·6 | 2·9 | 2·4 | 1·9 | WALKING |
| 1051 | bridge | 4·3 | 3·7 | 4·8 | 4·2 | 3·8 | 3·2 | WALKING |

**Table 5. Population coverage according to syndrome for the time thresholds of mild and moderate envenomation**. Both scenarios represent the wet season and the average speed for motorcycle as the main transport method.

| District | Population covered n (%) | | | |
| --- | --- | --- | --- | --- |
|  | **Haemotoxic syndrome** | | **Neurotoxic syndrome** | |
|  | 180 min. (mild) | 360 min. (moderate) | 30 min. (mild) | 60 min. (moderate) |
| Dhanusa | 785 146 (99·07) | 785 146 (99·07) | 505 754 (63·82) | 780 053 (98·43) |
| Mahottari | 630 276 (98·43) | 630 325 (98·44) | 240 904 (37·62) | 589 105 (92·00) |
| Sarlahi | 778 016 (99·48) | 778 224 (99·50) | **0 (0·00)** | **30 763 (3·93)** |
| Bara | 646 162 (99·17) | 646 162 (99·17) | 163 696 (25·12) | 513 849 (78·87) |
| Chitawan | 391 079 (98·67) | 392 488 (99·03) | 158 132 (39·90) | 314 332 (79·31) |
| Makwanpur | 320 930 (99·11) | 321 089 (99·16) | **3018 (0·93)** | **51 344 (15·86)** |
| Parsa | 1 000 853 (99·14) | 1 000 853 (99·14) | 400 333 (39·66) | 958 404 (94·94) |
| Rautahat | 688 160 (99·42) | 688 160 (99·42) | **0 (0·00)** | **172 (0·02)** |
| Morang | 566 980 (98·00) | 566 985 (98·01) | **12 451 (2·15)** | **151 786 (26·24)** |
| Sunsari | 945 634 (99·01) | 945 634 (99·01) | 123 184 (12·90) | 460 288 (48·19) |
| Jhapa | 448 722 (98·94) | 448 722 (98·94) | 181 179 (39·95) | 366 451 (80·80) |
| Saptari | 628 941 (99·16) | 628 941 (99·16) | **0 (0·00)** | **22 946 (3·62)** |
| Siraha | 810 342 (99·24) | 810 342 (99·24) | 311 195 (38·11) | 763 244 (93·47) |
| Udayapur | 314 507 (99·54) | 314 507 (99·54) | **325 (0·10)** | **40 820 (12·92)** |
| Kanchanpur | 599 178 (98·83) | 599 178 (98·83) | 122 109 (20·14) | 529 387 (87·32) |
| Kailali | 667 313 (96·73) | 683 374 (99·06) | 307 335 (44·55) | 582 267 (84·41) |
| Banke | 386 473 (99·53) | 387 178 (99·71) | 234 177 (60·31) | 332 839 (85·72) |
| Bardiya | 333 400 (98·44) | 333 400 (98·44) | 14 021 (4·14) | 173 078 (51·11) |
| Surkhet | 231 441 (97·49) | 235 865 (99·36) | 45 307 (19·09) | 97 380 (41·02) |
| Dang | 474 223 (99·06) | 476 937 (99·63) | 137 241 (28·67) | 388 232 (81·10) |
| Kapilbastu | 659 609 (99·44) | 659 618 (99·44) | 344 106 (51·87) | 647 543 (97·62) |
| Nawalparasi | 542 684 (99·03) | 542 691 (99·03) | 356 534 (65·06) | 504 511 (92·06) |
| Rupandehi | 796 099 (99·43) | 796 099 (99·43) | 431 666 (53·91) | 795 678 (99·37) |

**Table 6. Classification example of 9-class vulnerability for the combination of average snakebite risk (SBR) and average travel time risk (TTR) for motorcycle, wet season, and neurotoxic syndrome**. The coloured cells represent the 3-class vulnerability colour scheme used in figures 2a, 3, 4c, and 4d in the main text, which symbolises the population in low (blue), medium (yellow), high (red) vulnerability classes.

| Travel time  risk (TTR) | Snakebite risk (SBR) | | |  | |
| --- | --- | --- | --- | --- | --- |
|  | Lower | Medium | Higher | **Total TTR** | **TTR %** |
| Mild | 2 332 961 | 1 982 164 | 8573 | **4 323 698** | 31·93 |
| Moderate | 2 616 861 | 2 189 575 | 17 007 | **4 823 444** | 35·62 |
| Severe | 2 340 619 | 1 879 004 | 175 360 | **4 394 984** | 32·45 |
| Total SBR | **7 290 441** | **6 050 744** | **200 941** | **13 542 125** |  |
| SBR % | 53·84 | 44·68 | 1·48 |  |  |

| **Travel time**  **component** | **Vulnerability scenario** | | | **Vulnerability**  **class** |
| --- | --- | --- | --- | --- |
|  | **Low: n (%) ⯆** | **Medium: n (%) ⚫** | **High: n (%) ⯅** |  |
| FWH | 0 (0) | 661 (0) | 23 384 (0·17) | High |
|  | 4467 (0·03) | 229 304 (1·69) | 822 614 (6·05) | Medium |
|  | 13 538 171 (99·97) | 13 312 141 (98·30) | 12 755 877 (93·78) | Low |
| FDH | 0 (0) | 86 (0) | 6448 (0·05) | High |
|  | 3360 (0·02) | 207 420 (1·53) | 769 494 (5·64) | Medium |
|  | 13 585 037 (99·98) | 13 380 359 (98·47) | 12 871 143 (94·31) | Low |
| FWN | 409 862 (3·03) | 1 927 345 (14·23) | 4 467 704 (32·85) | High |
|  | 3 099 384 (22·89) | 4 549 852 (33·60) | 4 639 111 (34·11) | Medium |
|  | 10 033 392 (74·09) | 7 064 908 (52·17) | 4 495 060 (33·05) | Low |
| FDN | **330 206 (2·43)** | **1 460 759 (10·75)** | **3 374 106 (24·72)** | **High** |
|  | 2 200 135 (16·19) | 3 841 793 (28·27) | 4 373 520 (32·05) | Medium |
|  | 11 058 057 (81·38) | 8 285 313 (60·98) | 5 899 459 (43·23) | Low |
| TWH | 0 (0) | 733 (0·01) | 45 136 (0·33) | High |
|  | **4727 (0·03)** | **247 833 (1·83)** | **930 078 (6·84)** | **Medium** |
|  | 13 537 911 (99·97) | 13 293 540 (98·16) | 12 626 662 (92·83) | Low |
| TDH | 0 (0) | 149 (0) | 14 525 (0·11) | High |
|  | 3360 (0·02) | 217 797 (1·60) | 800 764 (5·87) | Medium |
|  | 13 585 037 (99·98) | 13 369 919 (98·40) | 12 831 796 (94·03) | Low |
| TWN | 497 003 (3·67) | 2 811 097 (20·76) | 6 859 176 (50·43) | High |
|  | 4 276 979 (31·58) | 5 120 068 (37·81) | 4 266 934 (31·37) | Medium |
|  | 8 768 656 (64·75) | 5 610 940 (41·43) | 2 475 766 (18·20) | Low |
| TDN | 446 644 (3·29) | 2 360 647 (17·37) | 3 365 089 (24·66) | High |
|  | 3 694 540 (27·19) | 4 713 609 (34·69) | 4 274 651 (31·32) | Medium |
|  | 9 447 214 (69·52) | 6 513 608 (47·94) | 6 007 346 (44·02) | Low |
| MWH | 0 (0) | 534 (0) | 3280 (0·02) | High |
|  | 3477 (0·03) | 205 179 (1·52) | 774 939 (5·70) | Medium |
|  | **13 539 181 (99·97)** | **13 336 412 (98·48)** | **12 823 676 (94·28)** | **Low** |
| MDH | 0 (0) | 5 (0) | 990 (0·01) | High |
|  | 3360 (0·02) | 201 593 (1·48) | 751 534 (5·51) | Medium |
|  | 13 585 057 (99·98) | 13 386 286 (98·52) | 12 894 580 (94·49) | Low |
| MWN | 424 157 (3·13) | 2 071 372 (15·30) | 4 468 096 (32·85) | High |
|  | 3 261 652 (24·08) | 4 538 768 (33·52) | 4 589 955 (33·74) | Medium |
|  | 9 856 849 (72·78) | 6 931 985 (51·19) | 4 543 844 (33·41) | Low |
| MDN | 311 816 (2·29) | 1 435 532 (10·56) | 3 578 337 (26·22) | High |
|  | 1 896 519 (13·96) | 3 781 037 (27·83) | 4 367 694 (32·00) | Medium |
|  | 11 380 082 (83·75) | 8 371 316 (61·61) | 5 701 073 (41·77) | Low |

**Table 7. Rural population vulnerability.** Rural population of the Terai in each of the 36 vulnerability scenarios and 3 vulnerability classes. **Abbreviations:**

FWH: four-wheeled vehicle, wet, haemotoxic

FDH: four-wheeled vehicle, dry, haemotoxic

FWN: four-wheeled vehicle, wet, neurotoxic

FDN: four-wheeled vehicle, dry, neurotoxic

TWH: tempo, wet, haemotoxic

TDH: tempo, dry, haemotoxic

TWN: tempo, wet, neurotoxic

TDN: tempo, dry, neurotoxic

MWH: motorcycle, wet, haemotoxic

MDH: motorcycle, dry, haemotoxic

MWN: motorcycle, wet, neurotoxic

MDN: motorcycle, dry, neurotoxic


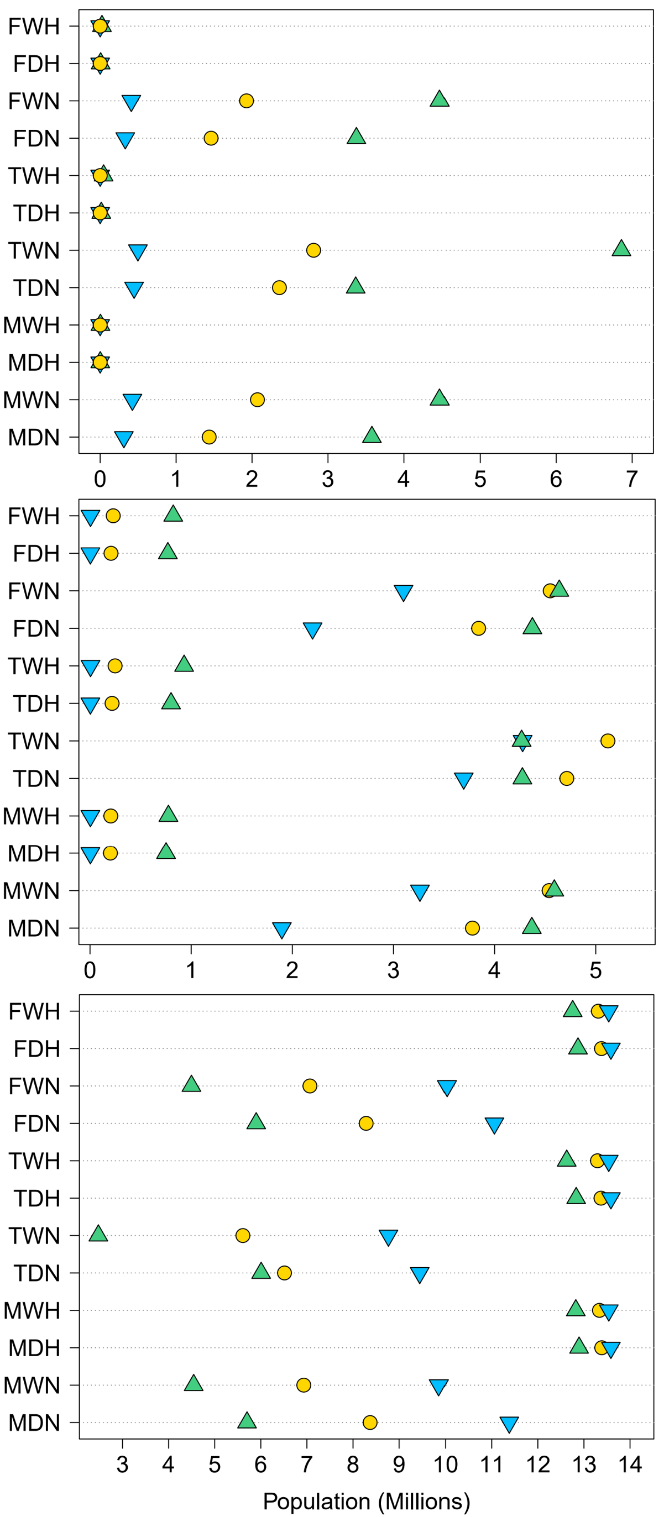


**Figure 1. Population per vulnerability class according to the different scenarios.**

Top panel: high vulnerability class

Middle panel: medium vulnerability class

Bottom panel: low vulnerability class

**⚫**: medium vulnerability scenario

⯆: low vulnerability scenario

**⯅**: high vulnerability scenario

The arrows show an example of how to read the values from each table row into the corresponding plot and model.

**Table 8. Population in the high vulnerability class, ranked according to the population size of non-overlapping catchment areas.** The ID represents the facilities in figure 3 in the main paper. The last column represents the number of people covered by each independent, 60-minute catchment.

| District | VDC | Health facility name | ID | Non-ovelapping coverage (pop.) | Independent coverage (pop.) |
| --- | --- | --- | --- | --- | --- |
| Saptari | Rajbiraj N.P. | Gajendra Narayan Zonal Hospital | A | 585 122 | 585 122 |
| Sarlahi | Netraganj | Nawalpur Snakebite Treatment Center | B | 490 135 | 490 135 |
| Morang | Rangeli | Rangeli District Hospital | C | 276 767 | 276 767 |
| Udayapur | Katari | Katari Hospital | D | 63 119 | 66 279 |
| Morang | Biratnagar N.P. | Koshi Zonal Hospital | E | 42 849 | 274 880 |
| Siraha | Lahan N.P. | Ram Kumar Muraraka Hospital | F | 30 869 | 323 844 |
| Udayapur | Triyuga N.P. | Samudayik Sarpadansha Upachar Kendra (Bhulke) | G | 19 611 | 95 895 |
| Sarlahi | Malangawa N.P. | Malangawa District Hospital | H | 5732 | 460 194 |
| Mahottari | Gauribas | Samudaik Sarpadansha Upachar Kendra | I | 5643 | 199 995 |
| Morang | Sijuwa | Samudaik Sarpadansha Upachar Kendra | J | 5364 | 159 805 |
| Siraha | Badharamal | Community Snakebite Treatment Center | K | 464 | 56 502 |


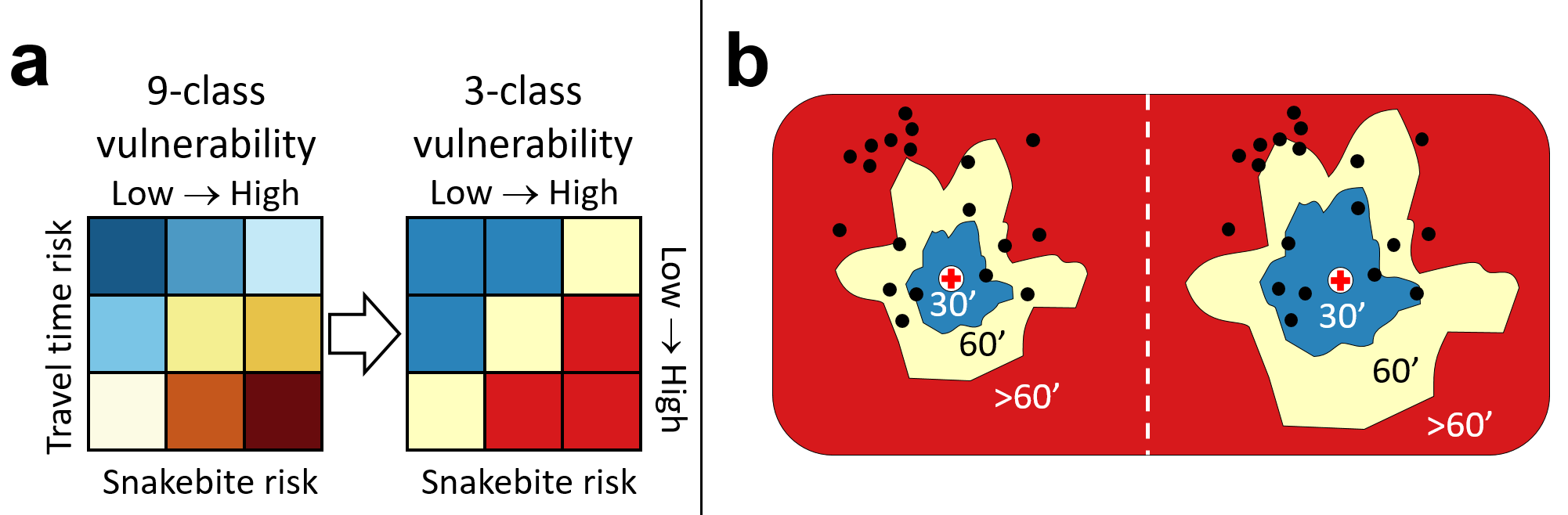


**Figure 2.** (a) Simplification code from 9-class to 3-class vulnerability colour scheme. (b) Comparison of the 3-class vulnerability coverage of the population (black dots) depending on high (left) or low (right) vulnerability scenarios.


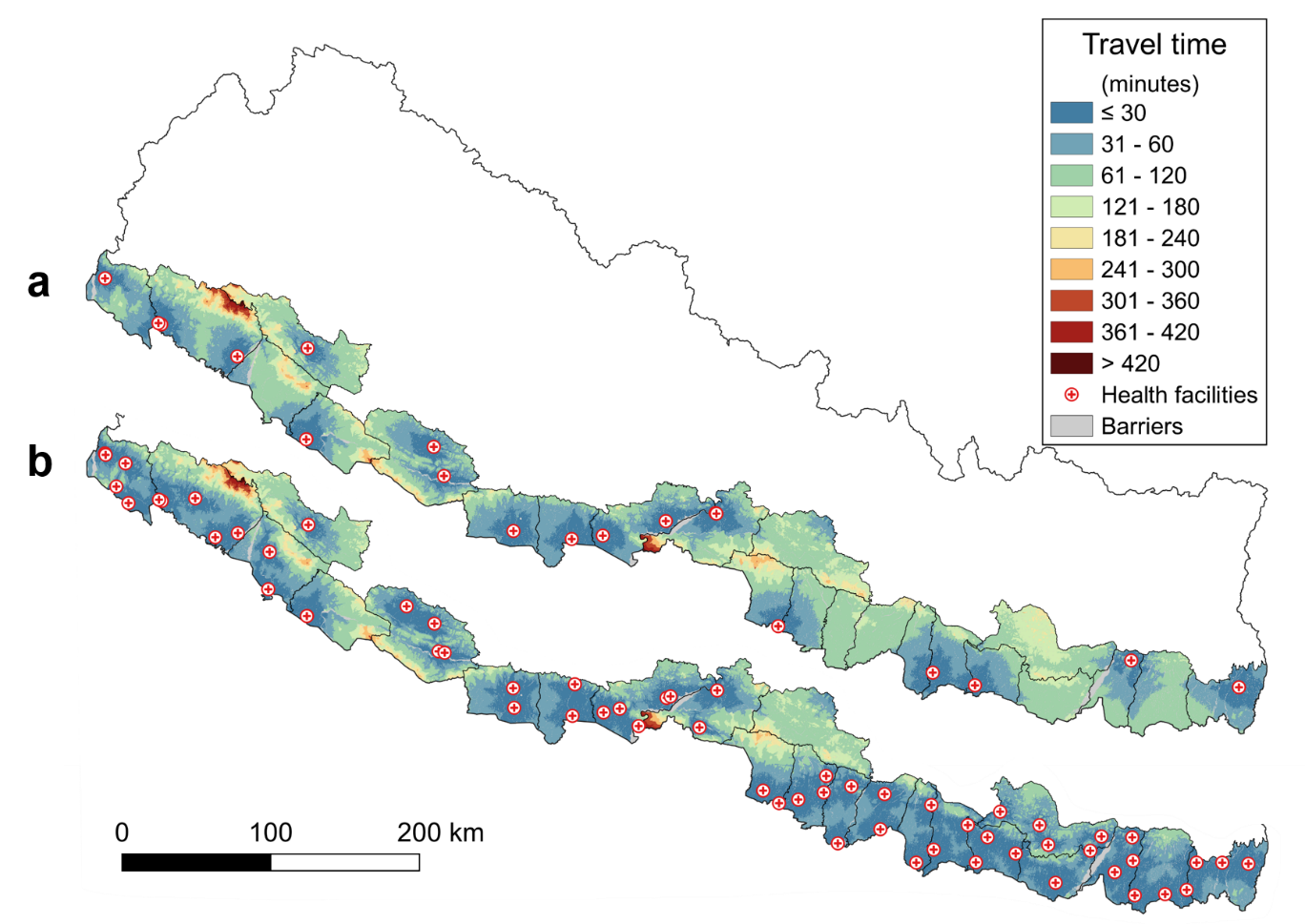


**Figure 3.** Continuous travel time raster of the Terai for a medium vulnerability scenario (combination of average snakebite and travel time risks), including motorcycle as main transport method, and wet season for the neurotoxic (**a**) and haemotoxic (**b**) syndromes, showing in each case the facilities able to treat them. **Source**: vector map and administrative divisions from [gadm.org](https://gadm.org/download_country_v3.html), projected in the local WGS 84/UTM zone 45 N coordinate reference system in QGIS 3.24 ([qgis.org](https://qgis.org/en/site/))


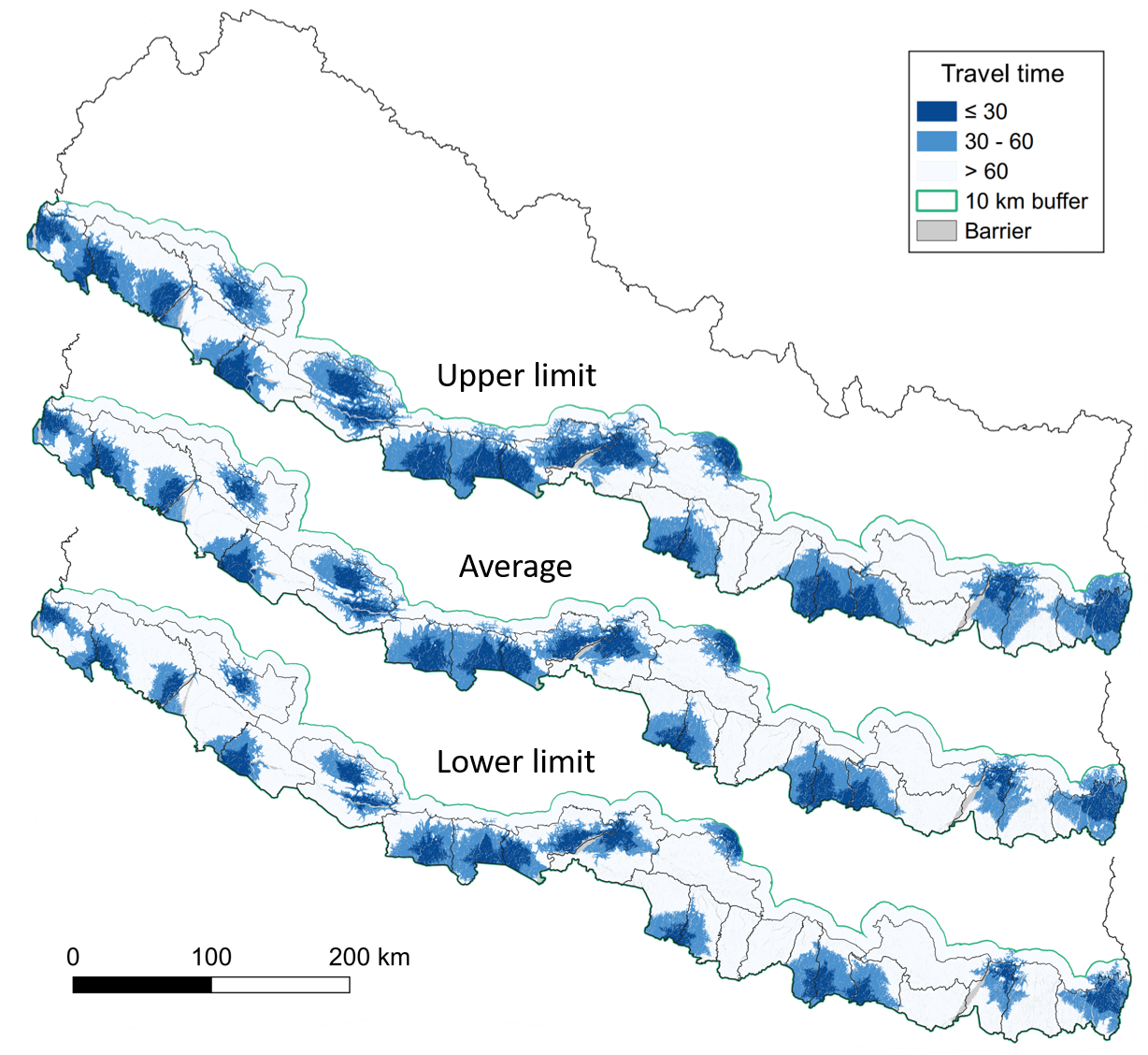


**Figure 4**. Travel time catchment areas for facilities treating the neurotoxic syndrome for a medium vulnerability scenario, combining motorcycle as main transport method, and wet season. The time thresholds represent mild, moderate, and severe risk of mortality or morbidity caused by travel-time treatment delays. **Source:** vector map and administrative divisions from [gadm.org](https://gadm.org/download_country_v3.html), projected in the local WGS 84/UTM zone 45 N coordinate reference system in QGIS 3.24 ([qgis.org](https://qgis.org/en/site/)).

**References to the supplementary appendix**

1. QGIS Geographic Information System. Open Source Geospatial Foundation Project. 3.24 ed: QGIS Development Team; 2022.

2. R-Core-Team. R: A language and environment for statistical computing. 4.1.2. ed. Vienna, Austria: R Foundation for Statistical Computing; 2021.

3. Ediriweera DS, Kasturiratne A, Pathmeswaran A, et al. Mapping the risk of snakebite in Sri Lanka - a national survey with geospatial analysis. PLoS Neglected Tropical Diseases 2016; 10(7): e0004813.

4. Ochoa C, Pittavino M, Babo Martins S, et al. Estimating and predicting snakebite risk in the Terai region of Nepal through a high-resolution geospatial and One Health approach. Scientific reports 2021; 11(1): 1-13.

5. Roll U, Feldman A, Novosolov M, et al. The global distribution of tetrapods reveals a need for targeted reptile conservation. Nature Ecology & Evolution 2017; 1(11): 1677-82.

6. Banick RS, Kawasoe Y. Measuring Inequality of Access: Modeling Physical Remoteness in Nepal. World Bank Policy Research Working Paper 2019; (8966).

7. Farr TG, Rosen PA, Caro E, et al. The shuttle radar topography mission. Reviews of geophysics 2007; 45(2).

8. Source imagery for HRSL© 2016 DigitalGlobe. Facebook connectivity lab and center for international earth science information network-CIESIN: Columbia University; 2016.

9. Ramm F, Names I, Files S, et al. OpenStreetMap data in layered GIS format. Version 06 2014; 7.

10. Zanaga D, Van De Kerchove R, De Keersmaecker W, et al. ESA WorldCover 10 m 2020 v100; 2021.

11. Trail Bridges Strategy. In: Development MoL, editor. Lalitpur: Government of Nepal; 2006.
