# Supplementary material for: Vulnerability to snakebite envenoming and access to healthcare in the Terai region of Nepal: a geospatial analysis
Source: Lancet Reg Health Southeast Asia. 2022 Nov 17;9:100103. doi: 10.1016/j.lansea.2022.100103 (PMC10306013; doi:10.1016/j.lansea.2022.100103)
Supplement: Appendix 3 [file mmc3.pdf]

High SB risk Vs FLDH_rc

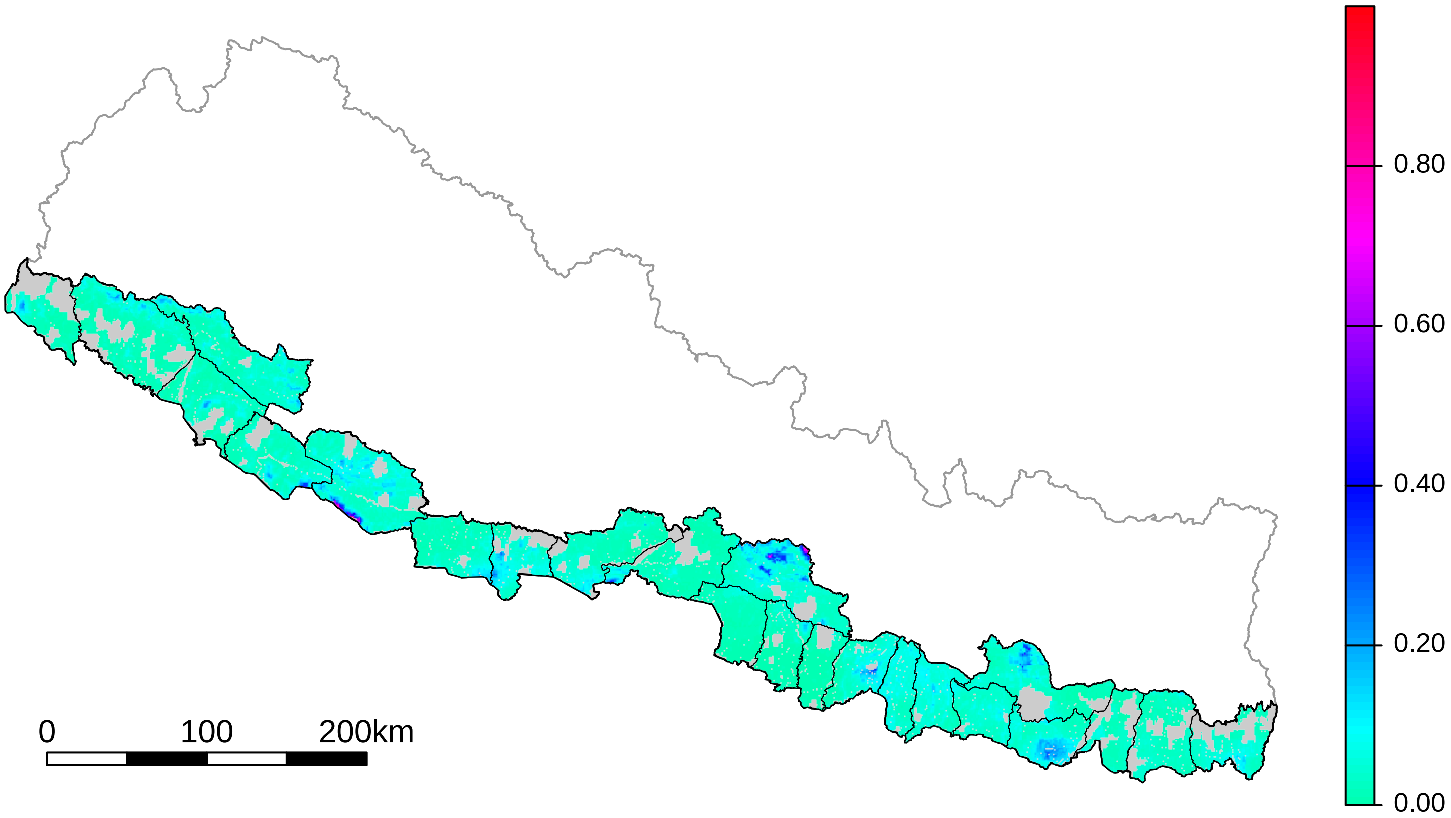

High SB risk Vs FLDN_rc

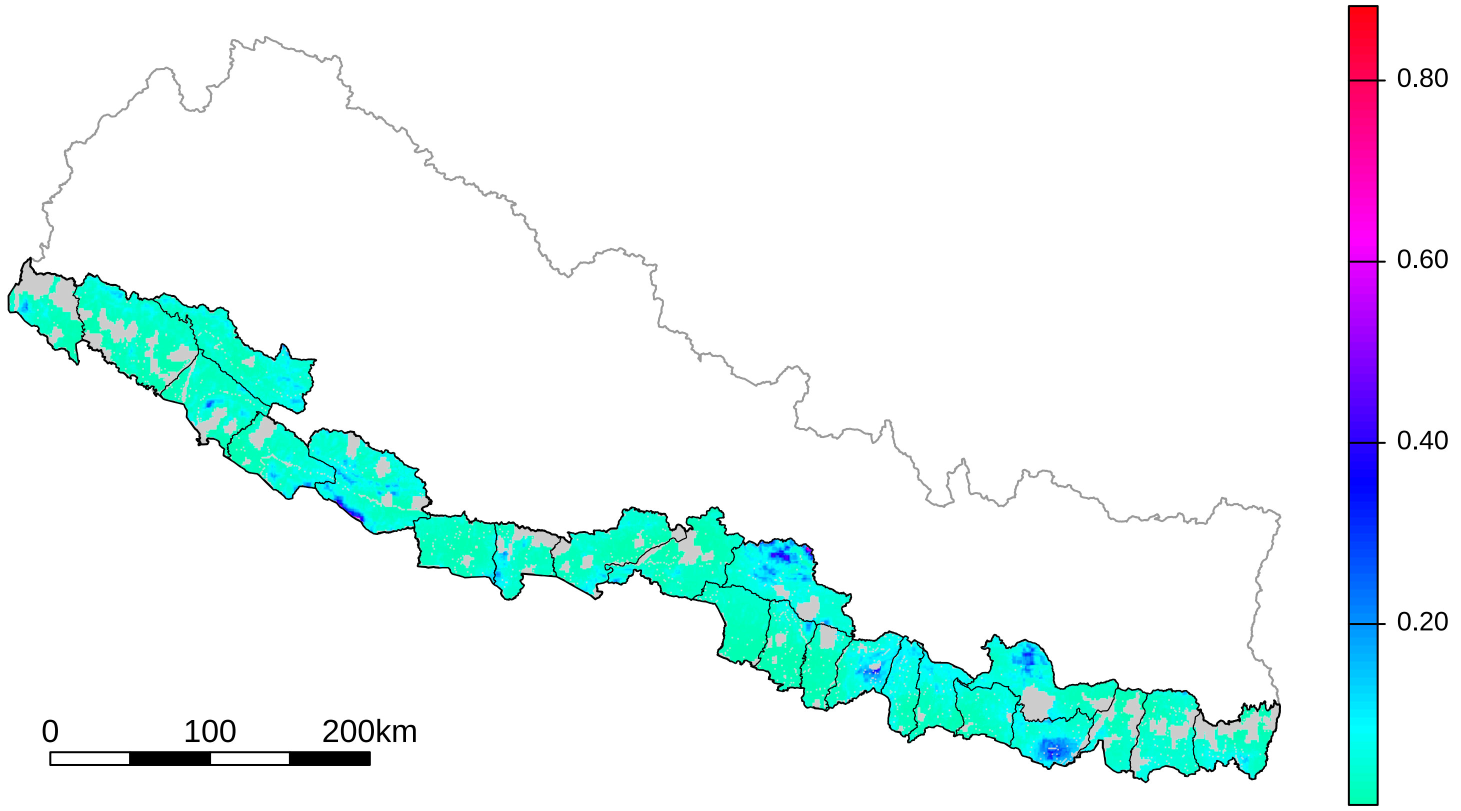

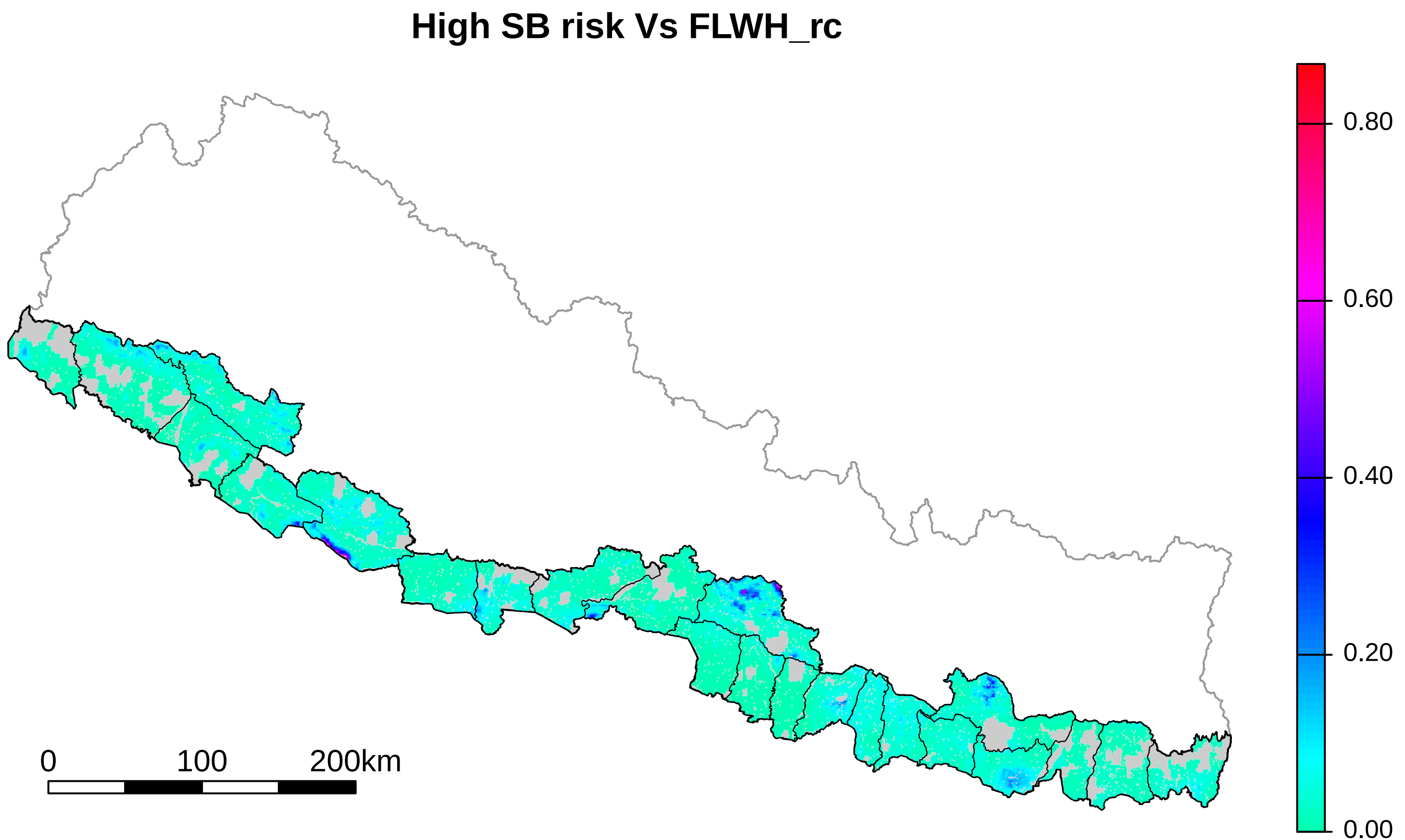

High SB risk Vs FLWH_rc
0
100
200km
0.80
0.60
0.40
0.20
0.00

High SB risk Vs FLWN_rc

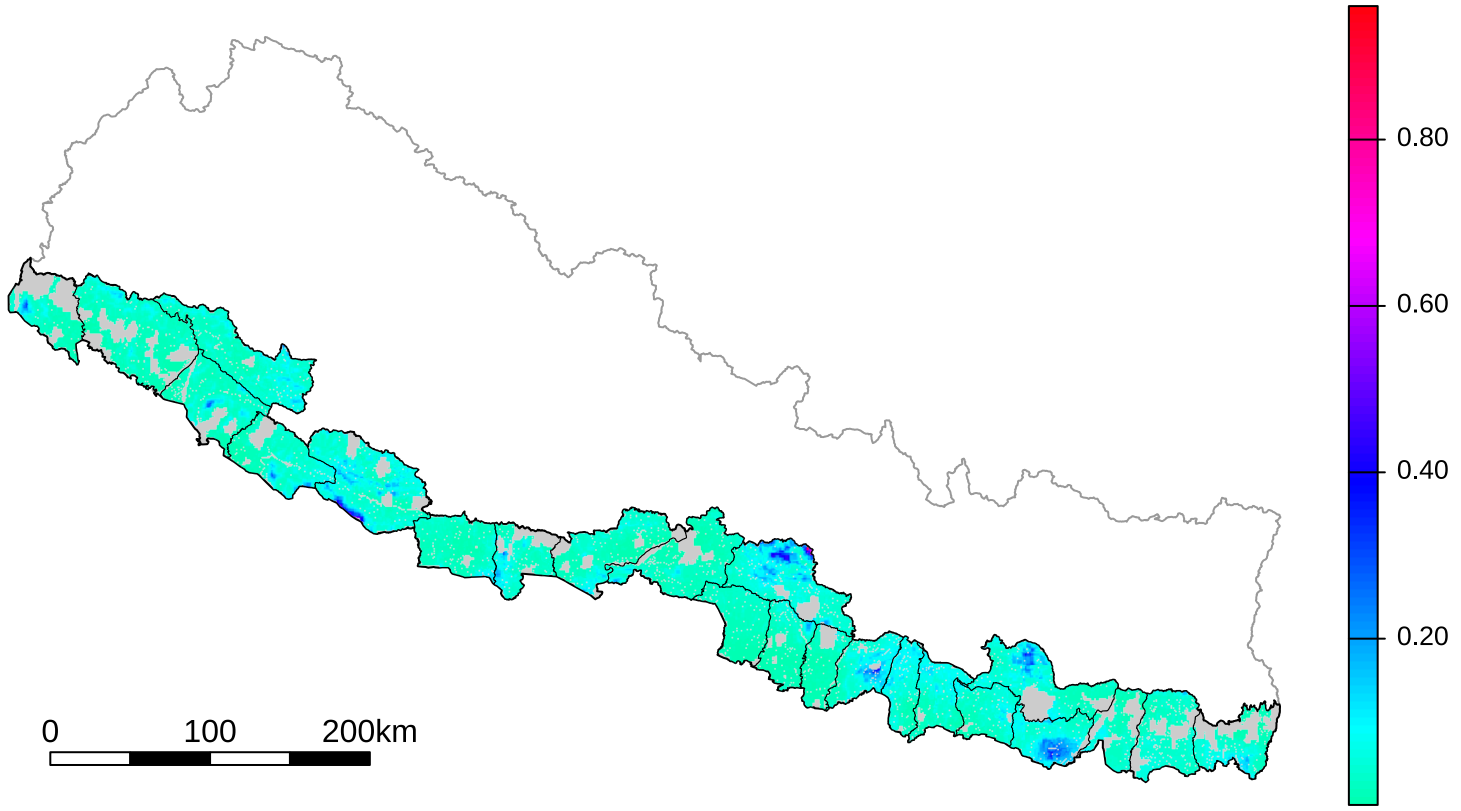

High SB risk Vs MLDH_rc

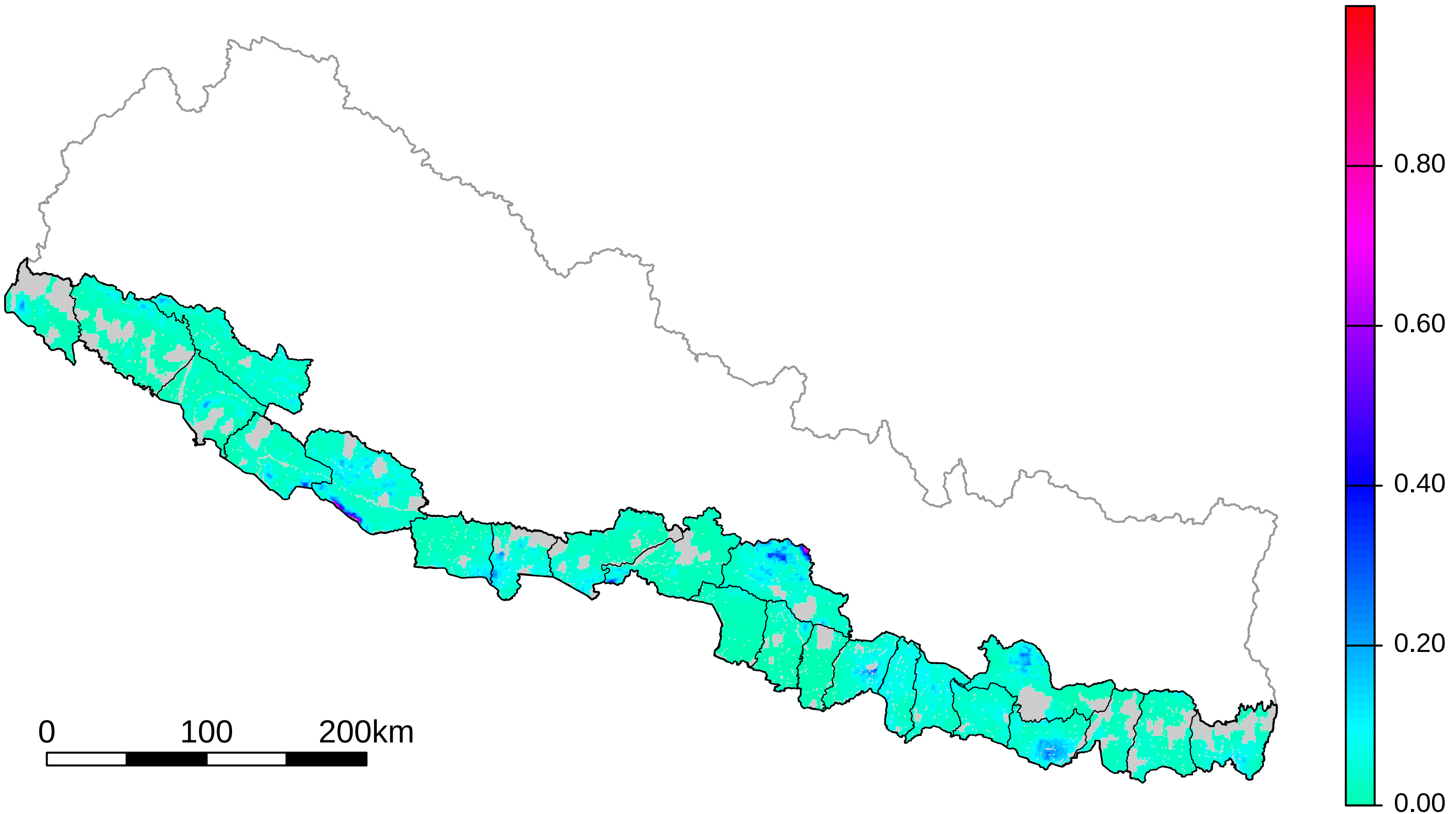

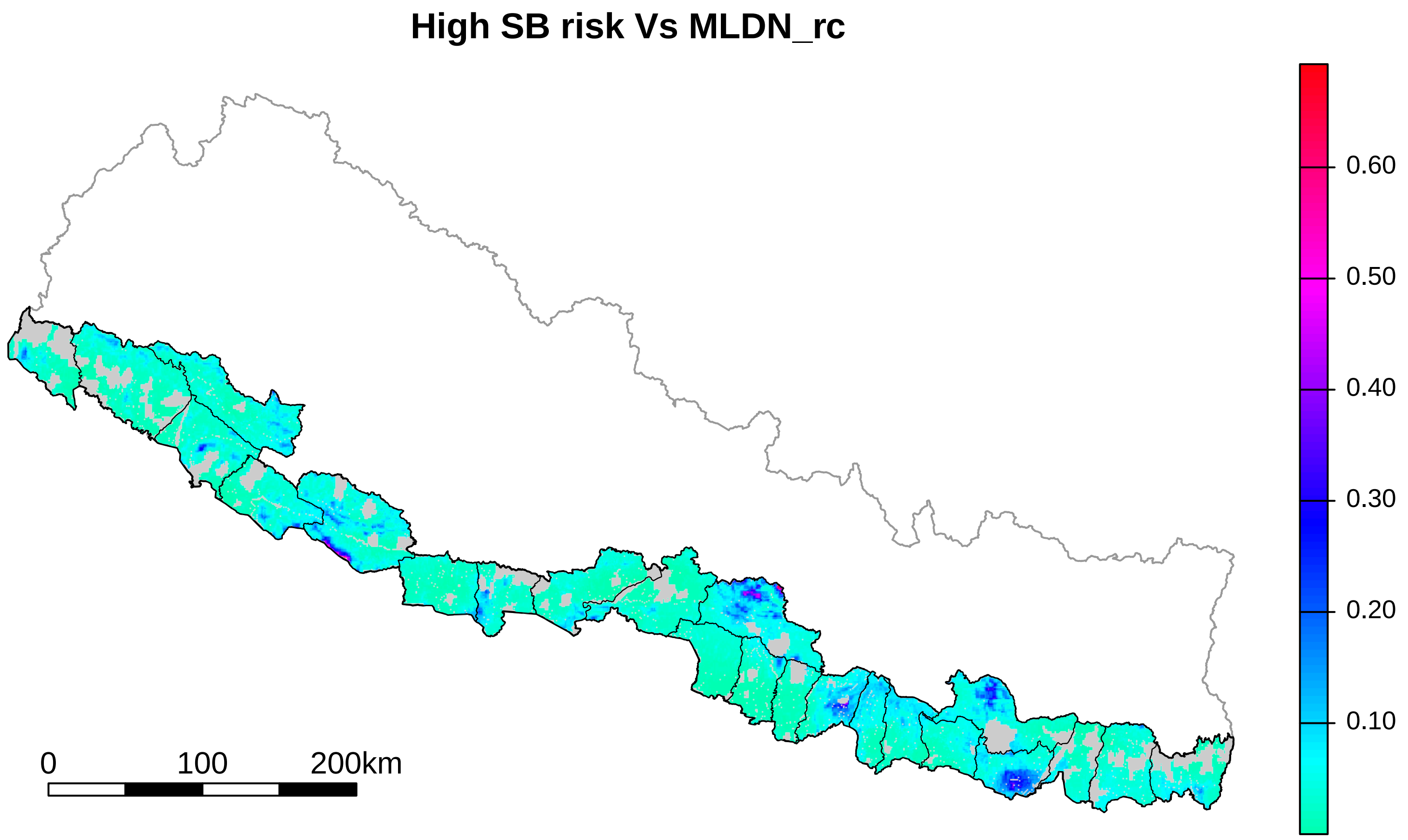

High SB risk Vs MLDN_rc
0.60
0.50
0.40
0.30
0.20
0.10
0
100
200km

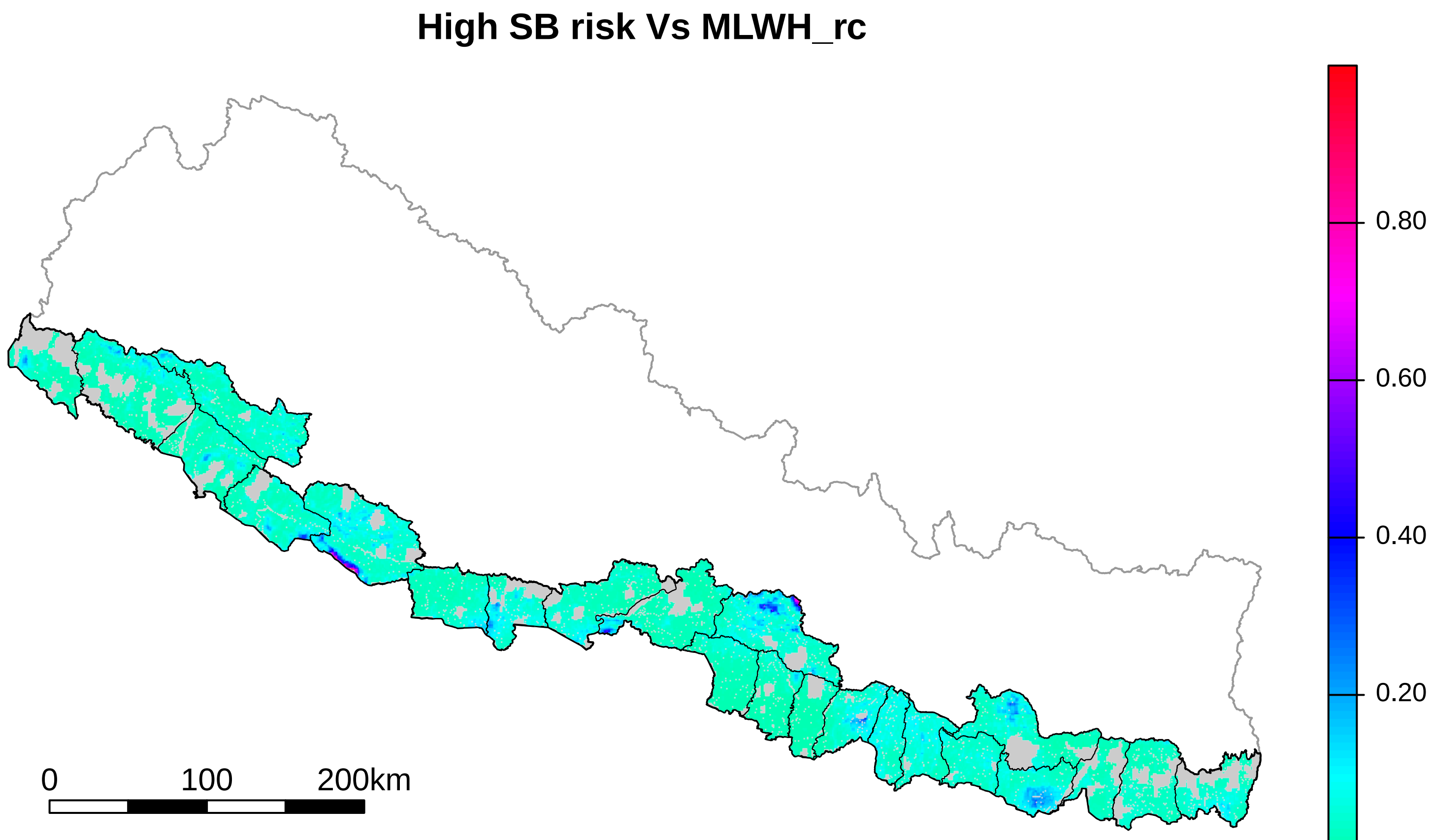
High SB risk Vs MLWH_rc
0.80
0.60
0.40
0.20
0.00
0
100
200km

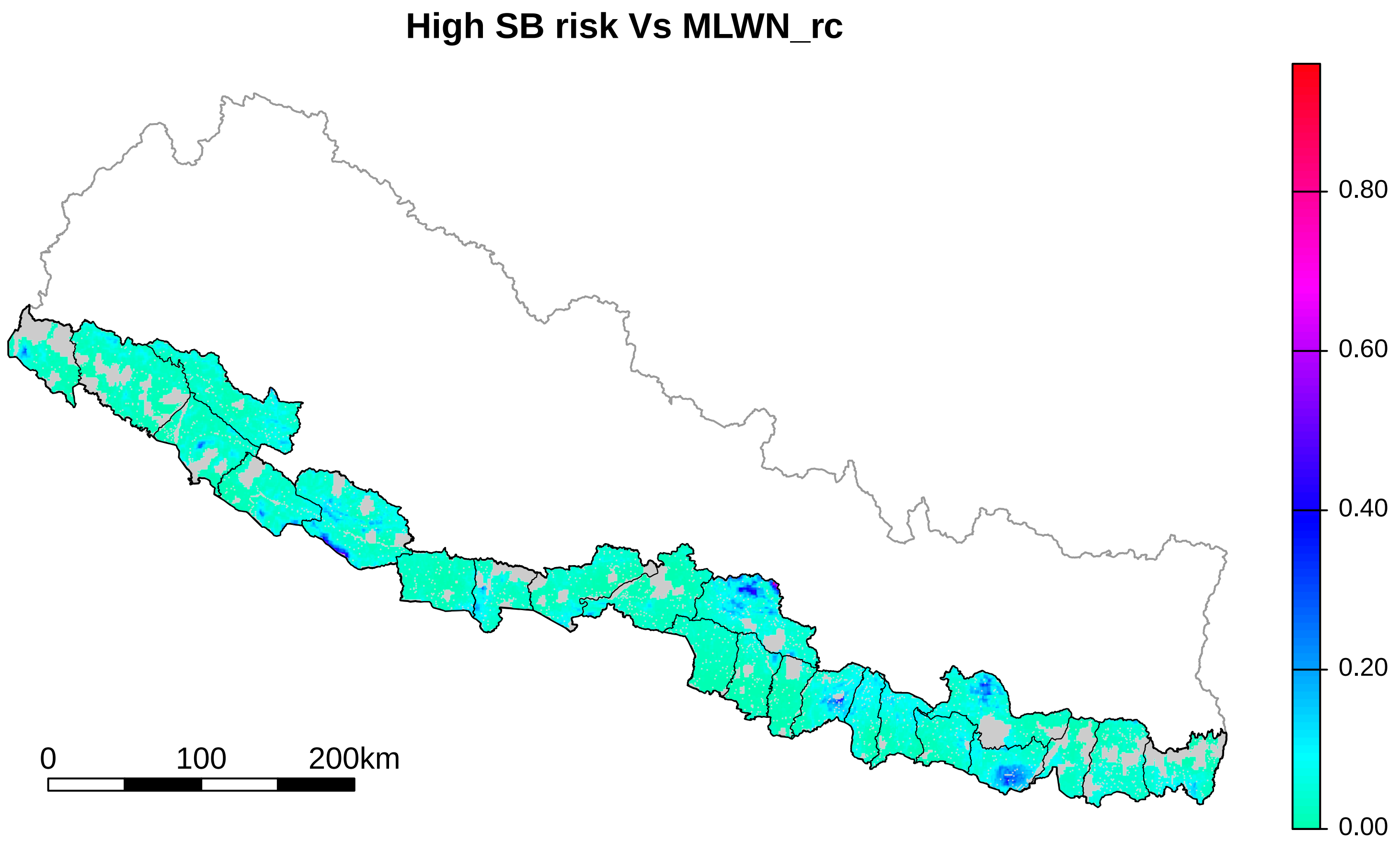

High SB risk Vs MLWN_rc
0
100
200km
0.80
0.60
0.40
0.20
0.00

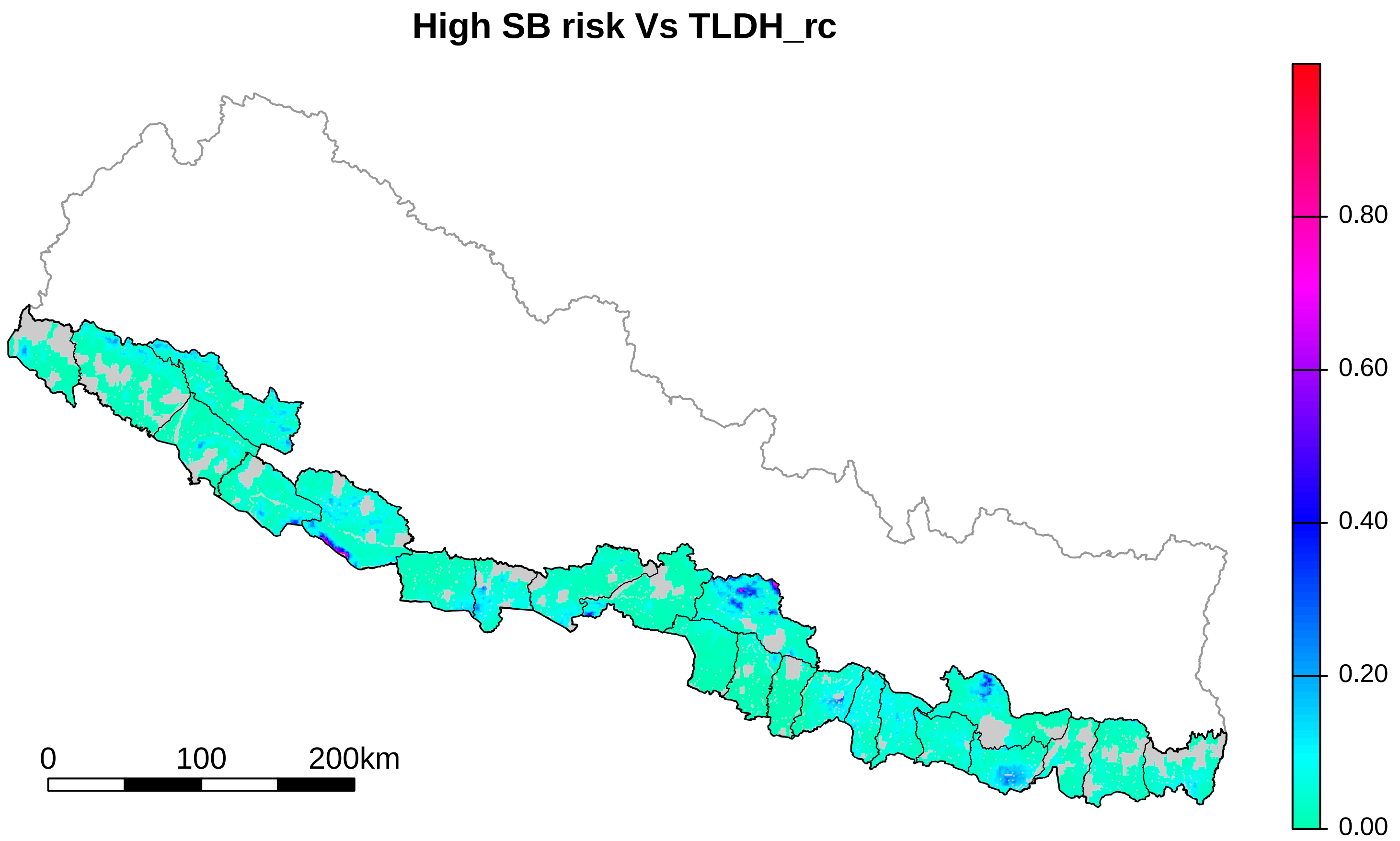
High SB risk Vs TLDH_rc
0
100
200km
0.80
0.60
0.40
0.20
0.00

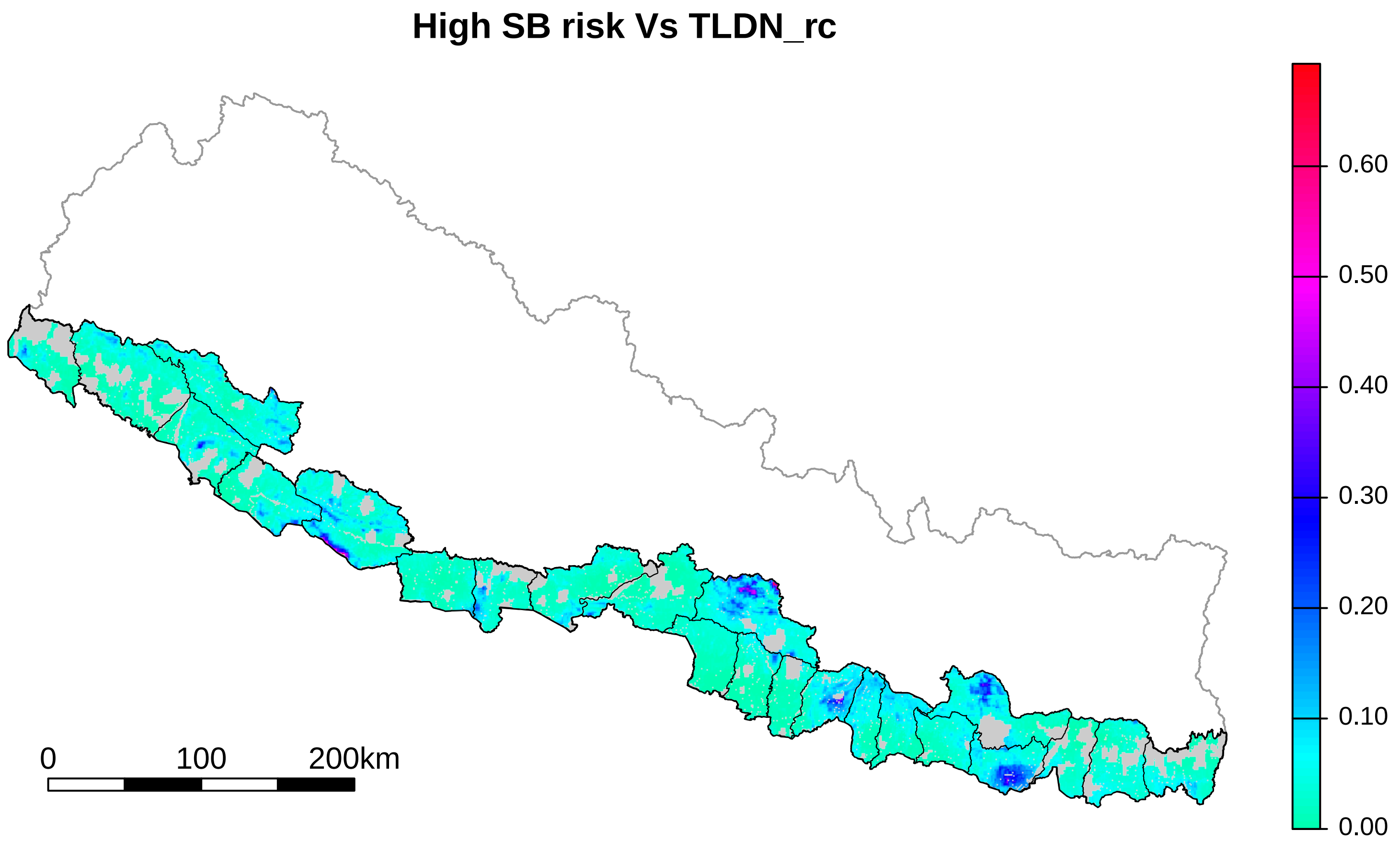
High SB risk Vs TLDN_rc
0
100
200km
0.60
0.50
0.40
0.30
0.20
0.10
0.00

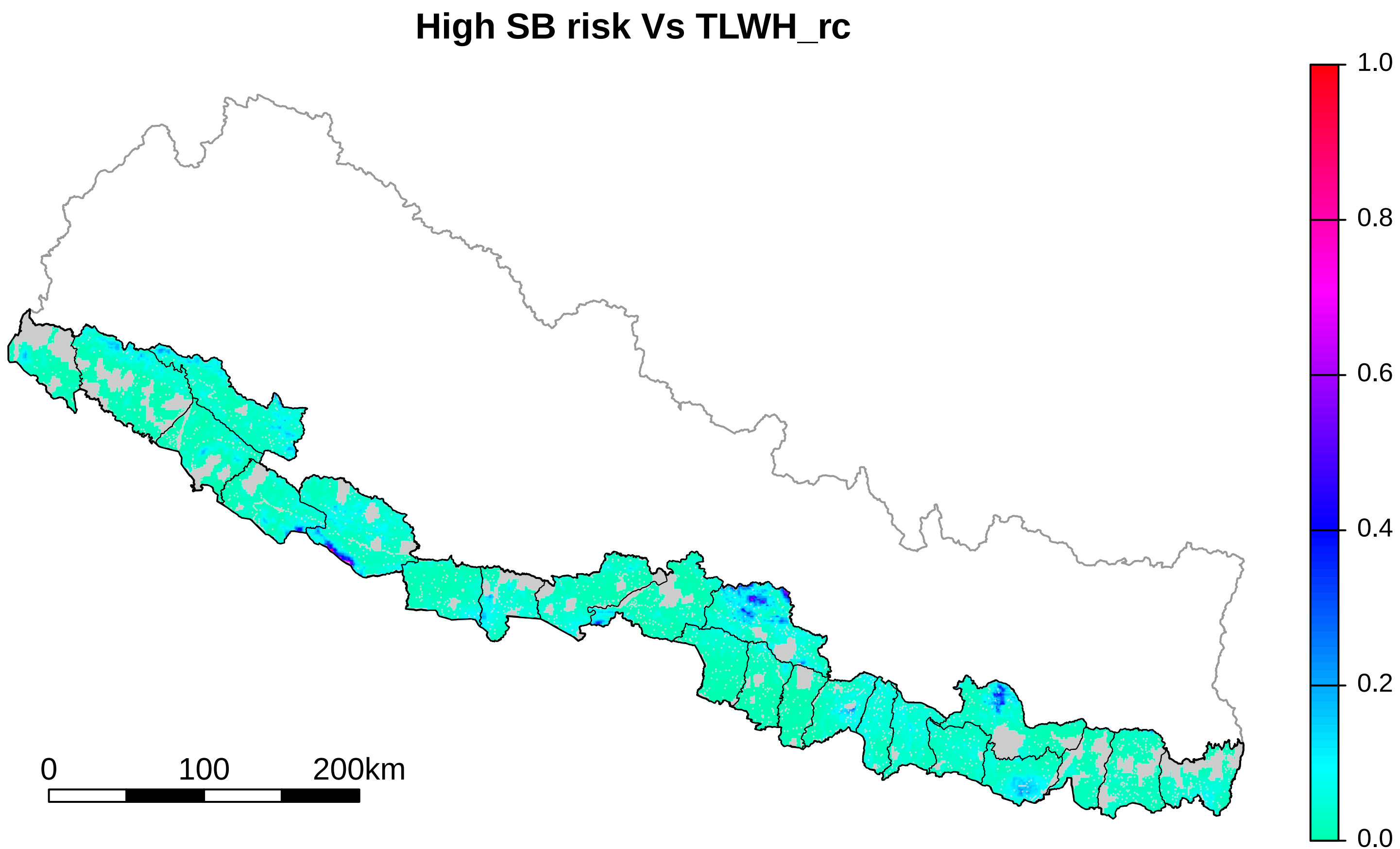
High SB risk Vs TLWH_rc
0
100
200km
1.0
0.8
0.6
0.4
0.2
0.0

High SB risk Vs TLWN_rc

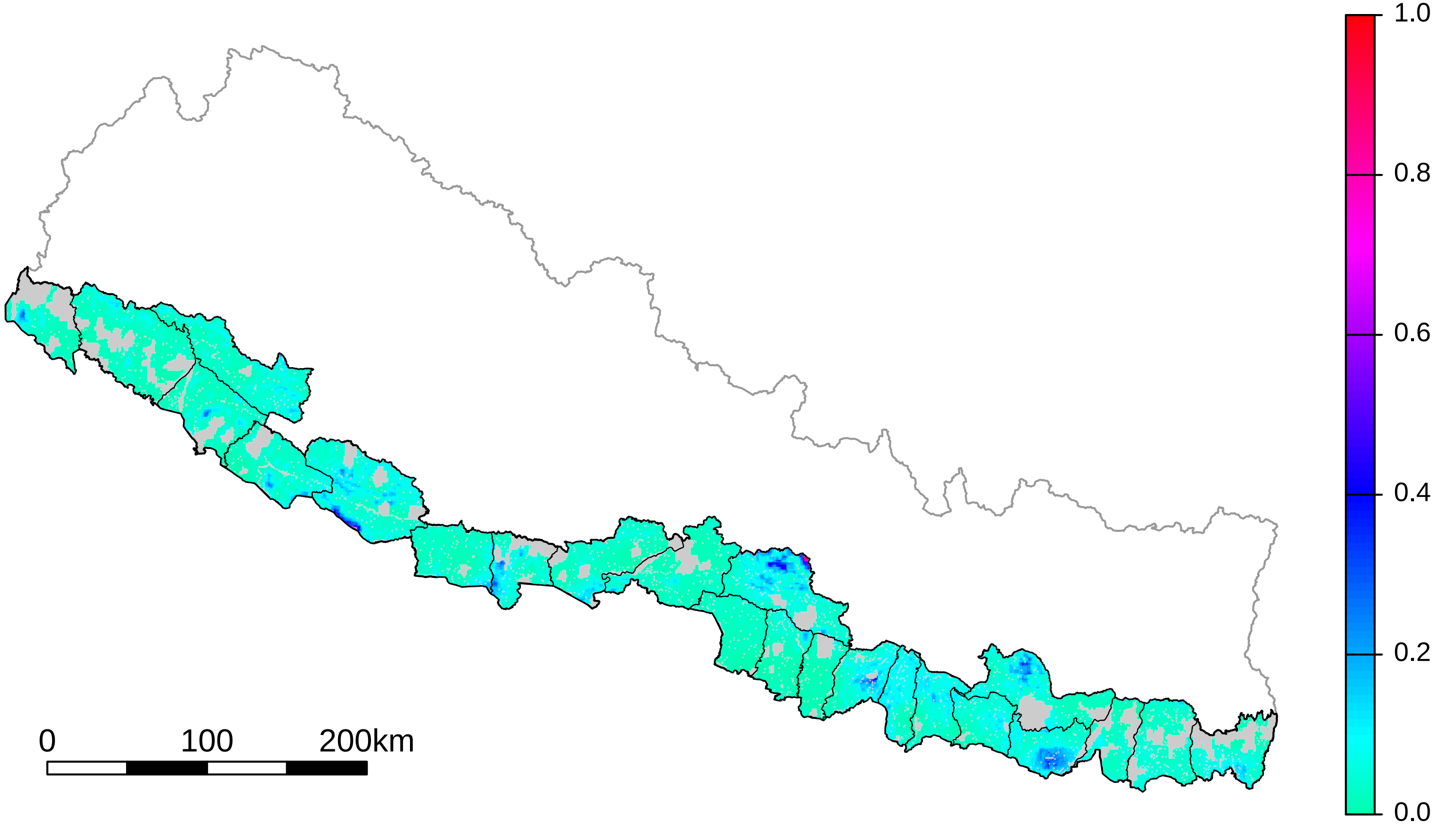

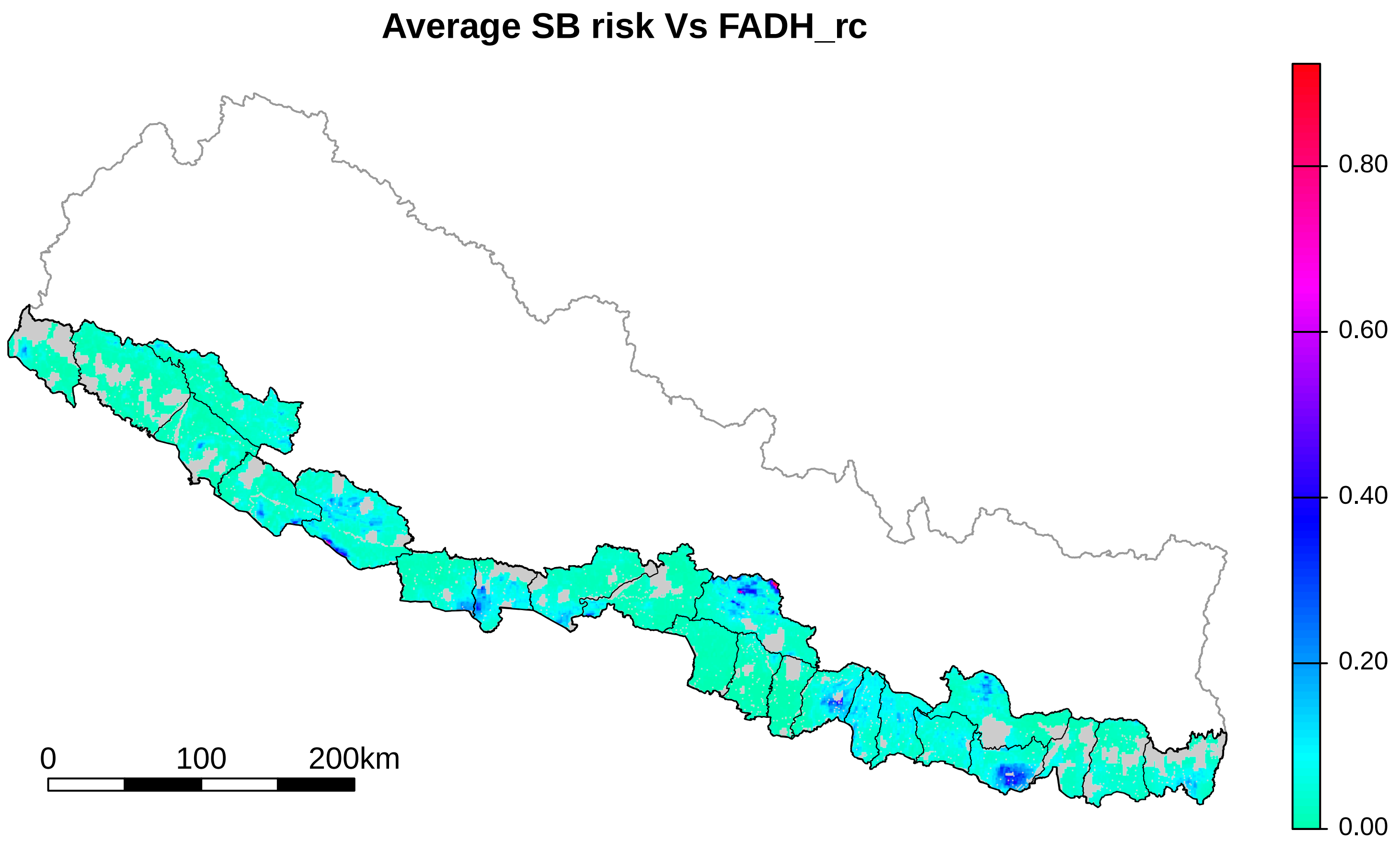

Average SB risk Vs FADH_rc
0
100
200km
0.80
0.60
0.40
0.20
0.00

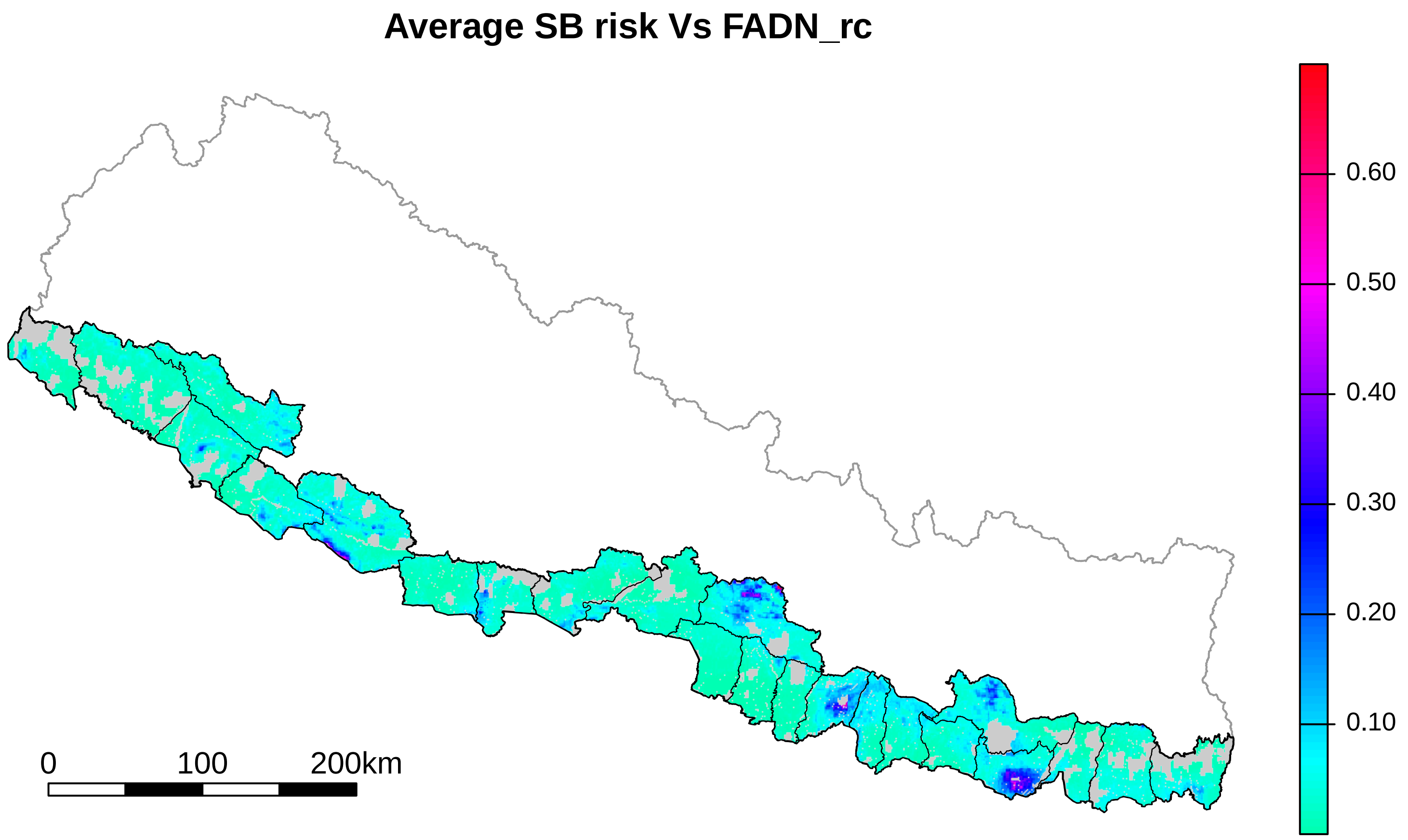

Average SB risk Vs FADN_rc
0.60
0.50
0.40
0.30
0.20
0.10
0
100
200km

Average SB risk Vs FAWH_rc

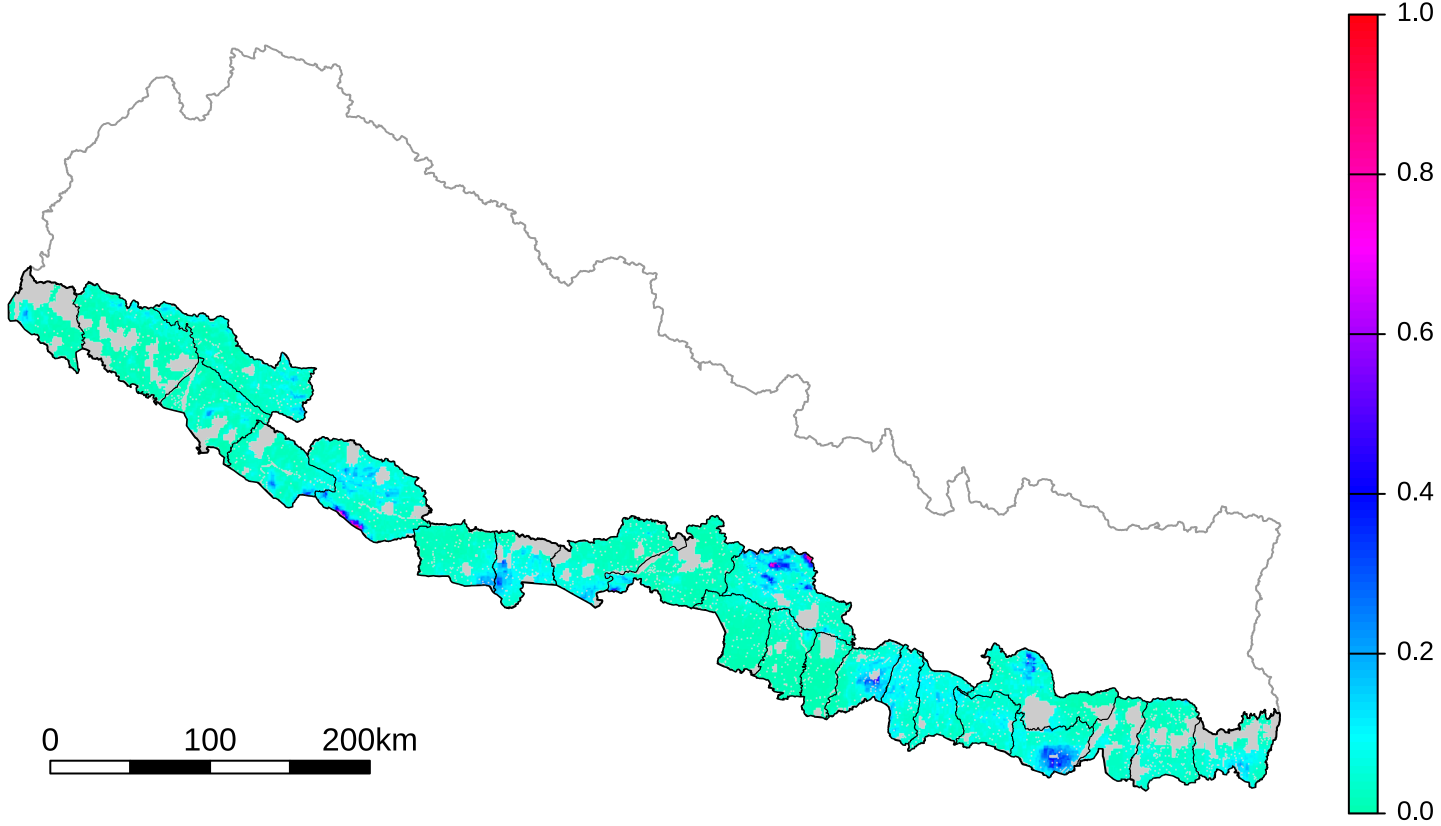

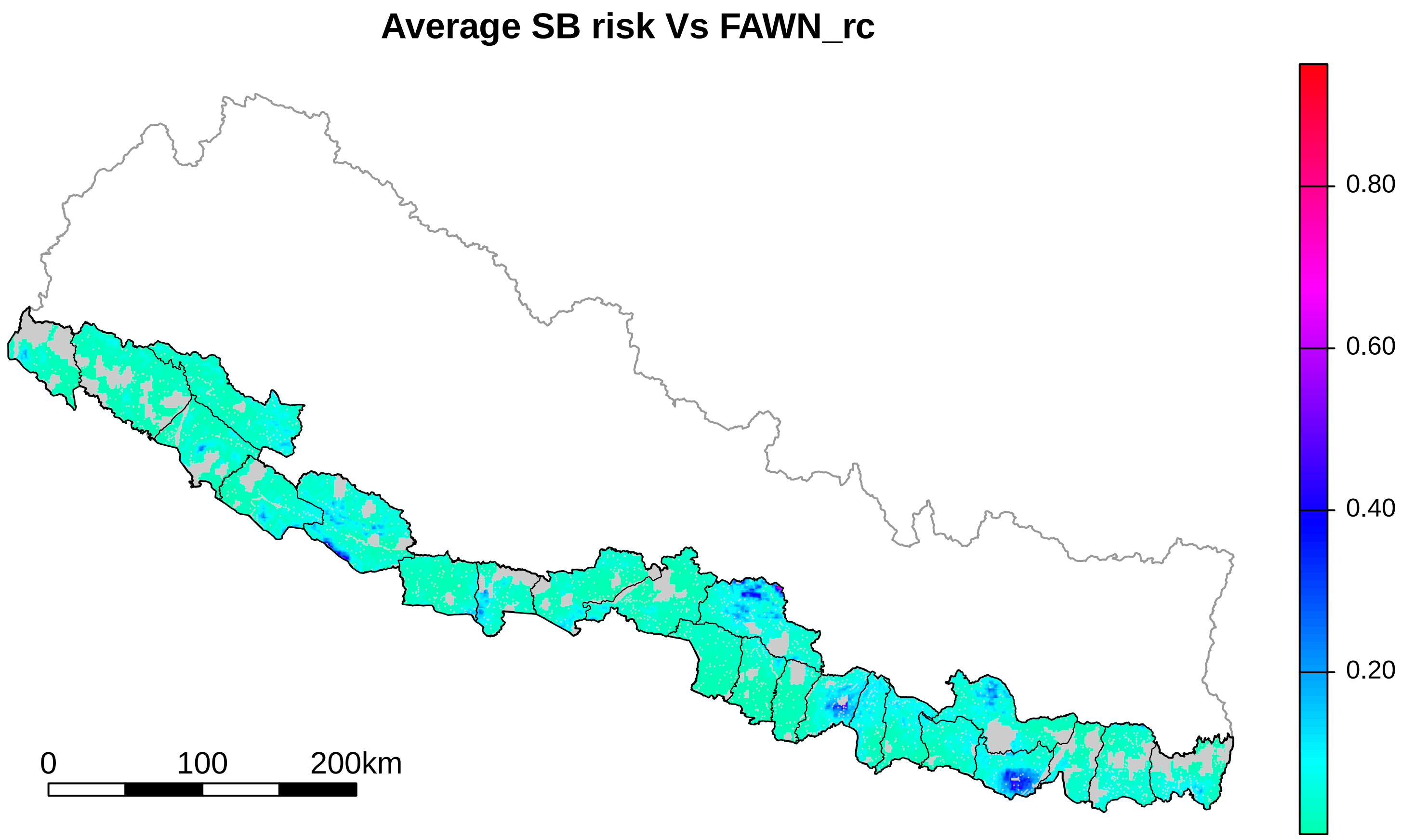
Average SB risk Vs FAWN_rc
0
100
200km
0.80
0.60
0.40
0.20

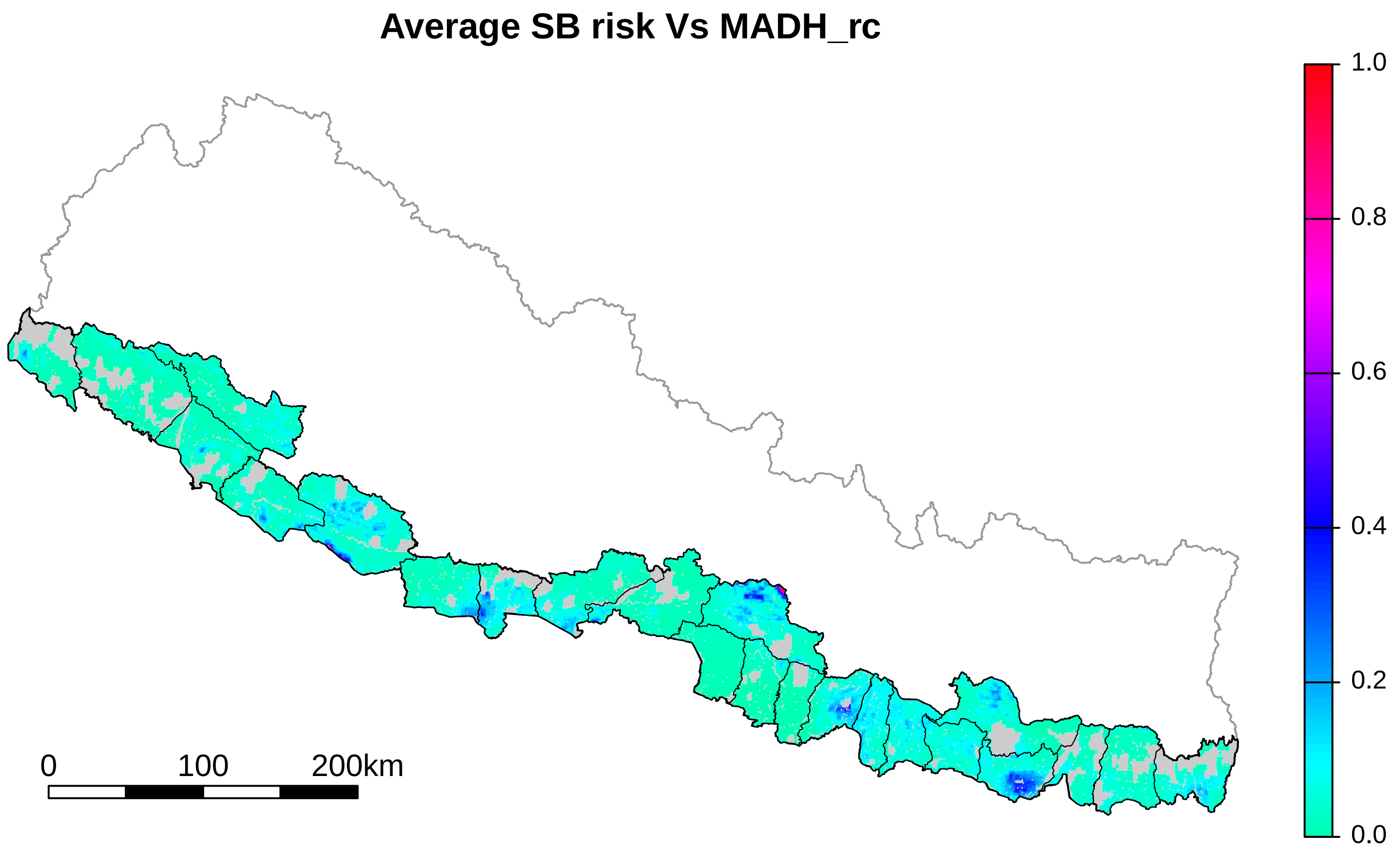

Average SB risk Vs MADH_rc
1.0
0.8
0.6
0.4
0.2
0.0
0
100
200km

Average SB risk Vs MADN_rc

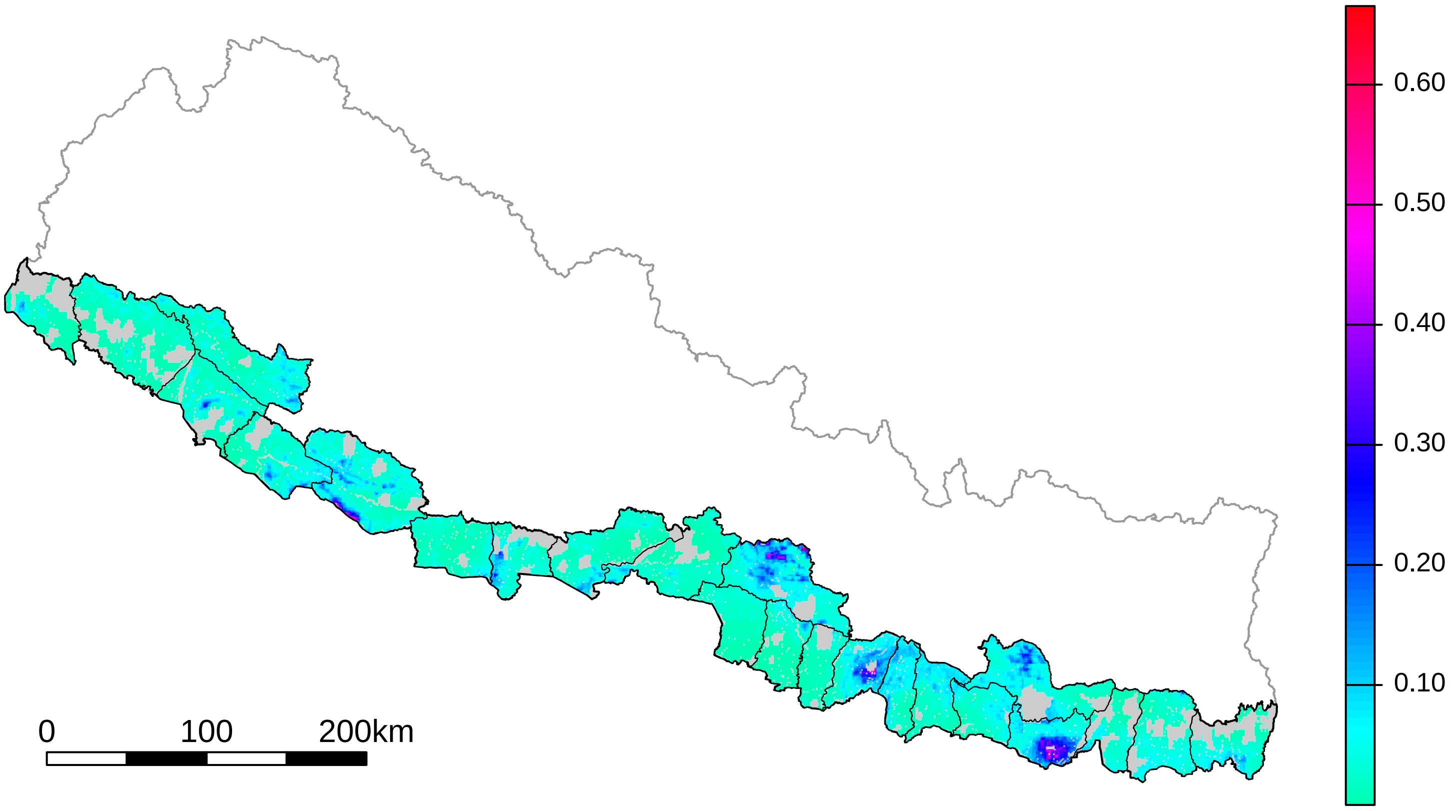

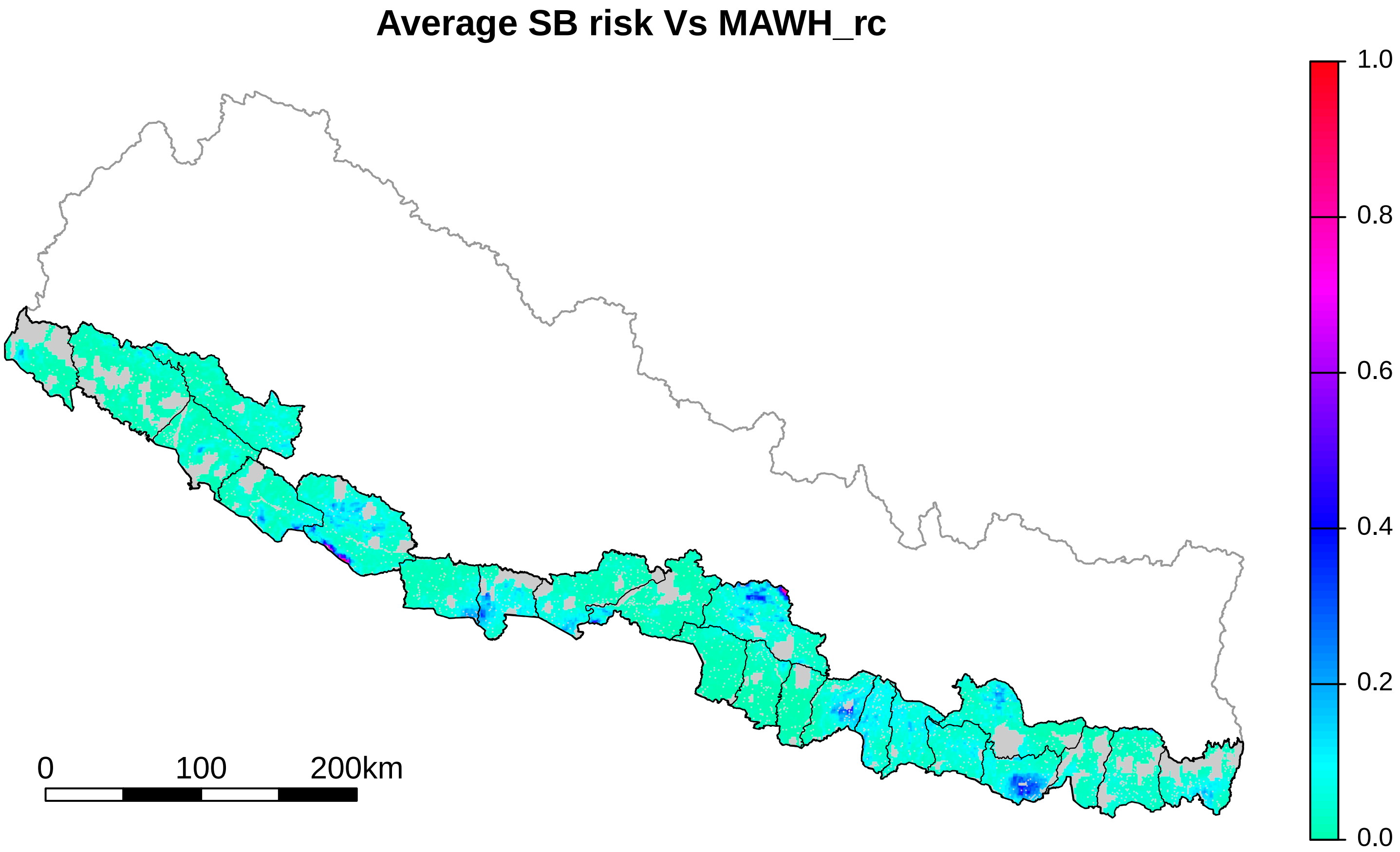

Average SB risk Vs MAWH_rc
1.0
0.8
0.6
0.4
0.2
0.0
0
100
200km

Average SB risk Vs MAWN_rc

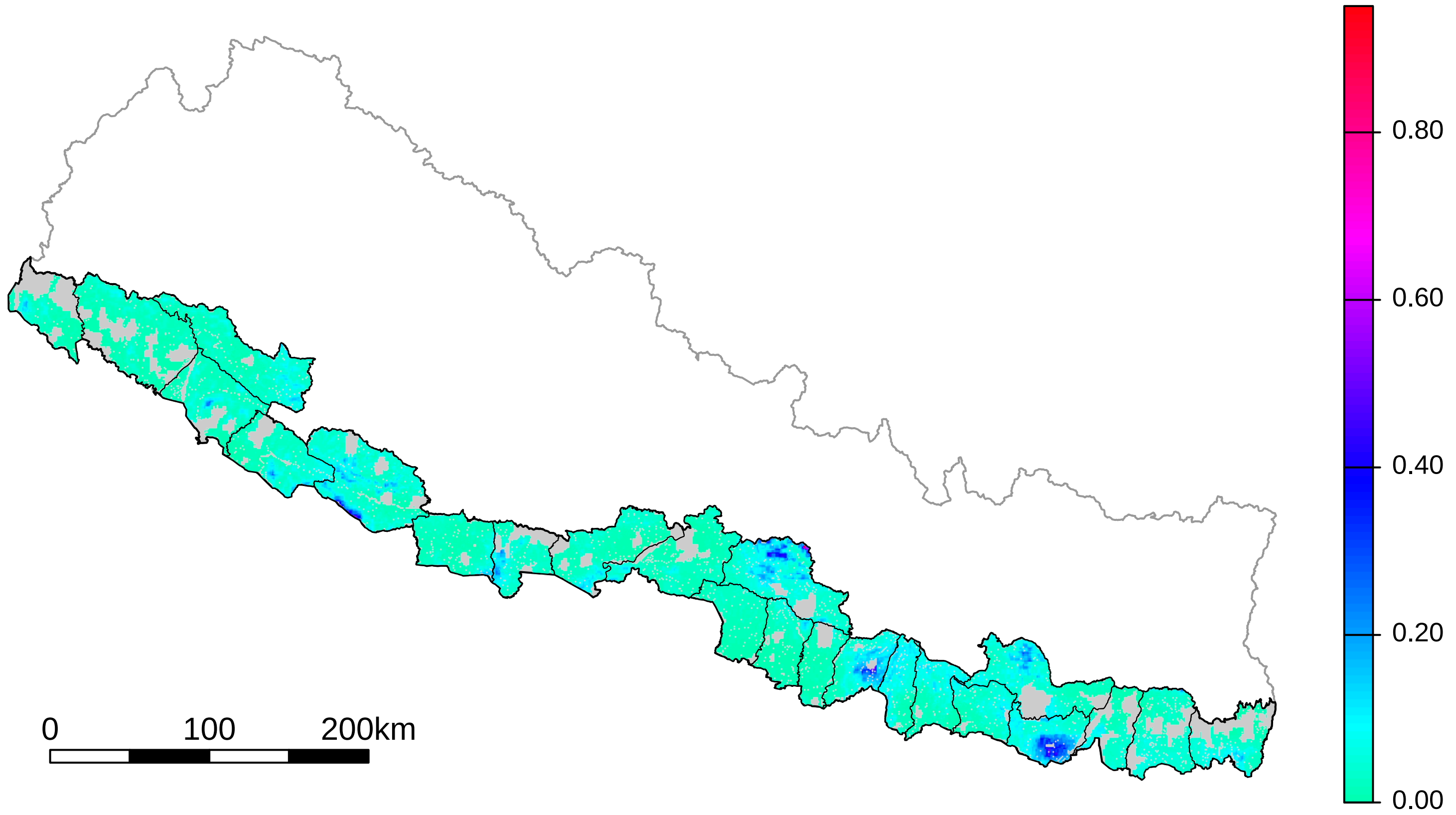

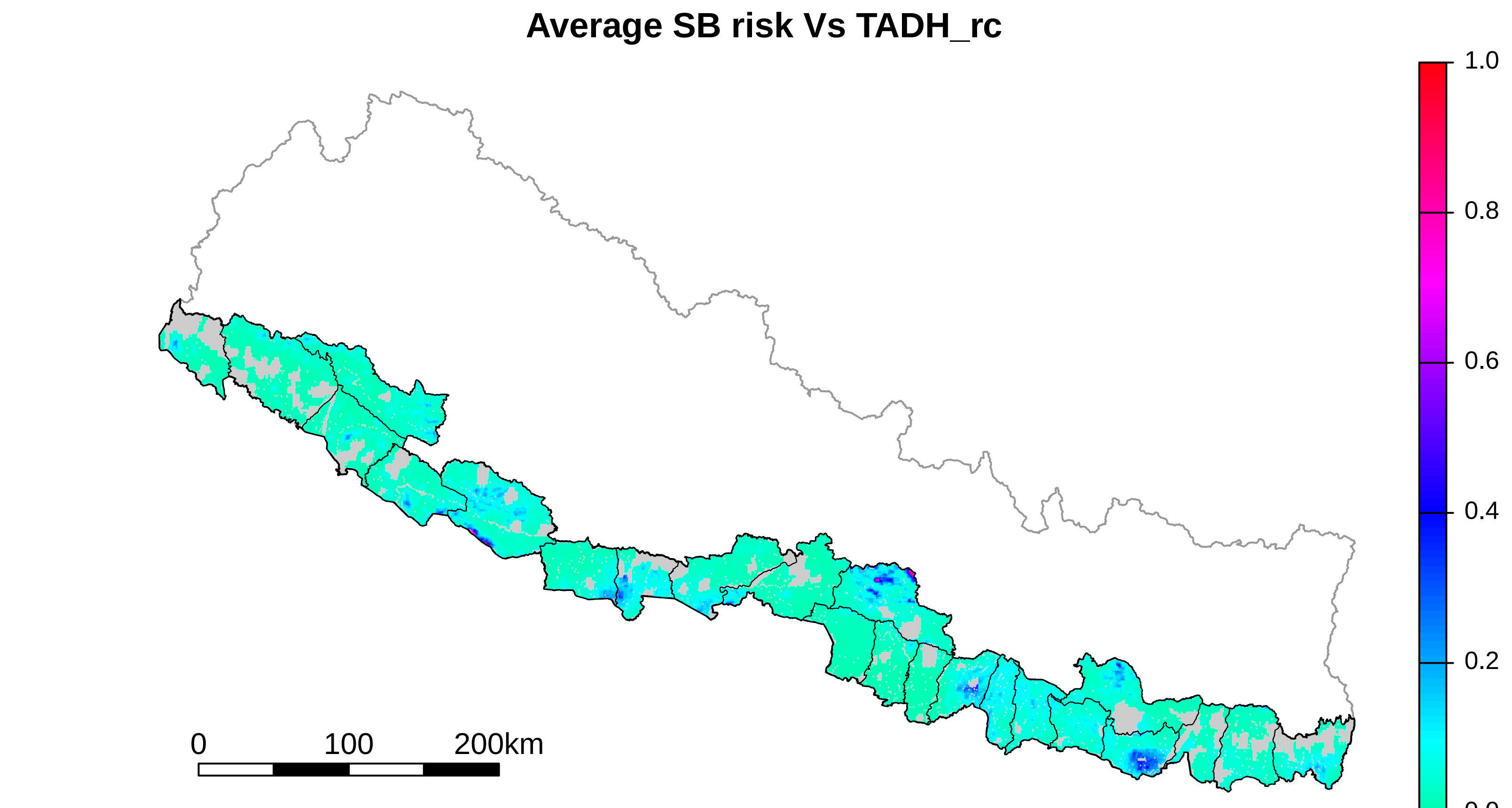
Average SB risk Vs TADH_rc
0
100
200km
1.0
0.8
0.6
0.4
0.2
0.0

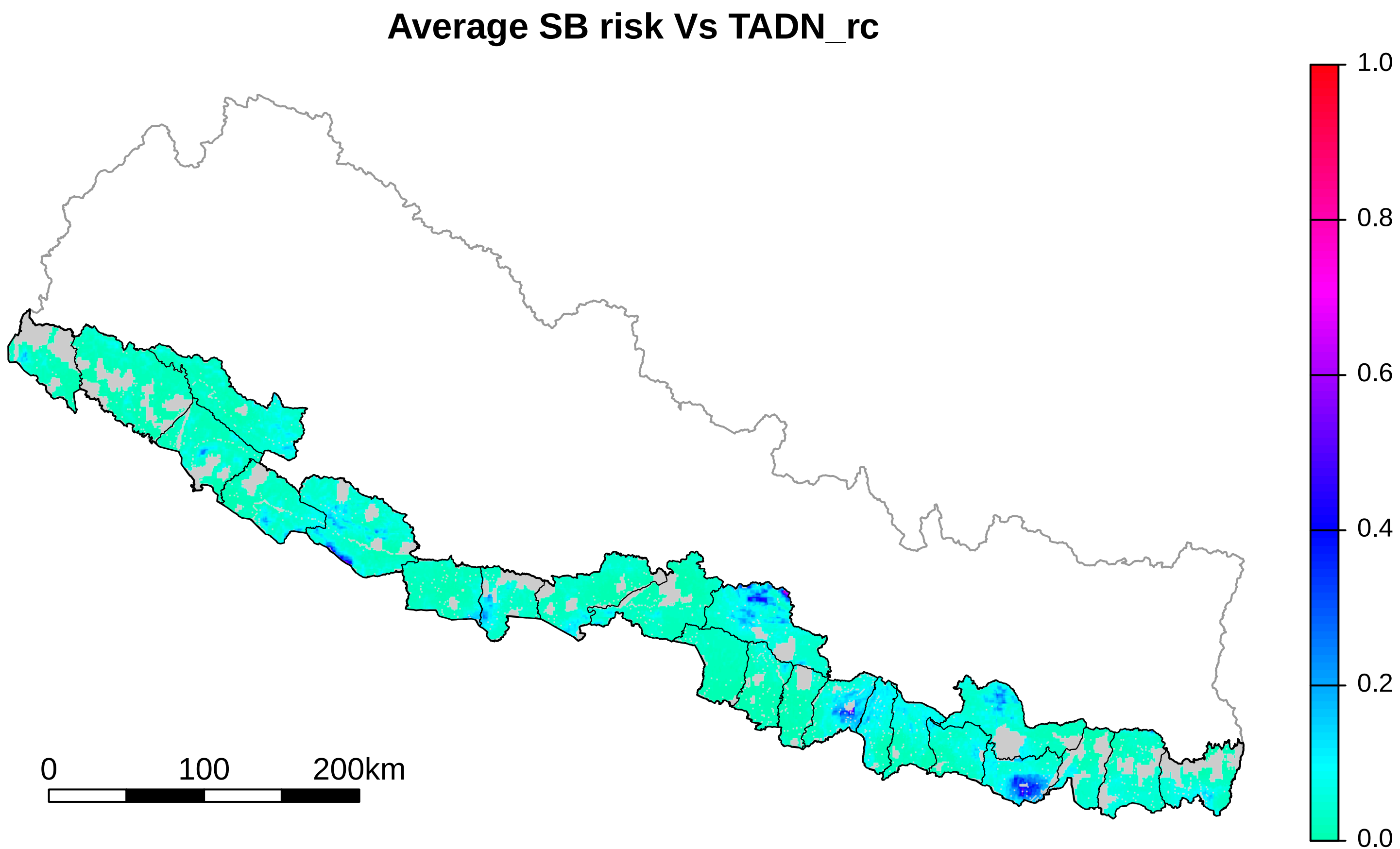

Average SB risk Vs TADN_rc
1.0
0.8
0.6
0.4
0.2
0.0
0
100
200km

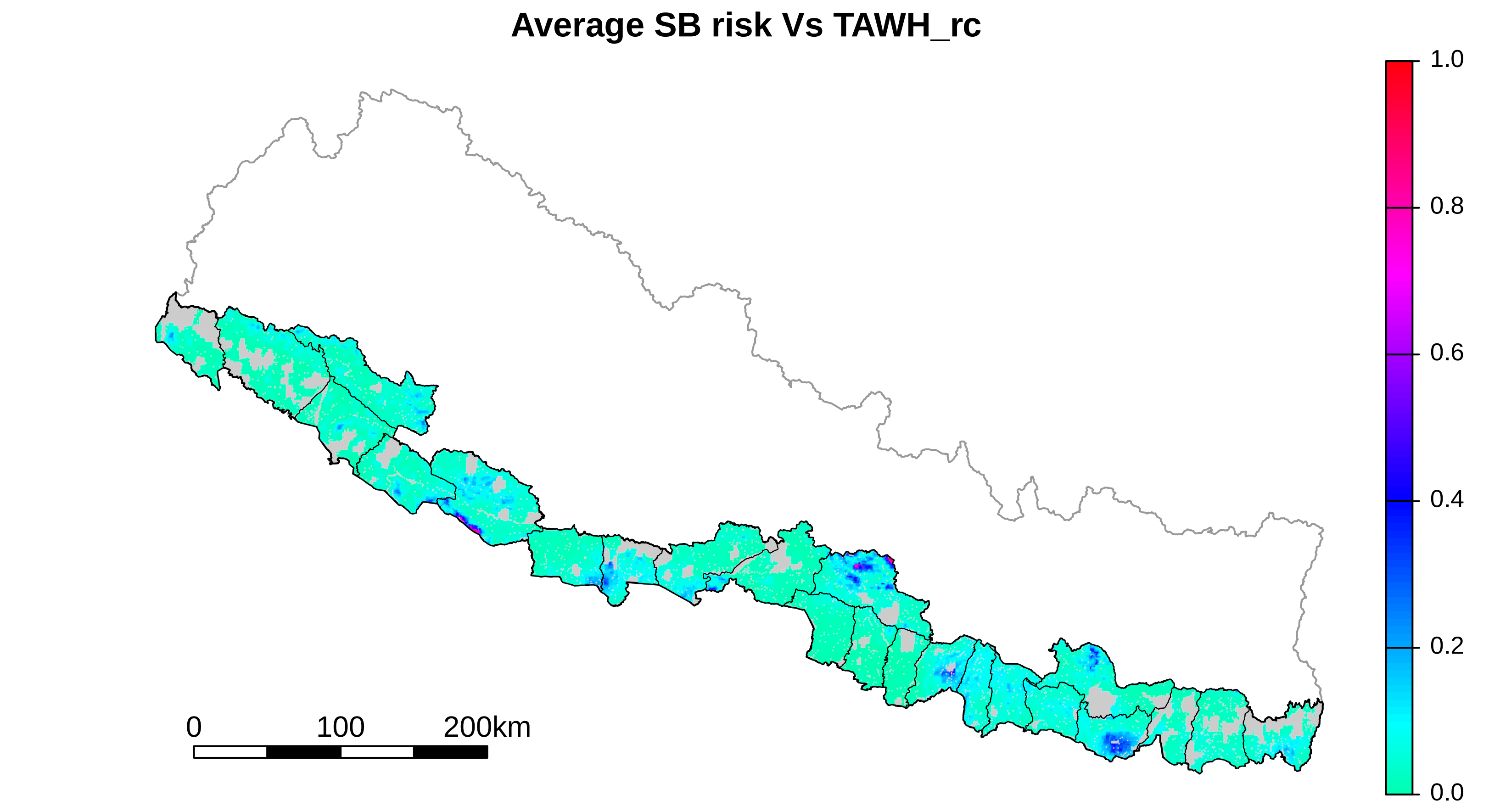

Average SB risk Vs TAWH_rc
0
100
200km
1.0
0.8
0.6
0.4
0.2
0.0

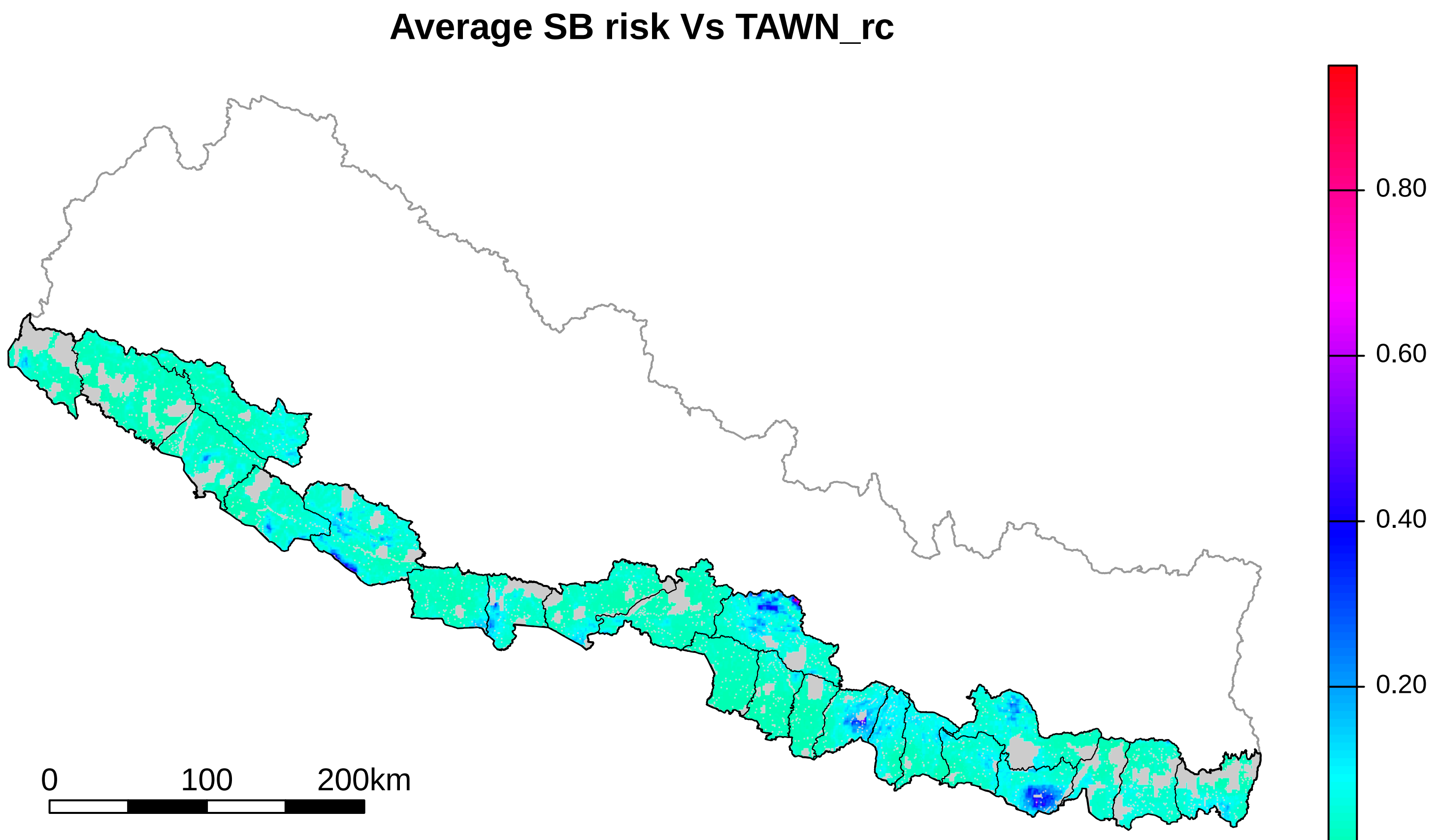
Average SB risk Vs TAWN_rc
0
100
200km
0.80
0.60
0.40
0.20
0.00

Low SB risk Vs FUDH_rc

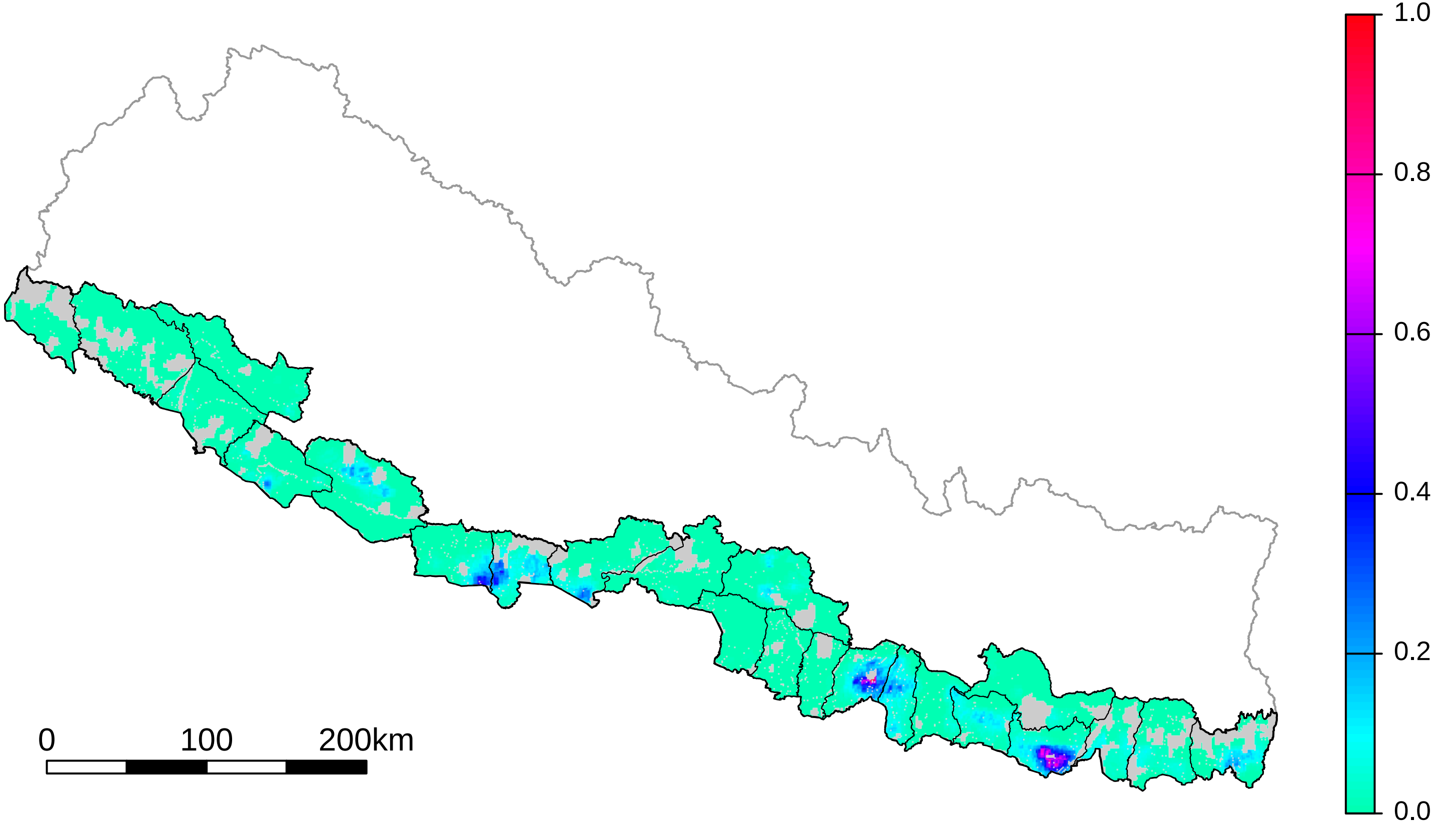

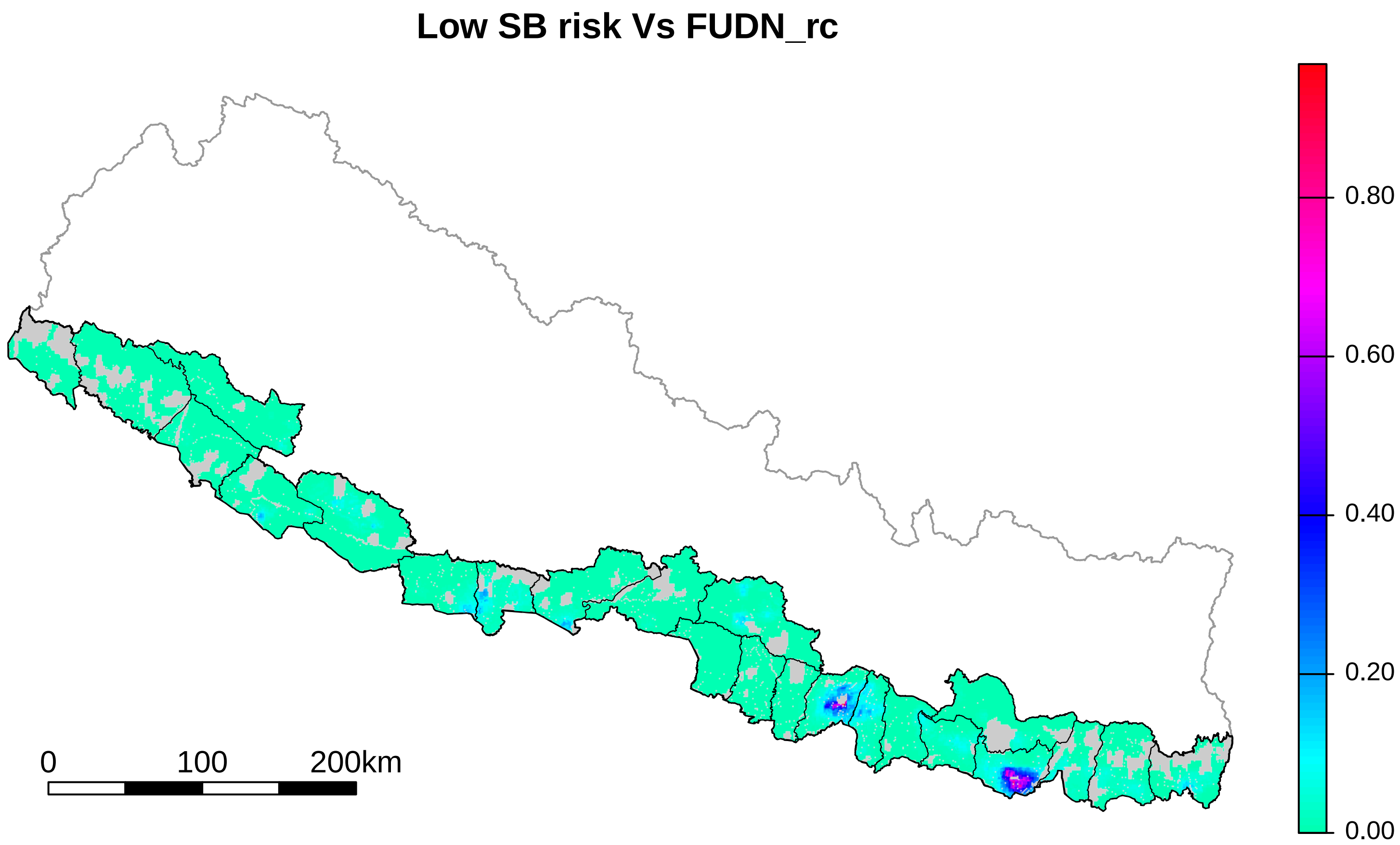

Low SB risk Vs FUDN_rc
0.80
0.60
0.40
0.20
0.00
0
100
200km

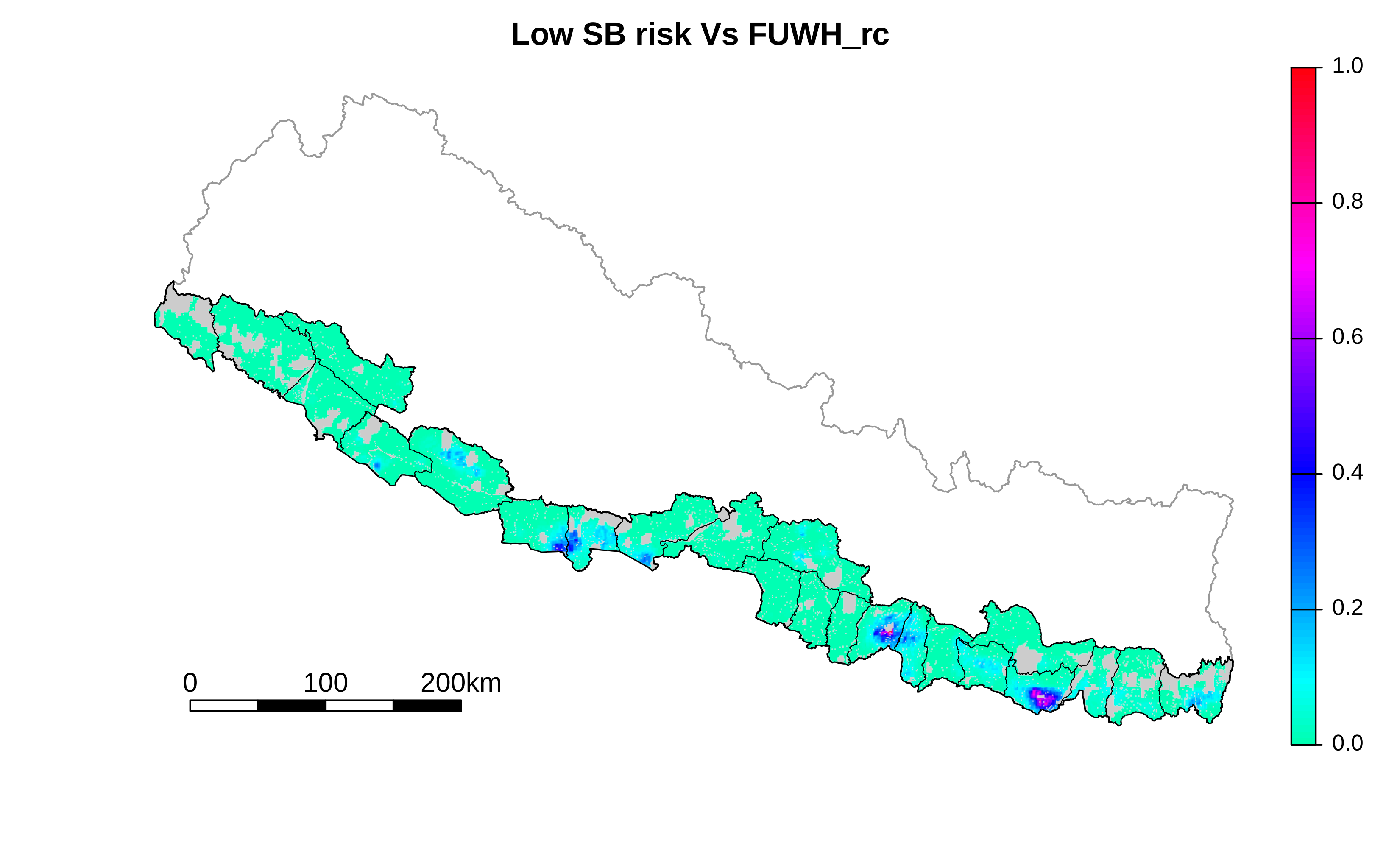
Low SB risk Vs FUWH_rc
0
100
200km
1.0
0.8
0.6
0.4
0.2
0.0

Low SB risk Vs FUWN_rc

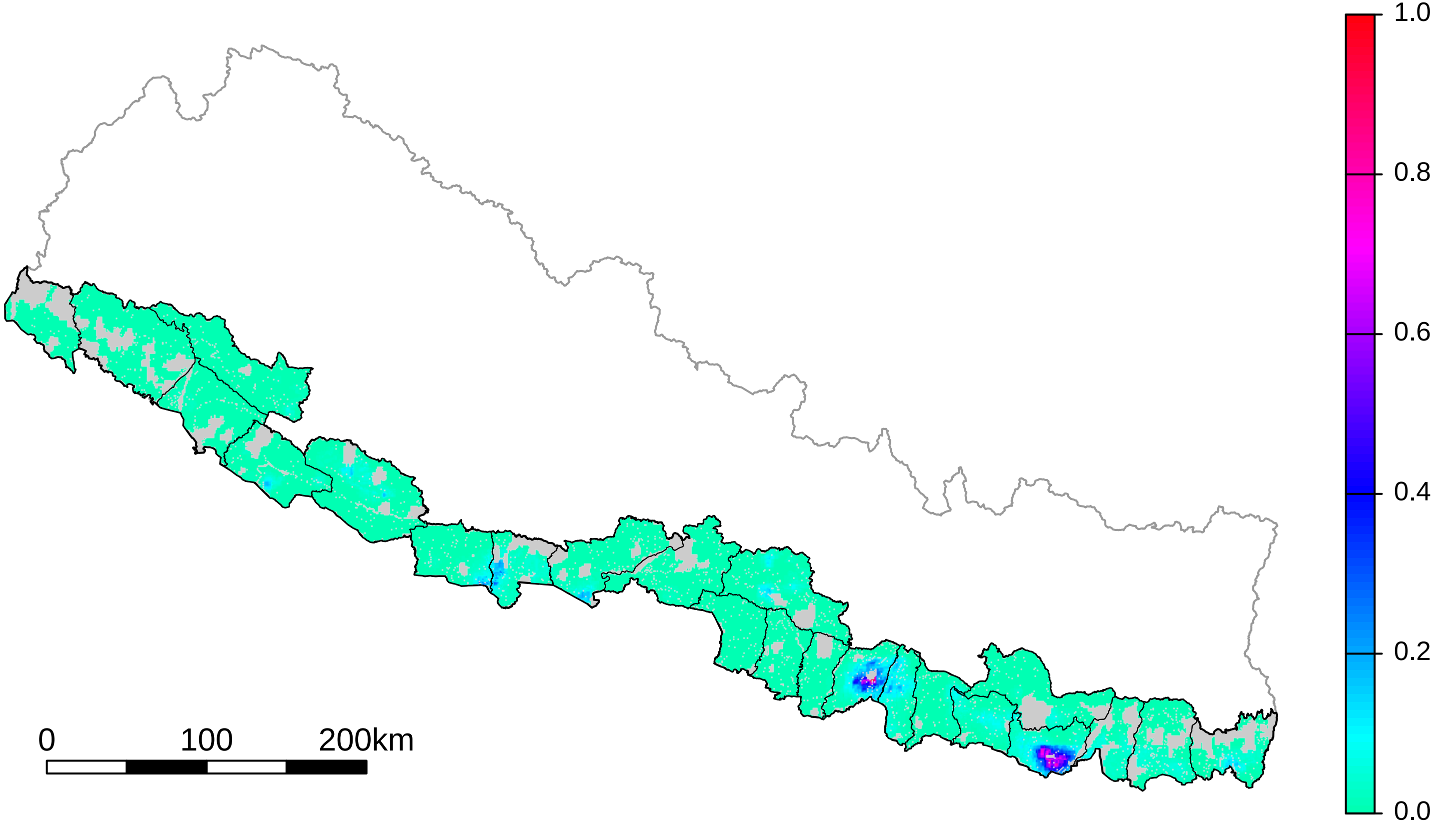

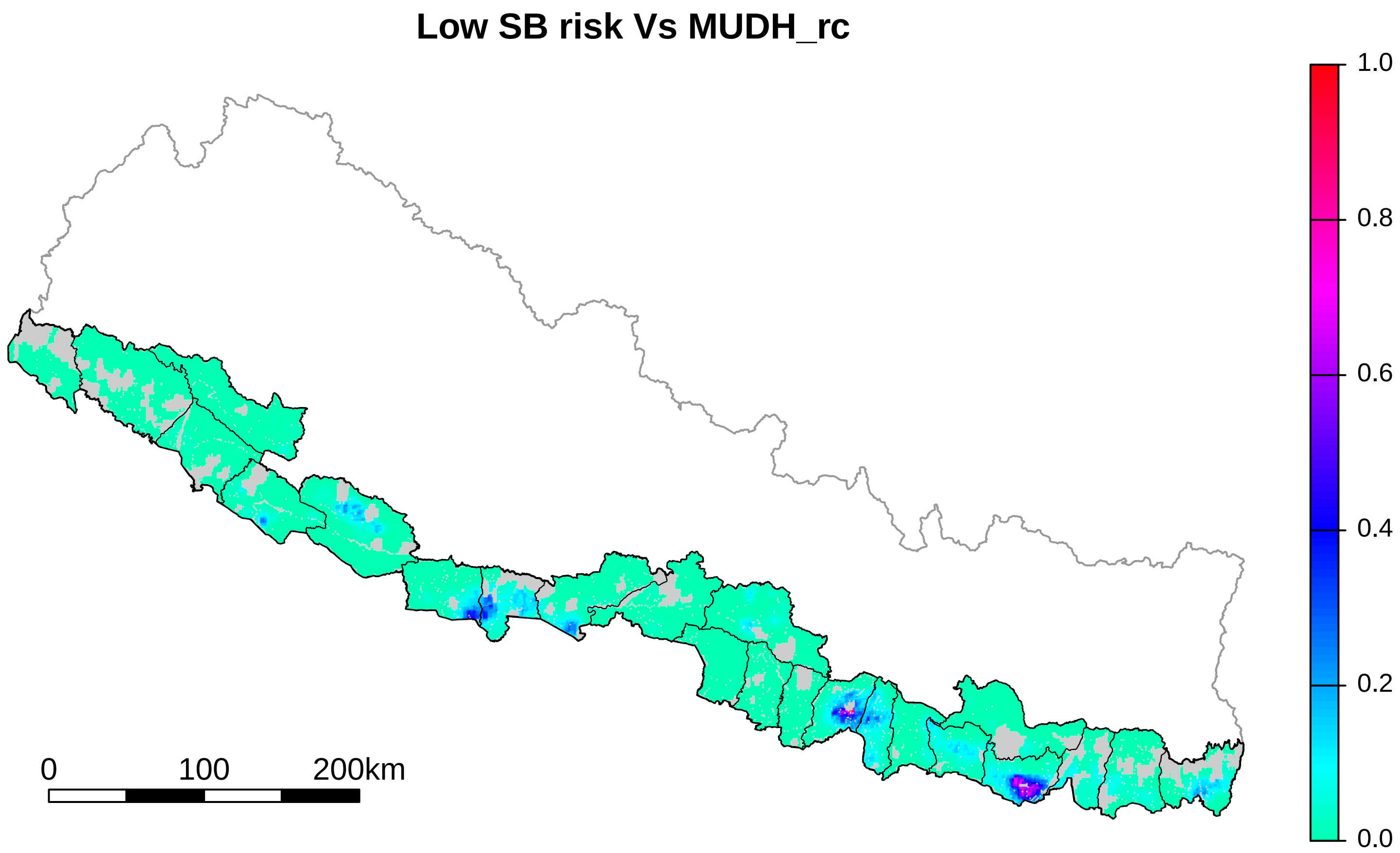

Low SB risk Vs MUDH_rc
0
100
200km
1.0
0.8
0.6
0.4
0.2
0.0

Low SB risk Vs MUDN_rc

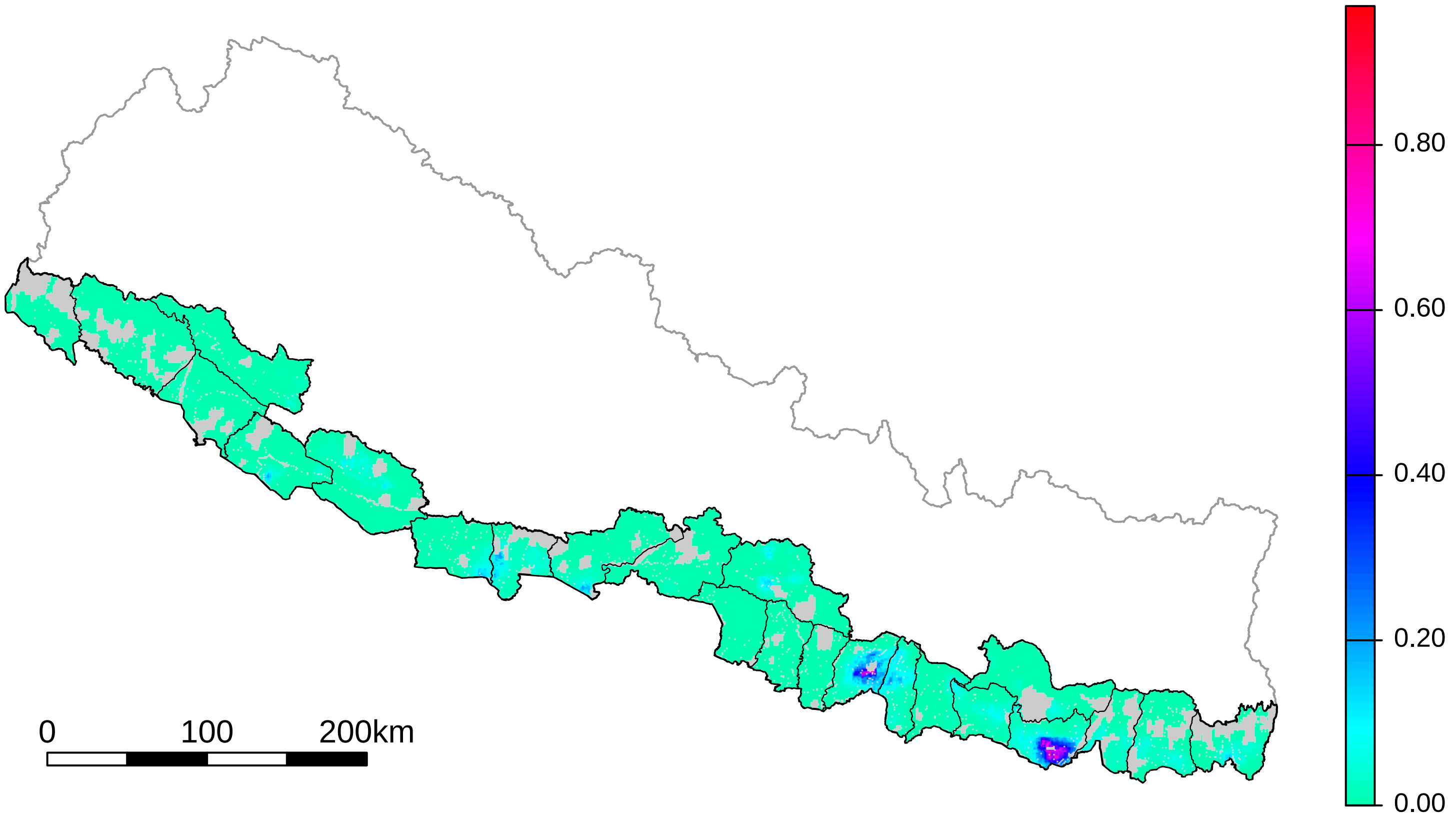

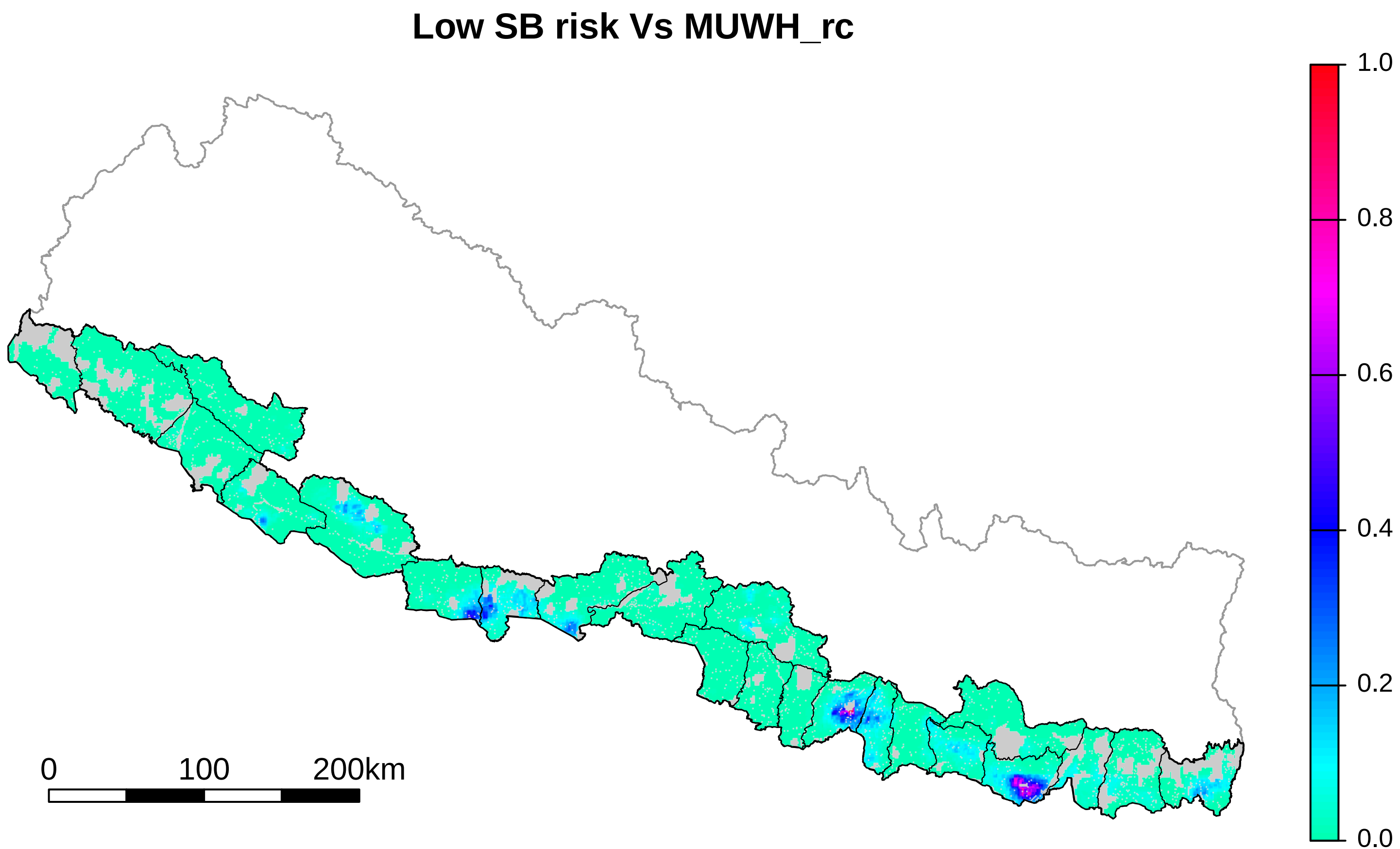
Low SB risk Vs MUWH_rc
1.0
0.8
0.6
0.4
0.2
0.0
0
100
200km

Low SB risk Vs MUWN_rc

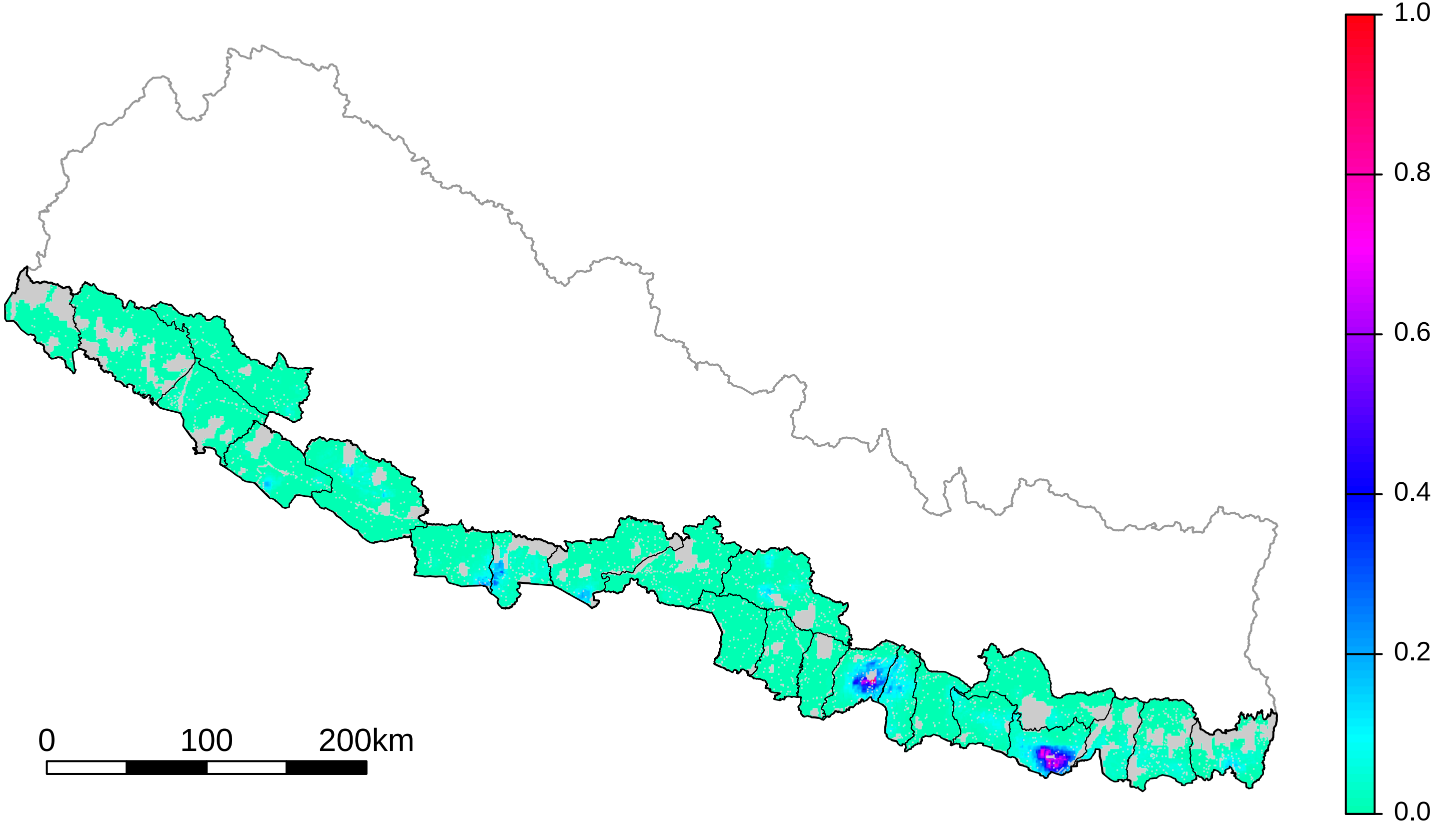

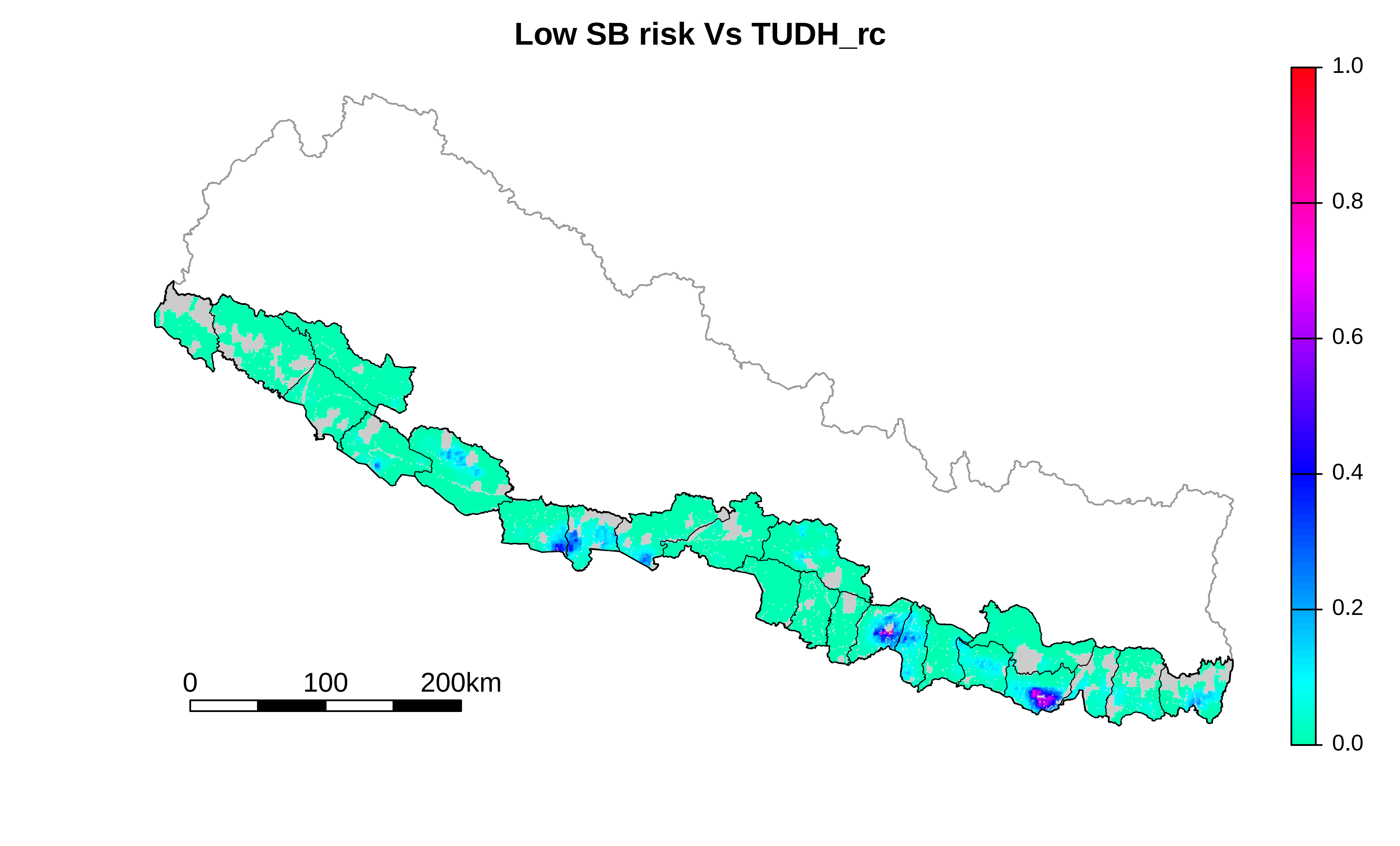

Low SB risk Vs TUDH_rc
0
100
200km
1.0
0.8
0.6
0.4
0.2
0.0

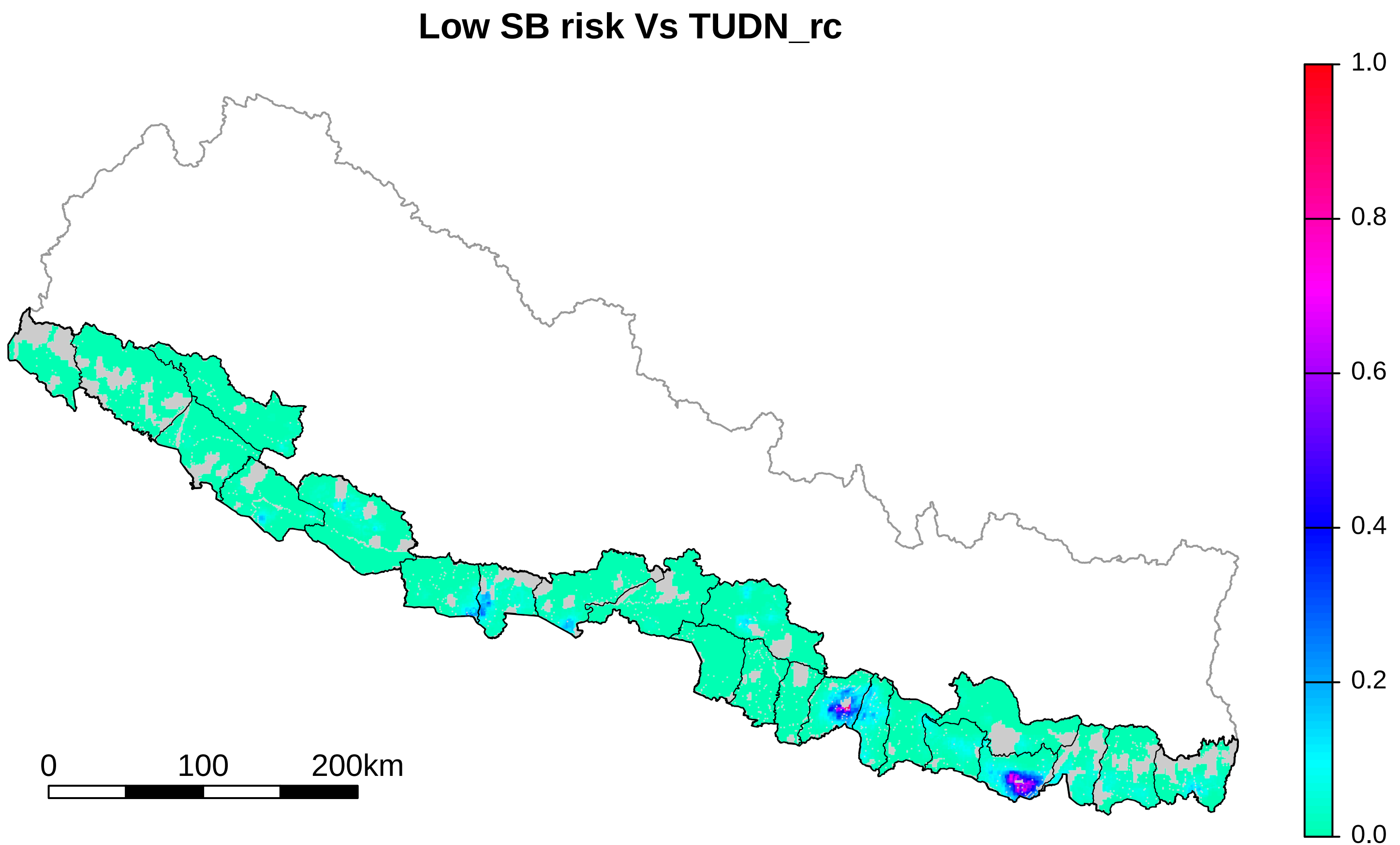
Low SB risk Vs TUDN_rc
1.0
0.8
0.6
0.4
0.2
0.0
0
100
200km

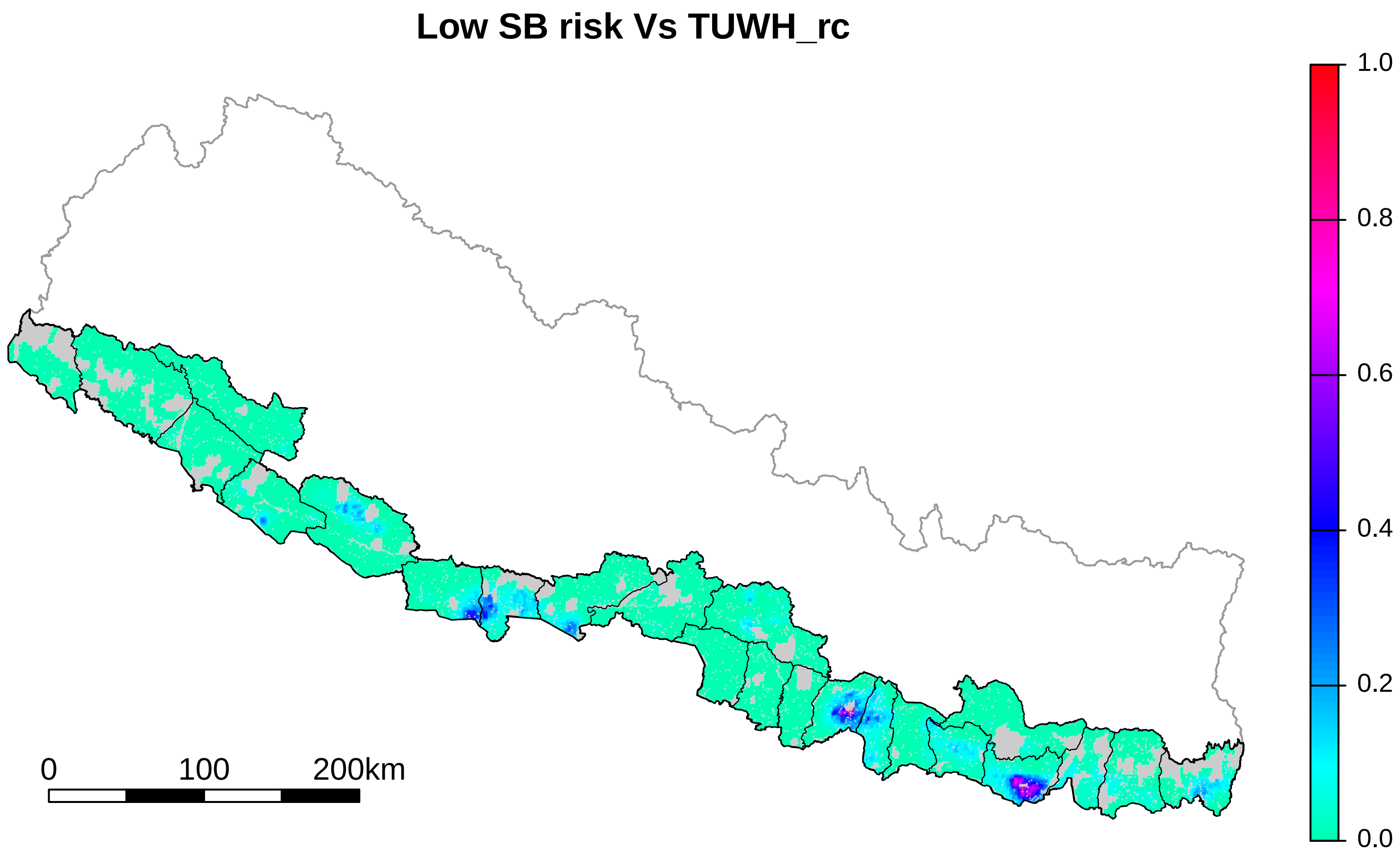
Low SB risk Vs TUWH_rc
1.0
0.8
0.6
0.4
0.2
0.0
0
100
200km

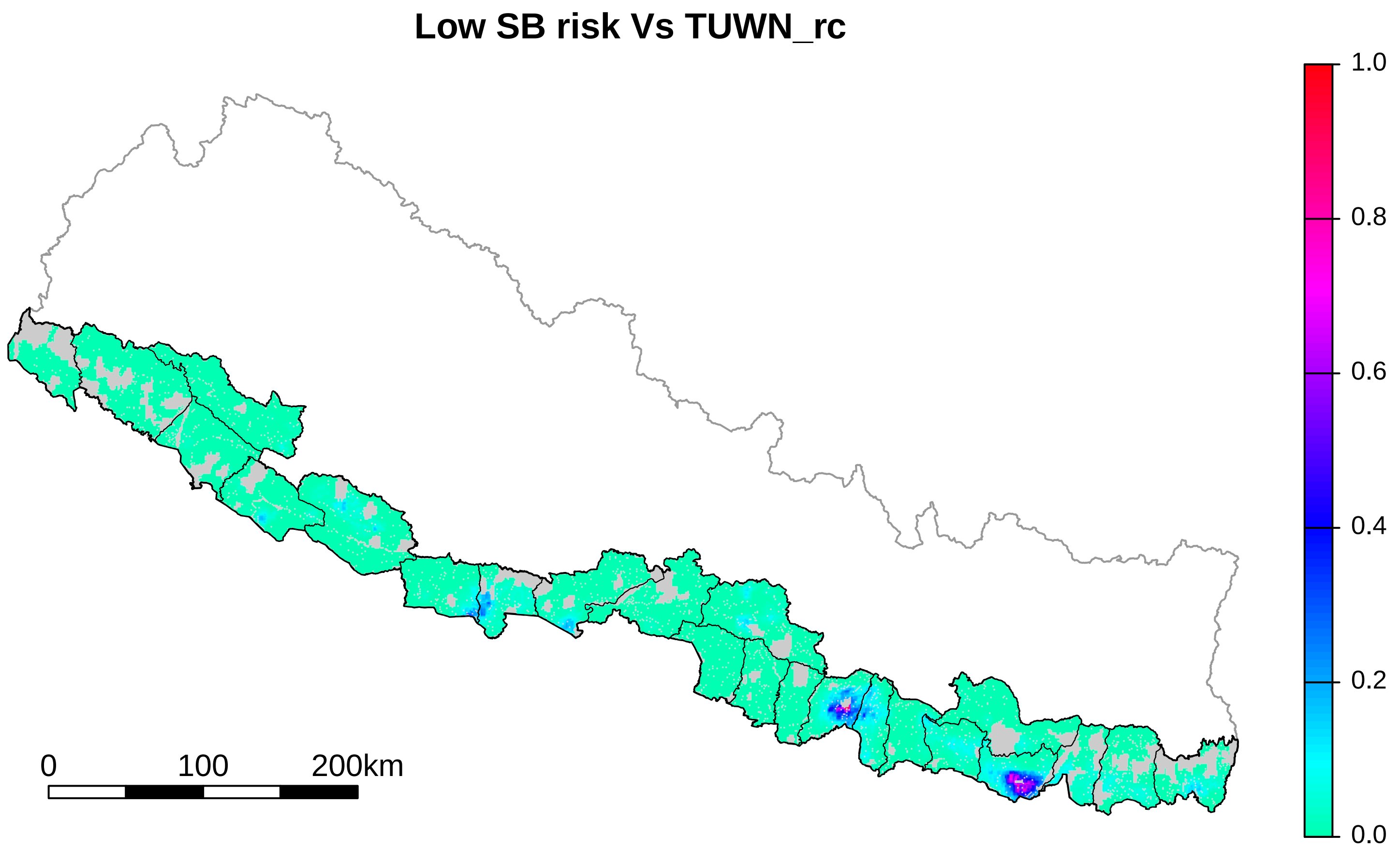
Low SB risk Vs TUWN_rc
1.0
0.8
0.6
0.4
0.2
0.0
0
100
200km
