# Supplementary material for: Vulnerability to snakebite envenoming and access to healthcare in the Terai region of Nepal: a geospatial analysis
Source: Lancet Reg Health Southeast Asia. 2022 Nov 17;9:100103. doi: 10.1016/j.lansea.2022.100103 (PMC10306013; doi:10.1016/j.lansea.2022.100103)
Supplement: Caption for Supplementary Material [file mmc7.docx]

Appendix 1: Methodological details, travel scenarios, and supplementary tables and figures.

Appendix 2: Tables of population coverage per district and accessibility scenarios.

Appendix 3: Normalized-continuous vulnerability maps for each scenario.

Appendix 4: Interactive choropleth map of the rural population by municipality, in the high snakebite envenoming vulnerability class for a medium vulnerability scenario

Appendix 5: Interactive choropleth map of the rural population by municipality, in the medium snakebite envenoming vulnerability class for a medium vulnerability scenario

Appendix 6: Interactive choropleth map of the rural population by municipality, in the low snakebite envenoming vulnerability class for a medium vulnerability scenario
